# Synthesis and Bioactivity of Novel Sulfonate Scaffold-Containing Pyrazolecarbamide Derivatives as Antifungal and Antiviral Agents

**DOI:** 10.3389/fchem.2022.928842

**Published:** 2022-06-22

**Authors:** Zhi-Wei Lei, Jianmei Yao, Huifang Liu, Chiyu Ma, Wen Yang

**Affiliations:** ^1^ State Key Laboratory Breeding Base of Green Pesticide and Agricultural Bioengineering, Key Laboratory of Green Pesticide and Agricultural Bioengineering, Ministry of Education, Guizhou University, Guiyang, China; ^2^ Tea Research Institute, Guizhou Academy of Agricultural Sciences, Guiyang, China

**Keywords:** pyrazolecarbamide, sulfonate, antifungal activity, antiviral activity, synthesis

## Abstract

Novel pyrazolecarbamide derivatives bearing a sulfonate fragment were synthesized to identify potential antifungal and antiviral agents. All the structures of the key intermediates and target compounds were confirmed by nuclear magnetic resonance (NMR) and high-resolution mass spectrometry (HRMS). The single-crystal X-ray diffraction of the compound **T22** showed that pyrazole carbamide is a sulfonate. The *in vitro* antifungal activities of the target compounds against Colletotrichum camelliae, Pestalotiopsis theae, Gibberella zeae, and Rhizoctonia solani were evaluated at 50 μg/ml. Among the four pathogens, the target compounds exhibited the highest antifungal activity against Rhizoctonia solani. The compound **T24** (EC_50_ = 0.45 mg/L) had higher antifungal activity than the commercial fungicide hymexazol (EC_50_ = 10.49 mg/L) against R. solani, almost similar to bixafen (EC_50_ = 0.25 mg/L). Additionally, the target compounds exhibited protective effects *in vivo* against TMV. Thus, this study reveals that pyrazolecarbamide derivatives bearing a sulfonate fragment exhibit potential antifungal and antiviral activities.

## Graphical Abstract

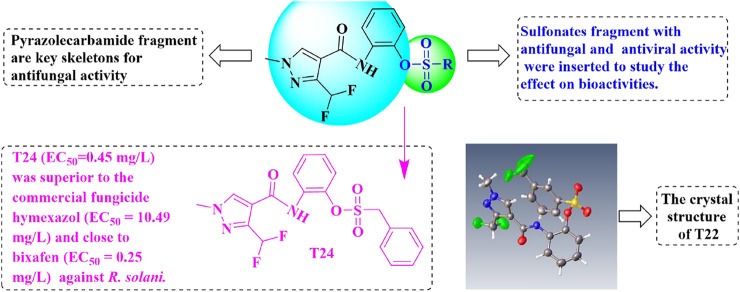

## Introduction

Phytopathogenic microorganisms, such as Rhizoctonia solani, Gibberella zeae, Pestalotiopsis theae, Colletotrichum camelliae, and tobacco mosaic virus (TMV) reduce the yield and quality of food and cash crops (Fisher et al., 2012). Chemical pesticides are still the most commonly used control measure for these diseases; however, the associated pesticide resistance and environmental hazards (Wei et al., 2020) impede their usage. Therefore, there is an urgent need to develop novel eco-friendly antifungal and antiviral agents agent with low toxicity and high efficiency.

Pyrazole and its derivatives have received considerable attention because of their diverse agrochemical and pharmaceutical applications. Most pyrazole derivatives exhibit a broad spectrum of biological activities, including antifungal (Kanungo and Joshi, 2014; Mu et al., 2016; Yan et al., 2018), insecticidal (Wu et al., 2012; Jiang et al., 2020), antibacterial (El Shehry et al., 2018; Wang et al., 2021), and other antimicrobial activities (Kasiotis et al., 2014; Saleh et al., 2020). Especially, pyrazole carboxamide derivatives, such as penthiopyrad, furametpyr, penflufen, isopyrazam, and bixafen, which could inhibit the succinate dehydrogenase, have been developed and commercialized as fungicides (Si et al., 2019).

Sulfonates are also widely applied in agrochemical and medical industries because of their insecticidal (Sun et al., 2013; Wang et al., 2015), antifungal (Kang et al., 2019; Zhou et al., 2022), and antibacteria (Su et al., 2021) Moreover, the heterocyclic compounds containing aryl sulfonate moiety exhibit excellent antiviral activities (Zeng et al., 2010; Huang et al., 2015; Hadházi et al., 2017).

Therefore, we designed and synthesized a series of novel pyrazolecarbamide derivatives bearing a sulfonate moiety based on the active splicing principle and used the mycelial growth rate and half-leaf blight spot methods to evaluate their antifungal and antiviral activities.

## Materials and Methods

### Chemistry

The ^1^H and ^13^C NMR spectra were recorded in CDCl_3_ using 400 and 101 MHz spectrophotometers (Bruker BioSpin GmbH, Rheinstetten, Germany), respectively, while high-resolution mass spectrometry (HRMS) was performed using Thermo Scientific Q Exactive (Thermo Fisher Scientific, Massachusetts, America). The X-ray crystallographic data were collected and processed on a D8 Quest X-ray diffractometer (Bruker BioSpin GmbH, Rheinstetten, German). All solvents were dried using the standard methods and distilled before use.

### 3-(Difluoromethyl)-1-Methyl-1H-Pyrazole-4-Carboxylic Acid (4)

**SCHEME 1:**
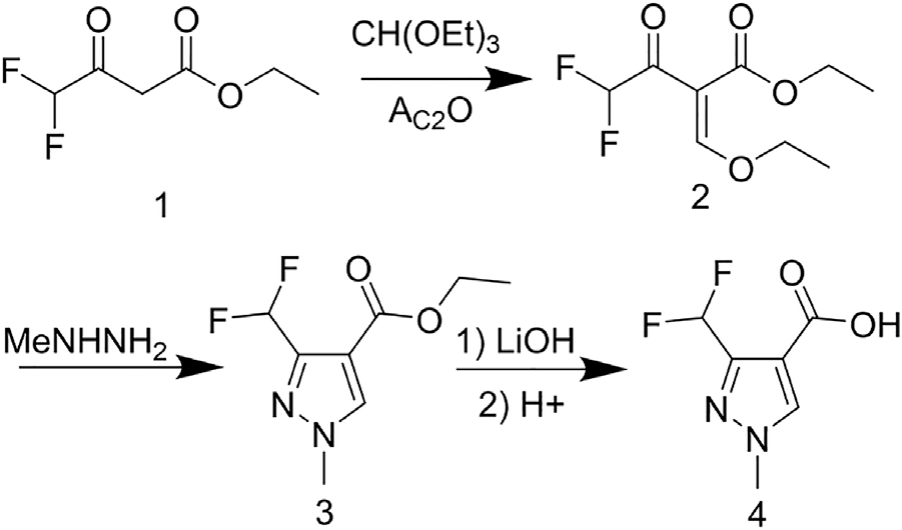
Synthesis of the key intermediate **4**.

As shown in Scheme 1, the key intermediate **4** was synthesized using a previously published three-step procedure (Wang et al., 2020). White powder, yield 46%. m.p 201.1-201.9°C.^1^H NMR (400 MHz, CDCl_3_) *δ* 7.98 (s, 1H), 7.12 (t, J = 54.3 Hz, 1H), 4.02 (s, 3H). HRMS (ESI): calculated for C_6_H_6_F_2_N_2_O_2_ [M + Na]^+^: 199.02950, found: 199.02896.

### 2-(Difluoromethyl)-N-(2-Hydroxyphenyl)-1-Methyl-1H-Pyrazole-4-Carboxamide (6)

A mixture of 1-Ethyl-3-(3-dimethyllaminopropyl)carbodiimide hydrochloride (EDCI, 120 mmol), Intermediate **4** (100 mmol) and o-aminophenol (100 mmol), and dimethylaminopyridine (DMAP, 10 mmol) were dissolved in CH_2_Cl_2_ (500 ml) at −10°C for 1 h. Thereafter, the mixture was stirred at room temperature for 8 h, and the key intermediate **6** was purified using column chromatography. Light yellow solid, yield 62%. m.p. 181.1-182.3°C .^1^H NMR (400 MHz, CDCl_3_) *δ* 8.99 (s, 1H), 8.37 (s, 1H), 8.08 (s, 1H), 7.17 (td, J = 7.7, 1.6 Hz, 1H), 7.05 (ddd, J = 7.8, 6.0, 1.5 Hz, 2H), 6.91 (dd, J = 7.4,1.5 Hz, 1H), 6.88 (t, J = 54.1Hz, 1H), 3.97 (s, 3H). ^13^C NMR (101 MHz, CDCl_3_) *δ* 160.74, 149.12(t, J = 26.5 Hz), 136.77, 127.62, 125.42, 122.63, 120.51, 120.10, 115.5, 112.18, 110.40 (t, J = 235.3 Hz), 39.71. HRMS (ESI): calculated for C_12_H_11_F_2_N_3_O_2_ [M + Na]^+^: 290.07170, found: 290.07126.

### General Procedure for the Preparation of the Target Compounds (T1-27)

Catalytic DMAP, arylsulfonyl chloride (1.1 mmol), and Et_3_N (2 mmol) were added to a stirred CH_3_CN (20 ml) solution of the key intermediate **6** (1 mmol), and the reaction was monitored at room temperature using TLC. Thereafter, the solvent was removed by rotary evaporation, and 10 ml of water was added to the residue, followed by extraction of the aqueous layer three times (30 ml × 3) using ethyl acetate. The organic layers were then combined and dried using anhydrous Na_2_SO_4_ and later concentrated under reduced pressure to form a crude product, purified using flash chromatography to obtain the target product.

### 2-(3-(Difluoromethyl)-1-Methyl-1H-Pyrazole-4-Carboxamido)Phenyl Benzenesulfonate (T1)

Gray powder, yield 72%. m.p. 138.3-139.6°C .^1^H NMR (400 MHz, CDCl_3_) *δ* 8.33 (s, 1H), 8.27 (dd, J = 8.3, 1.6 Hz, 1H), 7.92 (s, 1H), 7.87–7.80 (m, 2H), 7.70–7.61 (m, 1H), 7.52–7.45 (m, 2H), 7.26 (dd, J = 15.7, 1.5 Hz, 1H), 7.08 (t, J = 54.1 Hz, 1H), 7.04–6.97 (m, 1H), 6.90 (dd, J = 8.2, 1.5 Hz, 1H), 4.00 (s, 3H).^13^C NMR (101 MHz, CDCl_3_) *δ* 159.33, 144.86(t, J = 26.5 Hz), 139.41, 134.99, 134.50, 133.41, 131.01, 129.44(×2), 128.65(×2), 128.01, 124.78, 123.27, 122.71, 116.68, 110.50 (t, J = 235.3 Hz), 39.92. HRMS (ESI): calculated for C_18_H_15_F_2_N_3_O4S[M + Na]^+^: 430.06490, found: 430.06531.

### 2-(3-(Difluoromethyl)-1-Methyl-1H-Pyrazole-4-Carboxamido)Phenyl 4-Methylbenzenesulfonate (T2)

Light yellow power, yield 79%. m.p. 126.2-126.9°C .^1^H NMR (400 MHz, CDCl_3_) *δ* 8.35 (s, 1H), 8.28 (dd, J = 8.3, 1.6 Hz, 1H), 7.92 (s, 1H), 7.76–7.64 (m, 2H), 7.31–7.21 (m, 4H), 7.09 (t, J = 54.1 Hz, 1H), 7.00 (td, J = 7.9, 1.6 Hz, 1H), 6.88 (dd, J = 8.2, 1.5 Hz, 1H), 4.00 (s, 3H), 2.42 (s, 3H).^13^C NMR (101 MHz, CDCl_3_) *δ* 159.35, 146.34, 145.05(t, J = 29.3 Hz), 139.50, 133.19, 131.54, 131.13, 130.07(×2), 128.72(×2), 127.94, 124.73, 123.20, 122.78, 116.82, 110.44(t, J = 235.8 Hz), 39.92, 21.87. HRMS (ESI): calculated for C_19_H_17_F_2_N_3_O_4_S [M + Na]^+^: 444.08055, found: 444.08109.

### 2-(3-(Difluoromethyl)-1-Methyl-1H-Pyrazole-4-Carboxamido)Phenyl 2-Fluorobenzenesulfonate (T3)

White powder, yield 78%. m.p. 123.9-124.5°C.^1^H NMR (400 MHz, CDCl_3_) *δ* 8.40 (s, 1H), 8.33 (dd, J = 8.3, 1.6 Hz, 1H), 7.96 (s, 1H), 7.89 (ddd, J = 8.3, 6.9, 1.8 Hz, 1H), 7.76–7.66 (m, 1H), 7.30 (qd, J = 7.7, 1.3 Hz, 3H), 7.26–7.19 (m, 2H), 7.12 (t, J = 54.0 Hz, 1H), 7.15 (dd, J = 8.2, 1.5 Hz, 1H), 7.10-7.02 (m, 1H), 4.02 (s, 3H).^13^C NMR (101 MHz, CDCl_3_) *δ* 160.92, 159.53, 158.34, 138.73, 137.67, 137.58, 132.71, 131.57, 131.09, 128.31, 125.00, 124.96, 124.88, 123.24, 122.66, 117.81, 117.60, 116.77, 112.53, 110.19, 39.97. HRMS (ESI): calculated for C_18_H_14_F_3_N_3_O_4_S [M + Na]^+^: 448.05548, found: 448.05454.

### 2-(3-(Difluoromethyl)-1-Methyl-1H-Pyrazole-4-Carboxamido)Phenyl 3-Fluorobenzenesulfonate (T4)

Gray powder, yield 76%. m.p. 119.3-120.9°C.^1^H NMR (400 MHz, CDCl_3_) *δ* 8.31–8.19 (m, 2H), 7.92 (s, 1H), 7.63–7.53 (m, 2H), 7.47 (td, J = 8.1, 5.2 Hz, 1H), 7.33 (tdd, J = 8.3, 2.5, 1.0 Hz, 1H), 7.30–7.27 (m, 1H), 7.05 (ddd, J = 8.7, 7.2, 1.5 Hz, 1H),7.02 (t, J = 54.1 Hz, 1H), 7.00 (dd, J = 8.2, 1.7 Hz, 1H), 3.99 (s, 3H).^13^C NMR (101 MHz, CDCl_3_) *δ* 163.61, 161.09, 159.26, 144.67, 144.40, 144.13, 139.44, 136.61, 136.53, 133.98, 131.37, 131.30, 130.85, 128.18, 124.96, 124.58, 124.54, 123.55, 122.56, 122.35, 122.14, 116.55, 116.08, 115.83, 113.16, 110.82, 108.49, 39.86. HRMS (ESI): calculated for C_18_H_14_F_3_N_3_O_4_S [M + Na]^+^:448.05548, found: 448.05454.

### 2-(3-(Difluoromethyl)-1-Methyl-1H-Pyrazole-4-Carboxamido)Phenyl 4-Fluorobenzenesulfonate (T5)

Light yellow powder, yield 69%. m.p. 165.2-165.9°C.^1^H NMR (400 MHz, CDCl_3_) *δ* 8.32–8.21 (m, 2H), 7.93 (s, 1H), 7.88–7.80 (m, 2H), 7.33–7.26 (m, 1H), 7.18–7.07 (m, 2H), 7.05 (ddd, J = 8.9, 7.4, 1.6 Hz, 1H),7.00 (t, J = 54.0 Hz, 1H), 6.98 (dd, J = 8.3, 1.6 Hz, 1H), 4.00 (s, 3H).^13^C NMR (101 MHz, CDCl_3_) *δ* 166.37(d, J = 259.6 Hz), 159.22, 144.15(t, J = 26.2 Hz), 144.18, 139.45, 134.24, 131.69, 131.59, 130.89, 130.69, 128.13, 124.92, 123.48, 122.78, 116.96, 116.73, 116.65, 110.90(t, J = 235.8 Hz), 105.41, 39.90. HRMS (ESI): calculated for C_18_H_14_F_3_N_3_O_4_S [M + Na]^+^:448.05548, found: 448.05454.

### 2-(3-(Difluoromethyl)-1-Methyl-1H-Pyrazole-4-Carboxamido)Phenyl 2-Chlorobenzenesulfonate (T6)

Gray powder, yield 70%. m.p. 116.3-117.2°C.^1^H NMR (400 MHz, CDCl_3_) *δ* 8.48 (s, 1H), 8.39–8.30 (m, 1H), 8.03 (dd, J = 8.0, 1.5 Hz, 1H), 7.97 (s, 1H), 7.67–7.57 (m, 2H), 7.44 (ddd, J = 8.0, 7.1, 1.6 Hz, 1H), 7.29 (ddd, J = 8.6, 5.6, 3.4 Hz, 1H), 7.11 (d, J = 54.1 Hz, 1H), 7.06–6.99 (m, 2H), 4.01 (s, 3H).^13^C NMR (101 MHz, CDCl_3_) *δ* 159.59, 145.46(t, J = 26.5 Hz), 139.00, 135.92, 133.48, 133.15, 132.92, 132.54, 132.50, 131.21, 128.26, 127.52, 124.89, 123.48, 122.61, 116.70, 110.23 (t, J = 236.3 Hz), 39.96. HRMS (ESI): calculated for C_18_H_14_ClF_2_N_3_O_4_S [M + Na]^+^: 464.02593, found: 464.02521.

### 2-(3-(Difluoromethyl)-1-Methyl-1H-Pyrazole-4-Carboxamido)Phenyl 3-Chlorobenzenesulfonate (T7)

Light yellow powder, yield 73%. m.p. 110.0-111.9°C.^1^H NMR (400 MHz, CDCl_3_) *δ* 8.24 (dd, J = 8.2, 1.4 Hz, 1H), 8.19 (s, 1H), 7.91 (s, 1H), 7.86 (t, J = 1.9 Hz, 1H), 7.63 (dt, J = 7.9, 1.4 Hz, 1H), 7.58 (ddd, J = 8.1, 2.1, 1.0 Hz, 1H), 7.41 (t, J = 8.0 Hz, 1H), 7.29 (ddd, J = 8.5, 6.6, 2.4 Hz, 1H), 7.11–7.02 (m, 2H), 7.01 (d, J = 54.1 Hz, 1H), 3.99 (s, 3H).^13^C NMR (101 MHz, CDCl_3_) *δ* 159.16, 144.30(t, J = 27.3 Hz), 139.40, 136.43, 135.79, 134.99, 134.11, 130.81, 130.70, 128.47, 128.22, 126.77, 124.98, 123.52, 122.72, 116.53, 110.90(t, J = 235.3 Hz), 39.88. HRMS (ESI): calculated for C_18_H_14_ClF_2_N_3_O_4_S [M + Na]^+^: 464.02593, found: 464.02521.

### 2-(3-(Difluoromethyl)-1-Methyl-1H-Pyrazole-4-Carboxamido)Phenyl 4-Chlorobenzenesulfonate (T8)

Light yellow powder, yield 79%. m.p. 185.6-185.9°C.^1^H NMR (400 MHz, CDCl_3_) *δ* 8.24 (dd, J = 8.2, 1.5 Hz, 2H), 8.22 (s, 1H), 7.93 (s, 1H), 7.77–7.69 (m, 2H), 7.44–7.36 (m, 2H), 7.29 (ddd, J = 8.6, 7.2, 1.8 Hz, 1H), 7.07 (td, J = 7.7, 1.5 Hz, 2H), 7.02 (dd, J = 8.2, 1.8 Hz, 1H), 6.98 (t, J = 54.1 Hz, 1H), 3.99 (s, 3H).^13^C NMR (101 MHz, CDCl_3_) *δ* 159.15, 143.97(t, J = 26.7 Hz), 141.62, 139.54, 134.48, 133.37, 130.80, 130.04(×2), 129.75(×2), 128.15, 125.00, 123.59, 122.89, 116.64, 111.03(t, J = 234.7 Hz), 39.88. HRMS (ESI): calculated for C_18_H_14_ClF_2_N_3_O_4_S [M + Na]^+^: 464.02593, found: 464.02521.

### 2-(3-(Difluoromethyl)-1-Methyl-1H-Pyrazole-4-Carboxamido)Phenyl 2-Bromobenzenesulfonate (T9)

Gray powder, yield 69%. m.p. 133.9-134.2°C.^1^H NMR (400 MHz, CDCl_3_) *δ* 8.48 (s, 1H), 8.36–8.28 (m, 1H), 8.04 (dd, J = 7.8, 1.9 Hz, 1H), 7.98 (s, 1H), 7.81 (dd, J = 7.8, 1.4 Hz, 1H), 7.53 (td, J = 7.6, 1.9 Hz, 1H), 7.48 (td, J = 7.7, 1.4 Hz, 1H), 7.32–7.25 (m, 1H), 7.00 (t, J = 54.0 Hz, 1H), 7.04–6.98 (m, 2H), 4.00 (s, 3H).^13^C NMR (101 MHz, CDCl_3_) *δ* 159.59, 145.42(t, J = 25.8 Hz), 139.06, 136.05, 135.79, 134.98, 132.99, 132.79, 131.24, 128.23, 128.07, 124.89, 123.52, 122.66, 121.38, 116.82, 110.22(t, J = 235.6 Hz), 39.94. HRMS (ESI): calculated for C_18_H_14_BrF_2_N_3_O_4_S [M + Na]^+^: 507.97542, found: 507.97227.

### 2-(3-(Difluoromethyl)-1-Methyl-1H-Pyrazole-4-Carboxamido)Phenyl 3-Bromobenzenesulfonate (T10)

Light yellow powder, yield 80%. m.p. 128.4-128.5°C.^1^H NMR (400 MHz, CDCl_3_) *δ* 8.30–8.21 (m, 1H), 8.18 (s, 1H), 8.01 (t, J = 1.9 Hz, 1H), 7.91 (s, 1H), 7.73 (ddd, J = 8.1, 1.9, 1.0 Hz, 1H), 7.66 (ddd, J = 7.9, 1.8, 1.0 Hz, 1H), 7.33 (t, J = 8.0 Hz, 1H), 7.29 (td, J = 6.1, 3.3 Hz, 1H), 7.11–7.03 (m, 2H), 7.01 (t, J = 54.1 Hz, 1H), 3.99 (s, 3H).^13^C NMR (101 MHz, CDCl_3_) *δ* 159.13, 144.26 (t, J = 27.3 Hz), 139.35, 137.90, 136.47, 134.16, 131.24, 130.87, 130.75, 128.23, 127.18, 125.00, 123.50, 123.38, 122.78, 116.47, 110.91(t, J = 235.3 Hz), 39.91.HRMS (ESI): calculated for C_18_H_14_BrF_2_N_3_O_4_S [M + Na]^+^: 507.97542, found: 507.97227.

### 2-(3-(Difluoromethyl)-1-Methyl-1H-Pyrazole-4-Carboxamido)Phenyl 4-Bromobenzenesulfonate (T11)

Light yellow powder, yield 79%. m.p. 175.8-176.4°C.^1^H NMR (400 MHz, CDCl_3_) *δ* 8.23 (dd, J = 8.2, 1.5 Hz, 1H), 8.20 (s, 1H), 7.94 (s, 1H), 7.68–7.61 (m, 2H), 7.60–7.50 (m, 2H), 7.29 (ddd, J = 8.5, 7.0, 1.9 Hz, 1H), 7.07 (ddd, J = 8.6, 7.1, 1.5 Hz, 1H), 7.03 (dd, J = 8.2, 1.9 Hz, 1H), 6.97 (t, J = 54.1 Hz, 1H), 4.00 (s, 3H).^13^C NMR (101 MHz, CDCl_3_) *δ* 159.12, 143.91(t, J = 28.8 Hz), 139.54, 134.57, 133.94, 132.73(×2), 130.77, 130.26, 130.02(×2), 128.15, 125.02, 123.60, 122.92, 116.60, 111.06(t, J = 235.02 Hz), 39.89.HRMS (ESI): calculated for C_18_H_14_BrF_2_N_3_O_4_S [M + Na]^+^: 507.97542, found: 507.97227.

### 2-(3-(Difluoromethyl)-1-Methyl-1H-Pyrazole-4-Carboxamido)Phenyl 2-Nitrobenzenesulfonate (T12)

Light yellow powder, yield 83%. m.p. 146.0-147.8°C.^1^H NMR (400 MHz, CDCl_3_) *δ* 8.31 (s, 1H), 8.26 (dd, J = 8.3, 1.6 Hz, 1H), 7.92 (dd, J = 7.9, 1.4 Hz, 1H), 7.90 (d, J = 1.2 Hz, 1H), 7.81 (td, J = 7.8, 1.4 Hz, 1H), 7.69 (td, J = 7.8, 1.3 Hz, 1H), 7.66 (dd, J = 7.9, 1.3 Hz, 1H), 7.38 (dd, J = 8.3, 1.5 Hz, 1H), 7.34–7.28 (m, 1H), 7.13 (ddd, J = 8.3, 7.4, 1.6 Hz, 1H),7.11 (t, J = 54.1 Hz, 1H), 4.00 (s, 3H).^13^C NMR (101 MHz, CDCl_3_) *δ* 159.55, 148.37, 146.02(t, J = 25.3 Hz), 138.50, 136.28, 132.83, 132.35(×2), 130.76, 128.57, 128.06, 125.10, 124.90, 123.43, 123.06, 116.02(t, J = 2.7 Hz), 109.75(t, J = 236.8 Hz), 39.91. HRMS (ESI): calculated for C_18_H_14_F_2_N_4_O_6_S [M + Na]^+^: 475.04998, found: 475.04948.

### 2-(3-(Difluoromethyl)-1-Methyl-1H-Pyrazole-3-Carboxamido)Phenyl 4-Nitrobenzenesulfonate (T13)

Gray powder, yield 79%. m.p. 160.4-160.9°C.^1^H NMR (400 MHz, CDCl_3_) *δ* 8.70 (t, J = 2.0 Hz, 1H), 8.42 (ddd, J = 8.2, 2.2, 1.1 Hz, 1H), 8.14 (dd, J = 8.2, 1.6 Hz, 1H), 8.11 (d, J = 4.6 Hz, 1H), 7.99 (dt, J = 8.0, 1.3 Hz, 1H), 7.88 (s, 1H), 7.66 (t, J = 8.1 Hz, 1H), 7.32 (ddd, J = 8.3, 7.5, 1.5 Hz, 1H), 7.26 (dd, J = 8.3, 1.5 Hz, 1H), 7.14 (ddd, J = 8.5, 7.4, 1.6 Hz, 1H), 6.92 (t, J = 54.1 Hz, 1H), 3.98 (s, 3H).^13^C NMR (101 MHz, CDCl_3_) *δ* 158.87, 148.24, 143.29(t, J = 27.7 Hz),, 139.43, 136.92, 135.14, 134.01, 130.78, 130.38, 129.07, 128.44, 125.34, 123.96, 123.77, 122.79, 116.11, 111.46(t, J = 234.2 Hz), 39.83.HRMS (ESI): calculated for C_18_H_14_F_2_N_4_O_6_S [M + Na]^+^: 475.04998, found:475.04948.

### 2-(3-(Difluoromethyl)-1-Methyl-1H-Pyrazole-4-Carboxamido)Phenyl 4-Nitrobenzenesulfonate (T14)

Light yellow powder, yield 80%. m.p. 198.9-199.6°C.^1^H NMR (400 MHz, CDCl_3_) *δ* 8.24–8.17 (m, 2H), 8.15 (dd, J = 8.3, 1.6 Hz, 1H), 8.07 (s, 1H), 7.99–7.94 (m, 2H), 7.86 (s, 1H), 7.33 (td, J = 7.8, 1.6 Hz, 1H), 7.21 (dd, J = 8.3, 1.6 Hz, 1H), 7.14 (ddd, J = 8.5, 7.3, 1.6 Hz, 1H), 6.88 (t, J = 54.1 Hz, 1H), 3.98 (s, 3H).^13^C NMR (101 MHz, CDCl_3_) *δ* 158.82, 151.19, 144.20(t, J = 26.5 Hz), 140.75, 139.69, 135.60, 130.30, 130.00(×2), 128.42, 125.40, 124.41(×2), 124.16, 123.00, 116.31, 111.61(t, J = 234.7 Hz), 39.81. HRMS (ESI): calculated for C_18_H_14_F_2_N_4_O_6_S [M + Na]^+^: 475.04998, found:475.04948.

### 2-(3-(Difluoromethyl)-1-Methyl-1H-Pyrazole-4-Carboxamido)Phenyl 2,5-Dichlorobenzenesulfonate (T15)

Light yellow powder, yield 82%. m.p. 155.6-157.3°C.^1^H NMR (400 MHz, CDCl_3_) *δ* 8.40 (s, 1H), 8.35–8.27 (m, 1H), 8.01 (d, J = 2.4 Hz, 1H), 7.95 (s, 1H), 7.57 (dd, J = 8.6, 2.4 Hz, 1H), 7.52 (d, J = 8.5 Hz, 1H), 7.31 (ddd, J = 8.5, 5.4, 3.5 Hz, 1H), 7.10–7.03 (m, 2H), 7.06 (t, J = 54.1 Hz, 1H), 3.99 (s, 3H).^13^C NMR (101 MHz, CDCl_3_) *δ* 159.52, 145.01(t, J = 26.2 Hz), 138.96, 135.74, 134.44, 133.74, 133.54, 133.36, 132.06, 131.70, 131.01, 128.43, 125.11, 123.77, 122.47, 116.62, 110.43(t, J = 235.3 Hz), 39.93. HRMS (ESI): calculated for C_18_H_13_C_l2_F_2_N_3_O_4_S [M + Na]^+^: 497.98696, found: 497.98602.

### 2-(3-(Difluoromethyl)-1-Methyl-1H-Pyrazole-4-Carboxamido)Phenyl 3,5-Dichlorobenzenesulfonate (T16)

Gray powder, yield 78%. m.p. 128.9-129.5°C.^1^H NMR (400 MHz, CDCl_3_) *δ* 8.27–8.22 (m, 1H), 8.15 (s, 1H), 7.94 (s, 1H), 7.68 (d, J = 1.9 Hz, 2H), 7.56 (t, J = 1.9 Hz, 1H), 7.33 (ddd, J = 8.5, 5.7, 3.3 Hz, 1H), 7.15–7.12 (m, 2H), 7.06 (t, J = 54.0 Hz, 1H), 3.99 (s, 3H).^13^C NMR (101 MHz, CDCl_3_) *δ* 159.03, 143.78(t, J = 25.6 Hz), 139.33, 137.56, 136.50, 134.78, 134.76, 130.61, 128.44, 126.84(×2), 125.19, 123.82, 122.63, 116.40, 111.22(t, J = 234.9 Hz), 76.84, 39.89. HRMS (ESI): calculated for C_18_H_13_C_l2_F_2_N_3_O_4_S [M + Na]^+^: 497.98696, found: 497.98602.

### 2-(3-(Difluoromethyl)-1-Methyl-1H-Pyrazole-4-Carboxamido)Phenyl 3,4-Dichlorobenzenesulfonate (T17)

Gray powder, yield 79%. m.p. 173.4-174.4°C.^1^H NMR (400 MHz, CDCl_3_) *δ* 8.18 (dd, J = 8.2, 1.5 Hz, 1H), 8.11 (d, J = 4.1 Hz, 1H), 7.92 (d, J = 2.4 Hz, 2H), 7.56–7.43 (m, 2H), 7.32 (ddd, J = 8.5, 7.0, 1.9 Hz, 1H), 7.20–7.09 (m, 2H), 6.93 (t, J = 54.1 Hz, 1H), 3.99 (s, 3H).^13^C NMR (101 MHz, CDCl_3_) *δ* 158.92, 143.41(t, J = 28.3 Hz), 139.81, 139.59, 135.04, 134.65, 134.32, 131.42, 130.50, 130.29, 128.31, 127.52, 125.24, 123.89, 123.05, 116.33, 111.39(t, J = 234.8 Hz), 39.84.HRMS (ESI): calculated for C_18_H_13_C_l2_F_2_N_3_O_4_S [M + Na]^+^: 497.98696, found: 497.98602.

### 2-(3-(Difluoromethyl)-1-Methyl-1H-Pyrazole-4-Carboxamido)Phenyl 3,5-Difluorobenzenesulfonate (T18)

Gray powder, yield 86%. m.p. 143.1-144.0°C.^1^H NMR (400 MHz, CDCl_3_) *δ* 8.33–8.12 (m, 2H), 7.94 (s, 1H), 7.45–7.22 (m, 3H), 7.11–7.03 (m, 3H),7.00 (t, J = 54.0 Hz, 1H), 3.98 (s, 3H).^13^C NMR (101 MHz, CDCl_3_) *δ* 164.11, 164.00, 161.57, 161.45, 159.20, 144.23, 143.95, 143.68, 139.45, 137.91, 137.82, 137.73, 134.57, 130.68, 128.36, 125.15, 123.86, 122.38, 116.42, 113.44, 112.50, 112.41, 112.30, 112.21, 111.11, 110.83, 110.58, 110.33(t, J = 235.4 Hz), 39.84. HRMS (ESI): calculated for C_18_H_13_F_4_N_3_O_4_S [M + Na]^+^:466.04606, found: 466.04663.

### 2-(3-(Difluoromethyl)-1-Methyl-1H-Pyrazole-4-Carboxamido)Phenyl 2,5-Difluorobenzenesulfonate (T19)

Light yellow powder, yield 80%. m.p. 141.4-141.6°C.^1^H NMR (400 MHz, CDCl_3_) *δ* 8.33 (s, 1H), 8.29 (dd, J = 8.2, 1.6 Hz, 1H), 7.95 (s, 1H), 7.58 (ddd, J = 7.0, 5.2, 3.2 Hz, 1H), 7.43–7.32 (m, 1H), 7.33–7.27 (m, 1H), 7.25–7.20 (m, 1H), 7.18 (dd, J = 8.1, 1.7 Hz, 2H), 7.09 (t, J = 54.1 Hz, 1H), 7.08 (dd, J = 15.6, 1.6 Hz, 2H), 3.99 (s, 3H).^13^C NMR (101 MHz, CDCl_3_) *δ* 159.50, 159.06, 156.94, 156.55, 154.39, 145.44, 145.18, 144.92, 138.78, 133.15, 130.87, 128.43, 125.06, 124.42, 124.34, 124.27, 124.19, 124.10, 124.04, 123.55, 122.51, 119.42, 119.34, 119.18, 119.10, 118.33, 118.06, 116.57, 112.73, 110.39, 108.05, 39.91. HRMS (ESI): calculated for C_18_H_13_F_4_N_3_O_4_S [M + Na]^+^: 466.04606, found: 466.04663.

### 2-(3-(Difluoromethyl)-1-Methyl-1H-Pyrazole-4-Carboxamido)Phenyl 2,4-Difluorobenzenesulfonate (T20)

light yellow powder, yield 79%. m.p. 148.7-149.6°C.^1^H NMR (400 MHz, CDCl_3_) *δ* 8.36 (s, 1H), 8.30 (dd, J = 8.3, 1.6 Hz, 1H), 7.95 (s, 1H), 7.90 (ddd, J = 8.9, 7.8, 5.9 Hz, 1H), 7.30 (ddd, J = 8.5, 7.4, 1.6 Hz, 1H), 7.15 (dd, J = 8.3, 1.6 Hz, 1H), 7.10–7.05 (m, 1H),7.08 (t, J = 54.1 Hz, 1H), 7.04–6.95 (m, 2H), 4.00 (s, 3H).^13^C NMR (101 MHz, CDCl_3_) *δ* 159.46, 145.53, 138.87, 135.14, 133.40, 133.01, 132.81, 132.69, 130.97, 129.09, 129.00, 128.94, 128.76, 128.36, 124.93, 123.41, 123.01, 121.07, 116.49, 112.41, 110.07, 107.73, 39.90. HRMS (ESI): calculated for C_18_H_13_F_4_N_3_O_4_S [M + Na]^+^: 466.04606, found: 466.04663.

### 2-(3-(Difluoromethyl)-1-Methyl-1H-Pyrazole-4-Carboxamido)Phenyl 2-(Trifluoromethyl)Benzenesulfonate (T21)

Gray powder, yield 69%. m.p. 120.6-121.2°C.^1^H NMR (400 MHz, CDCl_3_) *δ* 8.36 (s, 1H), 8.30 (dd, J = 8.3, 1.6 Hz, 1H), 7.95 (s, 1H), 7.90 (ddd, J = 8.9, 7.8, 5.9 Hz, 1H), 7.30 (ddd, J = 8.5, 7.4, 1.6 Hz, 1H), 7.15 (dd, J = 8.3, 1.6 Hz, 1H), 7.10–7.05 (m, 2H),7.09 (t, J = 54.1 Hz, 1H), 7.04–6.95 (m, 2H), 4.00 (s, 3H).^13^C NMR (101 MHz, CDCl_3_) *δ* 168.62, 166.13, 166.02, 162.04, 161.91, 159.48, 159.31, 145.37, 145.11, 144.85, 138.85, 133.57, 133.46, 133.26, 130.97, 128.36, 125.00, 123.51, 122.57, 119.62, 119.49, 116.67, 112.78, 112.75, 112.56, 112.53, 110.44, 108.10, 106.65, 106.41, 106.39, 106.15, 39.92.HRMS (ESI): calculated for C_19_H_14_F_5_N_3_O_4_S [M + Na]^+^: 498.05229, found: 498.05078.

### 2-(3-(Difluoromethyl)-1-Methyl-1H-Pyrazole-3-Carboxamido)Phenyl 3-(Trifluoromethyl)Benzenesulfonate (T22)

Light yellow powder, yield 73%. m.p. 147.2-148.3°C.^1^H NMR (400 MHz, CDCl_3_) *δ* 8.27–8.15 (m, 2H), 8.12 (s, 1H), 7.92 (d, J = 7.8 Hz, 1H), 7.91 (s, 1H), 7.87 (d, J = 7.9 Hz, 1H), 7.62 (t, J = 7.9 Hz, 1H), 7.29 (ddd, J = 8.6, 5.8, 3.1 Hz, 1H), 7.13–7.03 (m, 2H), 6.96 (t, J = 54.1 Hz, 1H), 3.97 (s, 3H).^13^C NMR (101 MHz, CDCl_3_) *δ* 159.09, 144.17, 143.89, 143.62, 139.32, 136.04, 134.52, 132.64, 132.30, 131.96, 131.82, 131.63, 131.46, 131.43, 131.39, 131.36, 130.75, 130.30, 128.28, 125.69, 125.65, 125.62, 125.58, 125.02, 124.22, 123.59, 122.64, 121.50, 116.36, 113.43, 111.11, 108.78, 39.79.HRMS (ESI): calculated for C_19_H_14_F_5_N_3_O_4_S [M + Na]^+^: 498.05229, found: 498.05078.

### 2-(3-(Difluoromethyl)-1-Methyl-1H-Pyrazole-4-Carboxamido)Phenyl 4-Methoxybenzenesulfonate (T23)

Gray powder, yield 83%. m.p. 130.0-131.1°C.^1^H NMR (400 MHz, CDCl_3_) *δ* 8.35 (s, 1H), 8.28 (dd, J = 8.2, 1.6 Hz, 1H), 7.91 (s, 1H), 7.78–7.70 (m, 2H), 7.30–7.23 (m, 1H), 7.09 (t, J = 54.0 Hz, 1H), 7.01 (td, J = 7.8, 1.6 Hz, 1H), 6.93-6.86 (m, 3H), 4.00 (s, 3H), 3.86 (s, 3H).^13^C NMR (101 MHz, CDCl_3_) *δ* 164.70, 159.36, 144.97(t, J = 25.8 Hz), 139.56, 133.27, 131.15, 131.02(×2), 127.90, 125.68, 124.72, 123.18, 122.93, 116.83, 114.64(×2), 110.48(t, J = 235.4 Hz), 55.93, 39.90.HRMS (ESI): calculated for C_19_H_17_F_2_N_3_O_5_S [M + Na]^+^:460.07547, found: 460.07503.

### 2-(3-(Difluoromethyl)-1-Methyl-1H-Pyrazole-4-Carboxamido)Phenyl Phenylmethanesulfonate (T24)

Light yellow powder, yield 83%. m.p. 123.4-124.2°C.^1^H NMR (400 MHz, CDCl_3_) *δ* 8.35 (dd, J = 8.3, 1.6 Hz, 1H), 8.27 (s, 1H), 7.68 (s, 1H), 7.51–7.44 (m, 2H), 7.40 (dd, J = 5.0, 2.0 Hz, 3H), 7.31 (td, J = 7.9, 1.5 Hz, 1H), 7.20 (m, 1H),7.15 (t, J = 54.0 Hz, 1H), 7.09 (td, J = 7.8, 1.6 Hz, 1H), 7.02–6.97 (m, 1H), 4.65 (s, 2H), 3.97 (s, 3H).^13^C NMR (101 MHz, CDCl3) *δ* 159.54, 145.26(t, J = 25.8 Hz),138.25-132.95, 131.24, 131.09(×2), 129.67, 129.24, 128.23(×2), 126.88, 125.03, 123.19, 122.90,115.83,110.39 (t, J = 235.4 Hz), 57.29, 39.92. HRMS (ESI): calculated for C_19_H_17_F_2_N_3_O_4_S [M + Na]^+^:444.08055, found: 448.07975.

### 2-(3-(Difluoromethyl)-1-Methyl-1H-Pyrazole-4-Carboxamido)Phenyl Naphthalene-2-Sulfonate (T25)

Light yellow powder, yield 80%. m.p. 158.7-159.5°C.^1^H NMR (400 MHz, CDCl_3_) *δ* 8.43 (d, J = 1.9 Hz, 1H), 8.30–8.17 (m, 2H), 7.94–7.81 (m, 3H), 7.71 (ddd, J = 13.8, 8.5, 1.6 Hz, 2H), 7.65–7.58 (m, 2H), 7.31–7.21 (m, 1H), 7.04–6.99 (m, 2H), 6.98 (t, J = 54.1 Hz, 1H), 3.90 (s, 3H).^13^C NMR (101 MHz, CDCl_3_) *δ* 159.11, 144.39(t, J = 25.7 Hz), 139.66, 135.70, 133.52, 131.91, 131.81, 130.94, 130.72, 129.89, 129.82, 129.55, 128.15, 128.01, 128.00, 124.87, 123.32, 123.04, 122.76, 116.48, 110.66 (t, J = 234.8 Hz, 1H), 39.76. HRMS (ESI): calculated for C_22_H_17_F_2_N_3_O_4_S [M + Na]^+^:480.08055, found: 448.08005.

### 2-(3-(Difluoromethyl)-1-Methyl-1H-Pyrazole-4-Carboxamido)Phenyl 2,4,6-Trimethylbenzenesulfonate (T26)

White powder, yield 81%. m.p. 155.0-155.4°C.^1^H NMR (400 MHz, CDCl_3_) *δ* 8.56 (s, 1H), 8.35 (dd, J = 8.3, 1.6 Hz, 1H), 7.95 (s, 1H), 7.28–7.23 (m, 1H), 7.15 (t, J = 54.1 Hz, 1H), 7.01 (s, 2H), 6.91 (td, J = 7.8, 1.6 Hz, 1H), 6.52 (dd, J = 8.2, 1.5 Hz, 1H), 4.01 (s, 3H), 2.55 (s, 6H), 2.35 (s, 3H).^13^C NMR (101 MHz, CDCl_3_) *δ* 159.50, 145.40 (t, J = 25.3 Hz), 144.77, 140.77(×2), 139.25, 132.64, 132.05(×2), 131.52, 129.59, 127.78, 124.57, 123.25, 121.97, 116.80, 110.09 (t, J = 234.8Hz, 1H), 39.85, 22.85(×2), 21.23.HRMS (ESI): calculated for C_21_H_21_F_2_N_3_O_4_S [M + Na]^+^:472.11185, found: 472.11150.

### 2-(3-(Difluoromethyl)-1-Methyl-1H-Pyrazole-4-Carboxamido)Phenyl 4-(Tert-butyl)Benzenesulfonate (T27)

Light yellow powder, yield 82%. m.p.149.7-150.5°C.^1^H NMR (400 MHz, CDCl_3_) *δ* 8.41 (s, 1H), 8.31 (dd, J = 8.3, 1.6 Hz, 1H), 7.94 (s, 1H), 7.81–7.73 (m, 2H), 7.56–7.46 (m, 2H), 7.26 (td, J = 7.8, 1.5 Hz, 1H),7.11 (t, J = 54.1 Hz, 1H), 7.00 (td, J = 7.9, 1.6 Hz, 1H), 6.89 (dd, J = 8.2, 1.5 Hz, 1H), 4.00 (s, 3H), 1.32 (s, 9H).^13^C NMR (101 MHz, CDCl_3_) *δ* 159.33, 159.29, 145.04(t, J = 25.2 Hz), 139.43, 133.31, 131.48, 131.22, 128.57(×2), 127.93, 126.46(×2), 124.66, 123.12, 122.75, 116.81, 110.45 (t, J = 236.3 Hz), 39.91, 35.54, 31.05(×3). HRMS (ESI): calculated for C_22_H_23_F_2_N_3_O_4_S [M + Na]^+^: 486.12750, found: 486.12686.

### *In Vitro* Biological Evaluation

#### *In Vitro* Antifungal Assay

The test strains were Colletotrichum camelliae (C.camelliae), Pestalotiopsis theae (P. theae) provided by Guizhou Tea Research Institute, and Gibberella zeae (G. zeae), Rhizoctonia solani (R. solani) provided by Guizhou Institute of Plant Protection. In this study, the *in vitro* antifungal activity of the target compounds **T1-27** against four plant pathogens was screened by the mycelial growth rate method (Zhang et al., 2019). The tested compounds were dissolved in DMSO to prepare a 10 mg/ml stock solution before mixing with PDA. The PDA containing compounds at a concentration of 50 mg/L were then poured into sterilized Petri dishes for primary screening. Data Processing System (DPS, V9.50) was used for statistical analysis of test data, and Duncan’s new multiple range method was used to test the significance of differences. The EC_50_ values and 95% confidence limits were calculated after testing the inhibition rates, based on the above method. The inhibition rate of the potent compounds was further tested and the corresponding EC_50_ values were calculated by using DPS. This test method is provided in the Supporting information.

#### *In Vivo* Antiviral Activities Assay

The *in vivo* antiviral activities of target compounds **T1-27** against TMV were tested by the half leaf blight spot method previously reported in the literature(Chen et al., 2021; Xie et al., 2018). TMV was propagated in Nicotiana tabacum cv. K326 by the Gooding method. Antiviral activities of the target compounds against TMV *in vivo* were at 500 mg/L. The commercial antiviral agents Ningnanmycin and Chitosan oligosaccharides were severed as the positive controls. Data is processed in the same way as that of antifungal activity.

## Results and Discussion

### Chemistry

**SCHEME 2:**
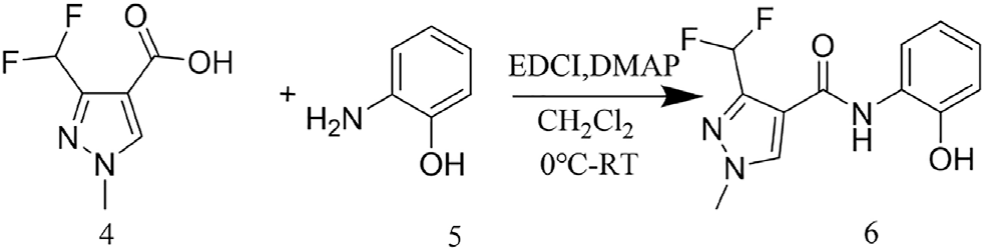
Synthesis of the key intermediate **6**.

**SCHEME 3:**
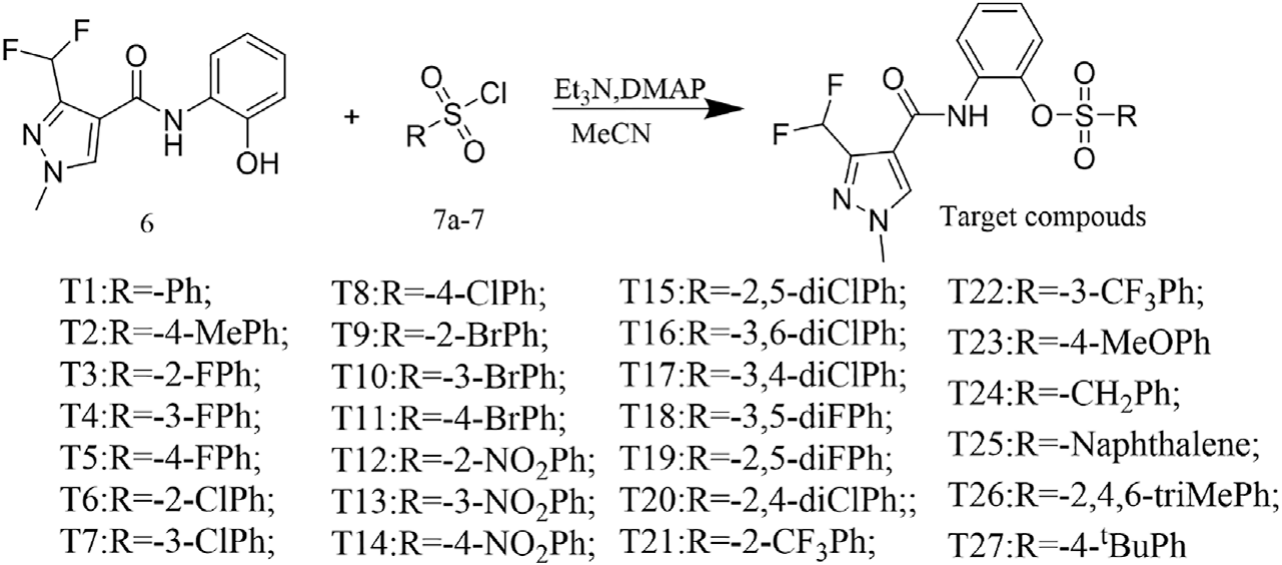
Synthesis of the target compounds.

**FIGURE 1:**
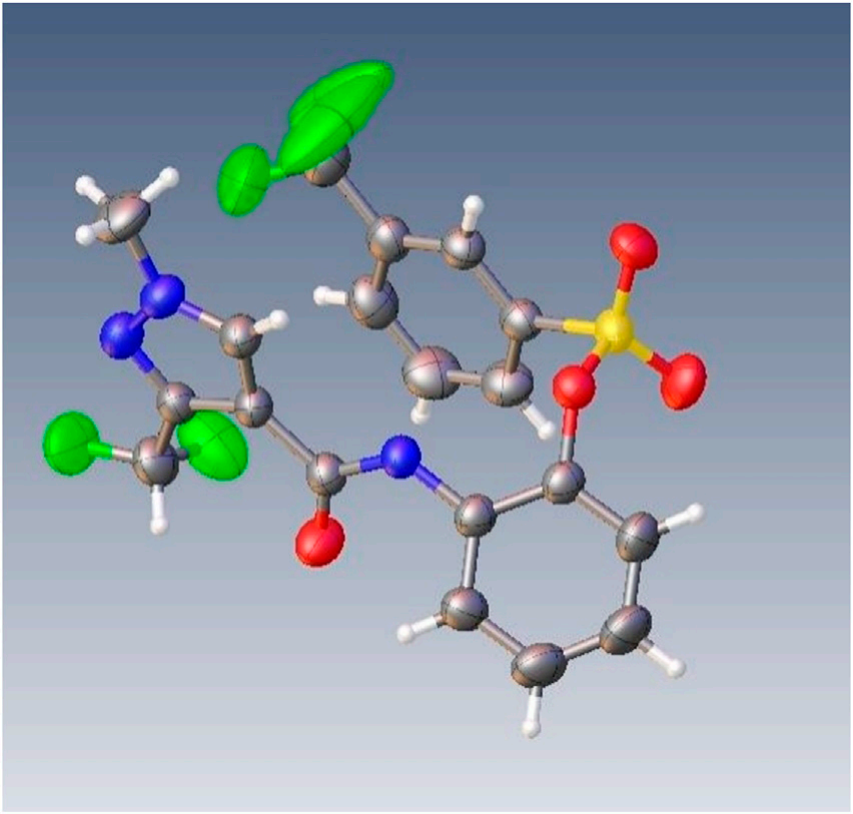
The single-crystal X-ray diffraction of compound T22.

The reaction between the starting material, ethyl 4,4-difluoro-3-oxobutanoate **1)** and triethyl orthoformate in acetic anhydride at 140°C, yielded ethyl 2-(ethoxymethylene)-4,4-difluoro-3-oxobutanoate (compound **2**) (Sun and Zhou, 2015). Compound **2** was then treated with methylhydrazine to yield compound **3**, which was successively hydrolyzed with lithium hydroxide and hydrochloric acid to obtain a white solid of the key intermediate 3-(difluoromethyl)-1-methyl-1H-pyrazole-4-carboxylic acid (compound **4**) (Scheme 1). Thereafter, compound **6,** a light yellow solid, was formed by conjugating compound **4** with 2-aminophenol in CH_2_Cl_2_ using EDCI and DMAP (Scheme 2). Finally, different substituted moieties of arylsulfonyl chloride were reacted with compound **5** to yield the target compounds (Scheme 3). The structures of all key intermediates and target compounds were confirmed *via* ^1^H and ^13^C NMR and HRMS, and their spectra data are shown in the Supplementary Material. The single-crystal X-ray diffraction of compound **T22** showed that the compound is a sulfonate and not a sulfonamide. Figure 1 shows the crystal structure of **T22**, whose deposition number is CCDC 2168151.

### *In Vitro* Biological Evaluation

#### *In Vitro* Antifungal Assay

The preliminary *in vitro* antifungal activities of the 27 target compounds are presented in Tables 1, 2. Most of the target compounds exhibited some degree of antifungal activities against the four plant pathogens at 50 μg/ml (Table 1). Among the four plant pathogens, the target compounds, particularly **T24,** exhibited remarkable antifungal activity against R. solani. When R group was nitro group, the antifugal activity against R. solani was no more than 20%. It can be known from these data that the substituent on the benzene ring was a strong electron-withdrawing group, the antifungal activity was adversely affected. We also found that the activity of **T24** against R. solani was much higher than that of **T1** (Table 1). The only structural difference between these two compounds is the presence of an extra methylene group in **T24**, which is thought to enhance its antifungal activity. The compound **T24** (EC_50_ = 0.45 mg/L) was superior to the commercial fungicide hymexazol (EC_50_ = 10.49 mg/L), but closer to bixafen (EC_50_ = 0.25 mg/L) in its activity against R. solani (Table 2).

**TABLE 1:** Inhibition rate *in vitro* of target compounds **T1-27** at 50 μg/ml.

Compounds	Inhibition Rate (%)			
	R. Solani (36 h)	C.camelliae (120 h)	P. Theae (120 h)	G.Zeae (120 h)
T1	29.37 ± 1.02 k	20.30 ± 1.22 kl	30.30 ± 0.42 ij	30.20 ± 1.33 j
T2	51.59 ± 1.31 e	41.09 ± 1.10 de	31.60 ± 1.69 hi	42.50 ± 1.23 d
T3	30.95 ± 1.19 k	26.90 ± 1.09 i	33.75 ± 0.19 fg	26.88 ± 2.09 k
T4	10.14 ± 0.24 q	11.74 ± 0.26o	12.04 ± 1.04 r	18.21 ± 1.04 no
T5	13.23 ± 0.97 op	16.20 ± 0.27 n	18.23 ± 1.08 p	19.69 ± 0.97 n
T6	44.97 ± 0.92 g	34.17 ± 0.12 f	40.02 ± 0.42 d	30.67 ± 0.62 j
T7	62.96 ± 1.27 d	12.90 ± 1.16o	32.16 ± 0.17 gh	36.16 ± 1.36 h
T8	61.38 ± 1.39 d	30.18 ± 1.09 h	29.08 ± 0.19 jk	41.38 ± 2.49 de
T9	23.02 ± 1.06 m	21.02 ± 0.76 k	20.19 ± 0.46o	25.02 ± 1.16 kl
T10	30.56 ± 1.42 k	20.66 ± 1.02 kl	28.51 ± 0.32 k	25.69 ± 1.02 kl
T11	36.77 ± 1.21 i	30.07 ± 0.41 h	33.71 ± 0.42 fg	38.27 ± 1.41 fg
T12	12.43 ± 1.01 p	19.73 ± 0.70 kl	17.40 ± 0.80 p	16.43 ± 1.21o
T13	13.46 ± 1.09 op	19.40 ± 1.17 lm	12.66 ± 0.19 r	10.26 ± 1.49 p
T14	20.45 ± 0.91 n	23.25 ± 0.78 j	22.05 ± 0.88 mn	22.45 ± 0.71 m
T15	24.34 ± 1.08 m	21.06 ± 0.98 k	23.04 ± 0.13 lm	26.64 ± 1.00 k
T16	48.15 ± 1.26 f	28.05 ± 0.16 i	33.18 ± 0.19 fgh	43.19 ± 0.26 d
T17	34.13 ± 1.10 j	24.03 ± 1.01 j	29.03 ± 1.00 jk	33.03 ± 0.16 i
T18	81.48 ± 1.06 c	40.40 ± 1.78 de	35.98 ± 0.76 e	40.08 ± 0.96 ef
T19	45.74 ± 1.02 g	35.04 ± 1.12 f	34.74 ± 0.92 ef	38.87 ± 0.46 f
T20	44.18 ± 1.00 g	40.01 ± 0.90 e	24.18 ± 0.10 l	36.58 ± 0.90 gh
T21	14.81 ± 0.98o	17.80 ± 0.68 mn	14.81 ± 0.78 q	24.73 ± 0.88 kl
T22	27.25 ± 0.93 l	23.15 ± 0.63 j	17.25 ± 0.13 p	26.35 ± 0.73 kl
T23	20.11 ± 0.95 n	20.71 ± 0.36 kl	13.05 ± 0.65 r	24.41 ± 0.65 l
T24	**100.00 ± 0.00 a**	45.31 ± 0.47 c	62.40 ± 0.51 c	48.00 ± 1.10 c
T25	29.37 ± 0.40 k	31.07 ± 0.69 gh	20.30 ± 0.16o	39.07 ± 0.64 f
T26	30.69 ± 0.73 k	32.19 ± 0.33 g	21.30 ± 0.44 no	32.64 ± 0.91 i
T27	40.21 ± 0.98 h	42.12 ± 1.84 d	20.20 ± 0.61°	26.26 ± 0.68 kl
hymexazol	84.28 ± 0.96 b	54.91 ± 1.80 b	66.11 ± 3.20 b	67.33 ± 2.19 b
bixafen	**100.00 ± 0.00 a**	**79.49 ± 1.36 a**	**93.40 ± 1.77 a**	**100.00 ± 0.00 a**

Note: Data in the table are mean ± SD., Different lowercase letters in the same column indicate significant difference at *p* < 0.05 level by Duncan’s new multiple range test. The meaning of bold is only to emphasize the good activity of the two compounds.

**TABLE 2:** EC_50_ values of **T24** against R. solani.

Compound	Regression Equation	EC_50_ (mg/L)	*R* ^2^	95% confidence Interval (mg/L)
T24	y = 5.7941 + 1.3307x	0.45	0.9588	0.32-0.61
hymexazol	y = 3.9940 + 0.9853x	10.49	0.9949	6.35-17.33
bixafen	y = 5.7941 + 1.3307x	0.25	0.9976	0.13-0.47

#### *In Vivo* Antiviral Activities of Compounds T1-27

The phenylsulfonyl fragment has been reported to increase the antiviral activity (Hadházi et al., 2017), we synthesised novel sulfonate scaffold-containing pyrazolecarbamide and evaluated their antiviral activities.The curative, protective, and inactivation effects of the 27 target compounds against TMV were evaluated using the half leaf blight spot method (Liu et al., 2021; Zhang et al., 2021), and the commercial agents, Ningnanmycin and Chitosan oligosaccharide, served as positive controls. Compound **T18** (54.2%) exhibited a close curative activity to ningnanmycin (55.3%) at 500 mg/ml. Additionally, most of the target compounds exhibited protective effects *in vivo*, and the protective effects of compounds **T5** (50.4%) and **T12** (50.2%) were similar to that of Ningnanmycin (50.7%). Although the target compounds had lower inactivation effects than ningnanmycin, most of them exhibited better inactivation activities than Chitosan oligosaccharides (Table 3).

**TABLE 3:** Antiviral activity of the target compounds against TMV *in vivo* (500 mg/L).

Compound	Curative effect(%)	Protective effect(%)	Inactivation effect(%)
T1	30.9 ± 2.4 fg	40.1 ± 2.2 ghi	54.6 ± 3.2 jkl
T2	35.2 ± 1.6 f	43.1 ± 1.4 defghi	53.2 ± 1.3 jkl
T3	32.8 ± 3.2 fg	49.8 ± 2.3 b	63.3 ± 2.3 efgh
T4	40.8 ± 2.9 e	43.8 ± 2.6 cdefgh	62.6 ± 4.2 efghi
T5	42.4 ± 4.5 de	50.4 ± 1.5 b	59.5 ± 1.7 ghijk
T6	42.5 ± 2.0 de	42.4 ± 2.4 efghi	57.6 ± 2.5 hijk
T7	43.8 ± 1.7 de	43.5 ± 1.4 defghi	56.5 ± 3.0 ijk
T8	45.9 ± 2.5 cde	47.2 ± 3.0 bcde	62.8 ± 2.2 efghi
T9	35.2 ± 2.7 f	41.1 ± 3.2 ghi	50.5 ± 3.9 lmn
T10	32.2 ± 2.3 fg	42.5 ± 2.4 defghi	55.1 ± 3.4 hijk
T11	33.8 ± 4.0 f	49.8 ± 1.9 b	57.3 ± 3.5 jkl
T12	41.9 ± 2.0 de	50.2 ± 3.6 b	60.6 ± 2.4 fghij
T13	43.0 ± 3.7 de	45.4 ± 3.5 bcdefg	58.5 ± 4.7 hijk
T14	44.5 ± 3.1 de	43.0 ± 3.9 defghi	49.6 ± 4.5 lmn
T15	40.8 ± 0.7 e	49.5 ± 4.4 b	46.5 ± 3.7 no
T16	42.9 ± 3.1 de	48.0 ± 3.0 bcd	72.8 ± 4.9 bc
T17	41.5 ± 3.7 e	41.1 ± 4.2 fghi	67.6 ± 4.3 de
T18	54.2 ± 3.6 ab	49.1 ± 4.4 bc	70.2 ± 4.6 bcd
T19	46.9 ± 3.4 cd	40.1 ± 3.2 ghi	74.6 ± 4.2 b
T20	49.8 ± 3.9 bc	45.8 ± 4.6 bcdef	68.6 ± 3.9 cde
T21	28.4 ± 2.9 g	40.3 ± 1.5 fghi	65.3 ± 2.1 defg
T22	41.2 ± 2.0 e	32.4 ± 1.8 j	58.7 ± 3.8 hijk
T23	33.8 ± 1.7 f	38.6 ± 2.6 hi	66.3 ± 3.9 def
T24	35.9 ± 2.5 f	37.9 ± 3.1 i	42.8 ± 3.7o
T25	33.8 ± 1.7 f	45.6 ± 1.7 bcdefg	57.5 ± 1.9 hijk
T26	45.9 ± 2.5 cde	43.2 ± 2.8 defghi	49.8 ± 2.9 lmn
T27	30.9 ± 1.7 fg	40.2 ± 2.9 fghi	57.8 ± 2.1 hijk
Chitosan oligosaccharides	54.6 ± 2.7 a	57.6 ± 2.2 a	47.9 ± 1.5 mno
Ningnanmycin	55.3 ± 1.2 a	50.7 ± 1.1 b	98.1 ± 1.0 a

Note: Data in the table are mean ± SD., Different lowercase letters in the same column indicate significant difference at *p* < 0.05 level by Duncan’s new multiple range test.

## Conclusion

In summary, 27 novel pyrazolecarbamide derivatives bearing a sulfonate fragment were synthesized and screened for their *in vitro* antifungal and *in vivo* antiviral activities against four plant pathogens (C. camelliae, P, theae, G. zeae, and R. solani). The structures of these compounds were identified using the single-crystal X-ray diffraction and spectral data obtained *via* ^1^H and ^13^C NMR and HRMS spectroscopy. The preliminary bioassay results showed that the target compounds exhibited certain inhibitory activities against the test fungi and TMV. Compound **T24** exhibited excellent antifungal activities against R. solani compared to the commercial fungicide hymexazol, almost similar to bixafen. Moreover, the target compounds displayed protective effects *in vivo* against TMV. Thus, our research group is conducting further structural optimization of the target compounds for wide-scale field application.

## Data Availability

The original contributions presented in the study are included in the article/Supplementary Material, further inquiries can be directed to the corresponding author.

## References

[B1] Chen M. Su S. Zhou Q. Tang X. Liu T. Peng F. (2021). Antibacterial and Antiviral Activities and Action Mechanism of Flavonoid Derivatives with a Benzimidazole Moiety. J. Saudi Chem. Soc. 25, 101194. 10.1016/j.jscs.2020.101194

[B2] El Shehry M. F. Ghorab M. M. Abbas S. Y. Fayed E. A. Shedid S. A. Ammar Y. A. (2018). Quinoline Derivatives Bearing Pyrazole Moiety: Synthesis and Biological Evaluation as Possible Antibacterial and Antifungal Agents. Eur. J. Med. Chem. 143, 1463–1473. 10.1016/j.ejmech.2017.10.046 29113746

[B3] Fisher M. C. Henk D. A. Briggs C. J. Brownstein J. S. Madoff L. C. McCraw S. L. (2012). Emerging Fungal Threats to Animal, Plant and Ecosystem Health. Nature 484, 186–194. 10.1038/nature10947 22498624 PMC3821985

[B4] Hadházi Á. Pascolutti M. Bailly B. Dyason J. C. Borbás A. Thomson R. J. (2017). A Sialosyl Sulfonate as a Potent Inhibitor of Influenza Virus Replication. Org. Biomol. Chem. 15, 5249–5253. 10.1039/C7OB00947J 28540971

[B5] Huang T.-J. Chuang H. Liang Y.-C. Lin H.-H. Horng J.-C. Kuo Y.-C. (2015). Design, Synthesis, and Bioevaluation of Paeonol Derivatives as Potential Anti-HBV Agents. Eur. J. Med. Chem. 90, 428–435. 10.1016/j.ejmech.2014.11.050 25461891

[B6] Jiang X. Wei X. Lin F. Zhang Z. Yao G. Yang S. (2020). Substrate-Controlled [5+1] Annulation of 5-Amino-1H -phenylpyrazoles with Alkenes: Divergent Synthesis of Multisubstituted 4,5-Dihydropyrazolo[1,5-A ]quinazolines. Eur. J. Org. Chem. 2020, 3997–4003. 10.1002/ejoc.202000536

[B7] Kang G.-Q. Duan W.-G. Lin G.-S. Yu Y.-P. Wang X.-Y. Lu S.-Z. (2019). Synthesis of Bioactive Compounds from 3-Carene (II): Synthesis, Antifungal Activity and 3D-QSAR Study of (Z)- and (E)-3-Caren-5-One Oxime Sulfonates. Molecules 24 (3), 477. 10.3390/molecules24030477 PMC6384770 30699975

[B8] Kanungo M. Joshi J. (2014). Impact of Pyraclostrobin (F-500) on Crop Plants. Plant Sci. Today. 1, 174–178. 10.14719/pst.2014.1.3.60

[B9] Kasiotis K. M. Tzanetou E. N. Haroutounian S. A. (2014). Pyrazoles as Potential Anti-angiogenesis Agents: A Contemporary Overview. Front. Chem. 2, 78. 10.3389/fchem.2014.00078 25250310 PMC4158875

[B10] Liu T. Peng F. Cao X. Liu F. Wang Q. Liu L. (2021). Design, Synthesis, Antibacterial Activity, Antiviral Activity, and Mechanism of Myricetin Derivatives Containing a Quinazolinone Moiety. ACS Omega 6, 30826–30833. 10.1021/acsomega.1c05256 34805711 PMC8600648

[B11] Mu J.-X. Shi Y.-X. Yang M.-Y. Sun Z.-H. Liu X.-H. Li B.-J. (2016). Design, Synthesis, DFT Study and Antifungal Activity of Pyrazolecarboxamide Derivatives. Molecules 21 (1), 68. 10.3390/molecules21010068 26760990 PMC6274113

[B12] Saleh N. M. El-Gazzar M. G. Aly H. M. Othman R. A. (2020). Novel Anticancer Fused Pyrazole Derivatives as EGFR and VEGFR-2 Dual TK Inhibitors. Front. Chem. 7, 917. 10.3389/fchem.2019.00917 32039146 PMC6993756

[B13] Si W.-J. Wang X.-B. Chen M. Wang M.-Q. Lu A.-M. Yang C.-L. (2019). Design, Synthesis, Antifungal Activity and 3D-QSAR Study of Novel Pyrazole Carboxamide and Niacinamide Derivatives Containing Benzimidazole Moiety. New J. Chem. 43, 3000–3010. 10.1039/C8NJ05150J

[B14] Su S. Zhou Q. Tang X. Peng F. Liu T. Liu L. (2021). Design, Synthesis, and Antibacterial Activity of Novel Myricetin Derivatives Containing Sulfonate. Monatsh. Chem. 152, 345–356. 10.1007/s00706-021-02739-1

[B15] Sun J. Zhou Y. (2015). Synthesis and Antifungal Activity of the Derivatives of Novel Pyrazole Carboxamide and Isoxazolol Pyrazole Carboxylate. Molecules 20, 4383–4394. 10.3390/molecules20034383 25759955 PMC6272414

[B16] Sun R. Wang Z. Li Y. Xiong L. Liu Y. Wang Q. (2013). Design, Synthesis, and Insecticidal Evaluation of New Benzoylureas Containing Amide and Sulfonate Groups Based on the Sulfonylurea Receptor Protein Binding Site for Diflubenzuron and Glibenclamide. J. Agric. Food Chem. 61, 517–522. 10.1021/jf304468b 23305601

[B17] Wang R. Zhi X. Li J. Xu H. (2015). Synthesis of Novel Oxime Sulfonate Derivatives of 2′(2′,6′)-(Di)chloropicropodophyllotoxins as Insecticidal Agents. J. Agric. Food Chem. 63, 6668–6674. 10.1021/acs.jafc.5b02036 26166302

[B18] Wang X. Wang A. Qiu L. Chen M. Lu A. Li G. (2020). Expedient Discovery for Novel Antifungal Leads Targeting Succinate Dehydrogenase: Pyrazole-4-Formylhydrazide Derivatives Bearing a Diphenyl Ether Fragment. J. Agric. Food Chem. 68, 14426–14437. 10.1021/acs.jafc.0c03736 33216530

[B19] Wang X. Wang X. Zhou B. Long J. Li P. (2021). Design, Synthesis, and Evaluation of New 4( 3 H )‐quinazolinone Derivatives Containing a Pyrazole Carboxamide Moiety. J. Heterocycl. Chem. 58, 2109–2116. 10.1002/jhet.4334

[B20] Wei C. Zhao L. Sun Z. Hu D. Song B. (2020). Discovery of Novel Indole Derivatives Containing Dithioacetal as Potential Antiviral Agents for Plants. Pestic. Biochem. Physiol. 166, 104568. 10.1016/j.pestbp.2020.104568 32448422

[B21] Wu J. Song B.-A. Hu D.-Y. Yue M. Yang S. (2012). Design, Synthesis and Insecticidal Activities of Novel Pyrazole Amides Containing Hydrazone Substructures. Pest. Manag. Sci. 68 (5), 801–810. 10.1002/ps.2329 22190278

[B22] Xie D. Shi J. Zhang A. Lei Z. Zu G. Fu Y. (2018). Syntheses, Antiviral Activities and Induced Resistance Mechanisms of Novel Quinazoline Derivatives Containing a Dithioacetal Moiety. Bioorg. Chem. 80, 433–443. 10.1016/j.bioorg.2018.06.026 29986188

[B23] Yan Z. Liu A. Huang M. Liu M. Pei H. Huang L. (2018). Design, Synthesis, DFT Study and Antifungal Activity of the Derivatives of Pyrazolecarboxamide Containing Thiazole or Oxazole Ring. Eur. J. Med. Chem. 149, 170–181. 10.1016/j.ejmech.2018.02.036 29501939

[B24] Zeng X.-W. Huang N. Xu H. Yang W.-B. Yang L.-M. Qu H. (2010). Anti Human Immunodeficiency Virus Type 1 (HIV-1) Agents 4. Discovery of 5,5'-(p-Phenylenebisazo)-8-Hydroxyquinoline Sulfonates as New HIV-1 Inhibitors *In Vitro* . Chem. Pharm. Bull. 58, 976–979. 10.1248/cpb.58.976 20606350

[B25] Zhang A. Yue Y. Yang J. Shi J. Tao K. Jin H. (2019). Design, Synthesis, and Antifungal Activities of Novel Aromatic Carboxamides Containing a Diphenylamine Scaffold. J. Agric. Food Chem. 67, 5008–5016. 10.1021/acs.jafc.9b00151 30977370

[B26] Zhang J. He F. Chen J. Wang Y. Yang Y. Hu D. (2021). Purine Nucleoside Derivatives Containing a Sulfa Ethylamine Moiety: Design, Synthesis, Antiviral Activity, and Mechanism. J. Agric. Food Chem. 69, 5575–5582. 10.1021/acs.jafc.0c06612 33988985

[B27] Zhou Q. Tang X. Chen S. Zhan W. Hu D. Zhou R. (2022). Design, Synthesis, and Antifungal Activity of Novel Chalcone Derivatives Containing a Piperazine Fragment. J. Agric. Food Chem. 70, 1029–1036. 10.1021/acs.jafc.1c05933 35072471

