# Supplementary material for: Synthesis and Bioactivity of Novel Sulfonate Scaffold-Containing Pyrazolecarbamide Derivatives as Antifungal and Antiviral Agents
Source: Front Chem. 2022 Jun 22;10:928842. doi: 10.3389/fchem.2022.928842 (PMC9257181; doi:10.3389/fchem.2022.928842)
Supplement: Supplementary file 1 [file DataSheet1.PDF]

Synthesis and Bioactivity of Novel Sulfonate scaffold-containing Pyrazolecarbamide Derivatives as Antifungal and Antiviral Agents

Zhi-wei Lei[1,2]*, Jianmei Yao[2], Huifang Liu[2], Chiyu Ma[2],Wen Yang[2]

[1] *State Key Laboratory Breeding Base of Green Pesticide and Agricultural Bioengineering, Key Laboratory of Green Pesticide and Agricultural Bioengineering, Ministry of Education, Guizhou University, Huaxi District, Guiyang 550025, China*

[2] *Tea Research Institute, Guizhou Academy of Agricultural Sciences, Huaxi District,Guiyang 550006，China*

* Author to whom correspondence should be addressed; E-mail: leizhiwei816@163.com;

1. *In vitro* antifungal activity Test

The tested compounds were dissolved in DMSO to prepare a 10 mg/mL stock solution before mixing with PDA. The PDA containing compounds at a concentration of 50 mg/L were then poured into sterilized Petri dishes for primary screening. After 36-120 h at 28 °C, the colony diameter of each strain was measured. The commercial fungicides hymexazol and bixafen were severed as the positive controls. Each treatment was performed three times. The EC_{50} values were determined for the compounds with inhibition rate >85% at 50 mg/L. The EC_{50} values and 95% confidence limits were calculated after testing the inhibition rates, based on the above method. The inhibition rate of the potent compounds was further tested and the corresponding EC_{50} values were calculated by using Data Processing System (DPS, V 9.50).

$$\text{Inhibition rate\%} = (A-C)/(A-B) \times 100\ \%$$

A: colony diameter in control Petri dishes; B: diameter of mycelial disc; C: the colony diameter in the Petri dishes with tested compounds;

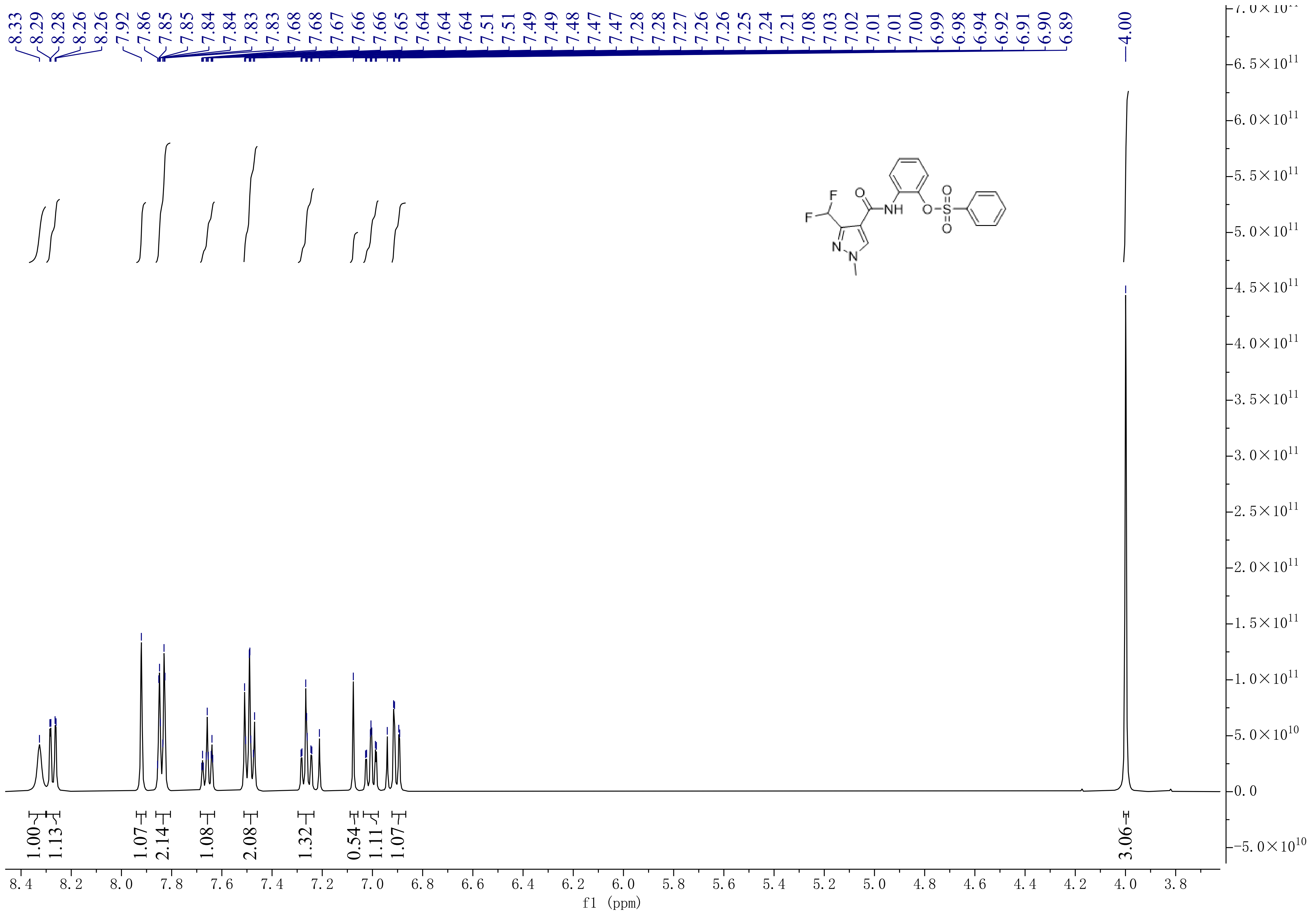

8.33 8.29 8.28 8.26 8.26 7.92 7.86 7.85 7.85 7.84 7.84 7.83 7.83 7.68 7.68 7.67 7.66 7.66 7.65 7.64 7.64 7.64 7.51 7.51 7.49 7.49 7.48 7.47 7.47 7.28 7.28 7.27 7.26 7.26 7.25 7.24 7.21 7.08 7.03 7.02 7.01 7.01 7.00 6.99 6.98 6.94 6.92 6.91 6.90 6.89
4.00
1.00 1.13 1.07 2.14 1.08 2.08 1.32 0.54 1.11 1.07 3.06
F F O NH O S O O N N
f1 (ppm)

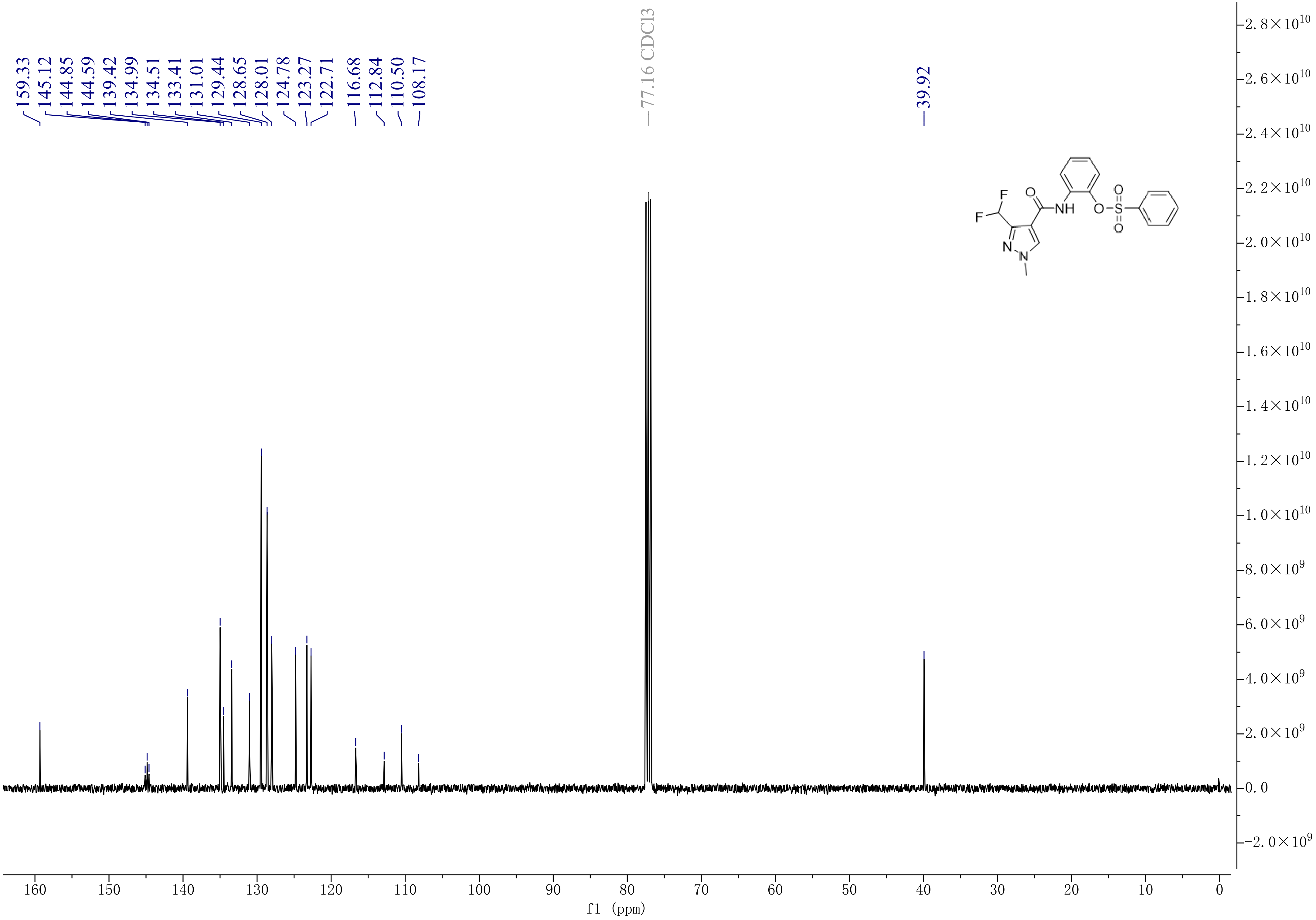

159.33
145.12
144.85
144.59
139.42
134.99
134.51
133.41
131.01
129.44
128.65
128.01
124.78
123.27
122.71
116.68
112.84
110.50
108.17
77.16 CDCl3
39.92
F
F
O
NH
O
S
O
O
N
N
160
150
140
130
120
110
100
90
80
70
60
50
40
30
20
10
0
f1 (ppm)
2.8×10^10
2.6×10^10
2.4×10^10
2.2×10^10
2.0×10^10
1.8×10^10
1.6×10^10
1.4×10^10
1.2×10^10
1.0×10^10
8.0×10^9
6.0×10^9
4.0×10^9
2.0×10^9
0.0
−2.0×10^9

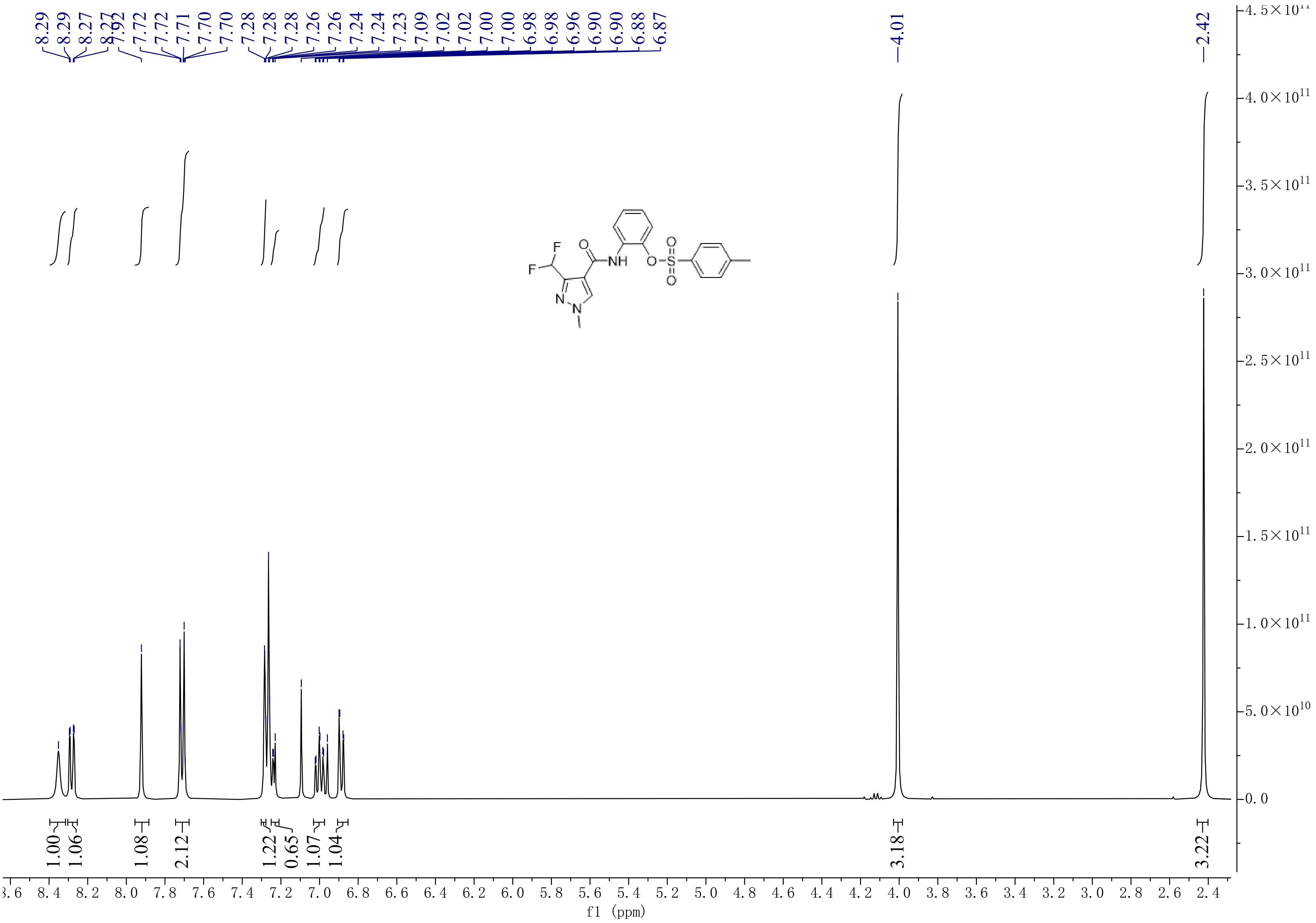
8.29
8.29
8.27
8.27
7.92
7.72
7.72
7.71
7.70
7.70
7.28
7.28
7.28
7.26
7.26
7.24
7.24
7.23
7.09
7.02
7.02
7.00
7.00
6.98
6.98
6.96
6.90
6.90
6.88
6.87
4.01
2.42
F
F
O
NH
O
S
O
O
N
N
1.00
1.06
1.08
2.12
1.22
0.65
1.07
1.04
3.18
3.22
4.5×10^11
4.0×10^11
3.5×10^11
3.0×10^11
2.5×10^11
2.0×10^11
1.5×10^11
1.0×10^11
5.0×10^10
0.0
f1 (ppm)

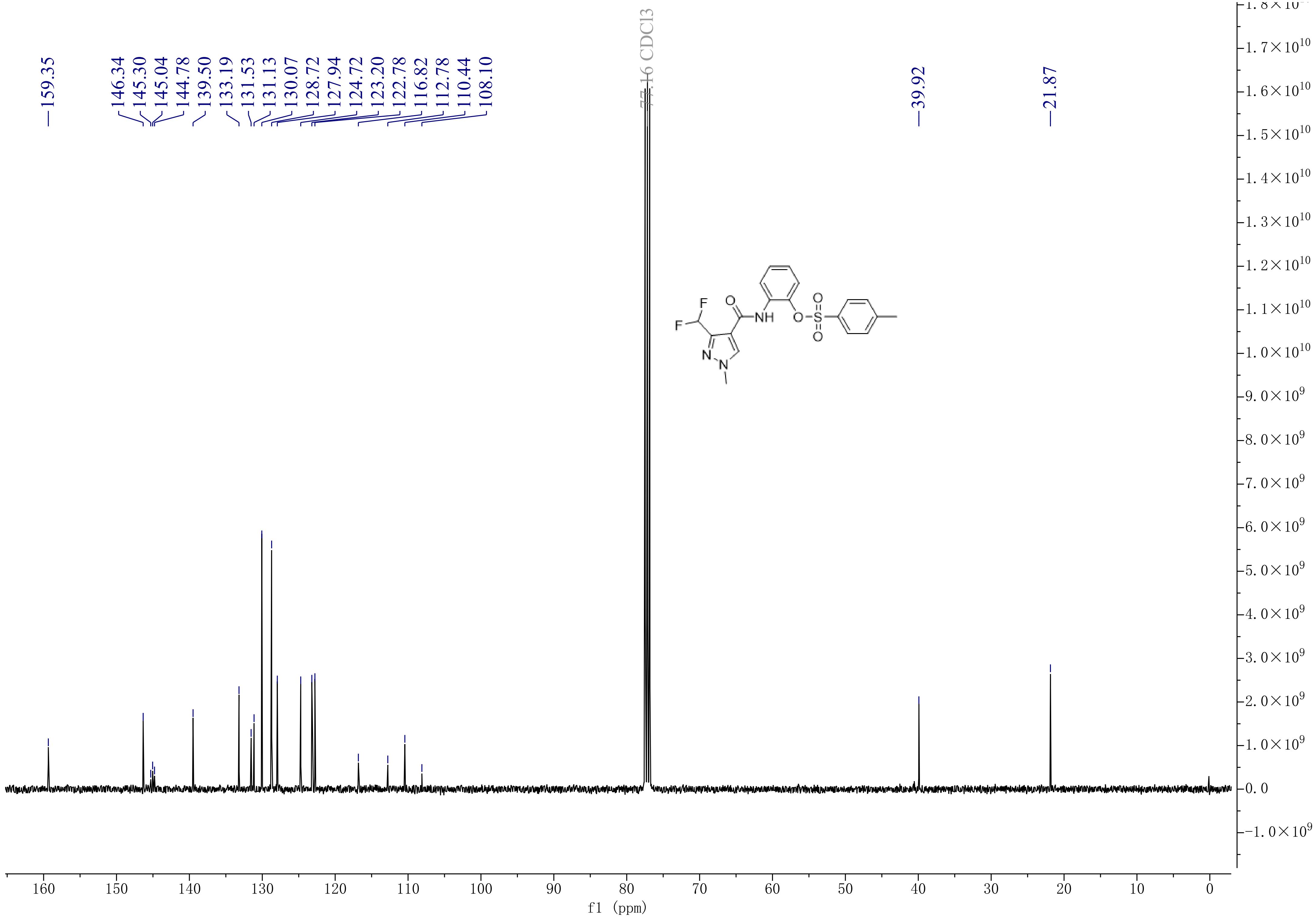
159.35
146.34
145.30
145.04
144.78
139.50
133.19
131.53
131.13
130.07
128.72
127.94
124.72
123.20
122.78
116.82
112.78
110.44
108.10
77.16 CDCl3
39.92
21.87
F
F
O
NH
O
S
O
O
N
N
f1 (ppm)
160
150
140
130
120
110
100
90
80
70
60
50
40
30
20
10
0
1.7×10^10
1.6×10^10
1.5×10^10
1.4×10^10
1.3×10^10
1.2×10^10
1.1×10^10
1.0×10^10
9.0×10^9
8.0×10^9
7.0×10^9
6.0×10^9
5.0×10^9
4.0×10^9
3.0×10^9
2.0×10^9
1.0×10^9
0.0
-1.0×10^9

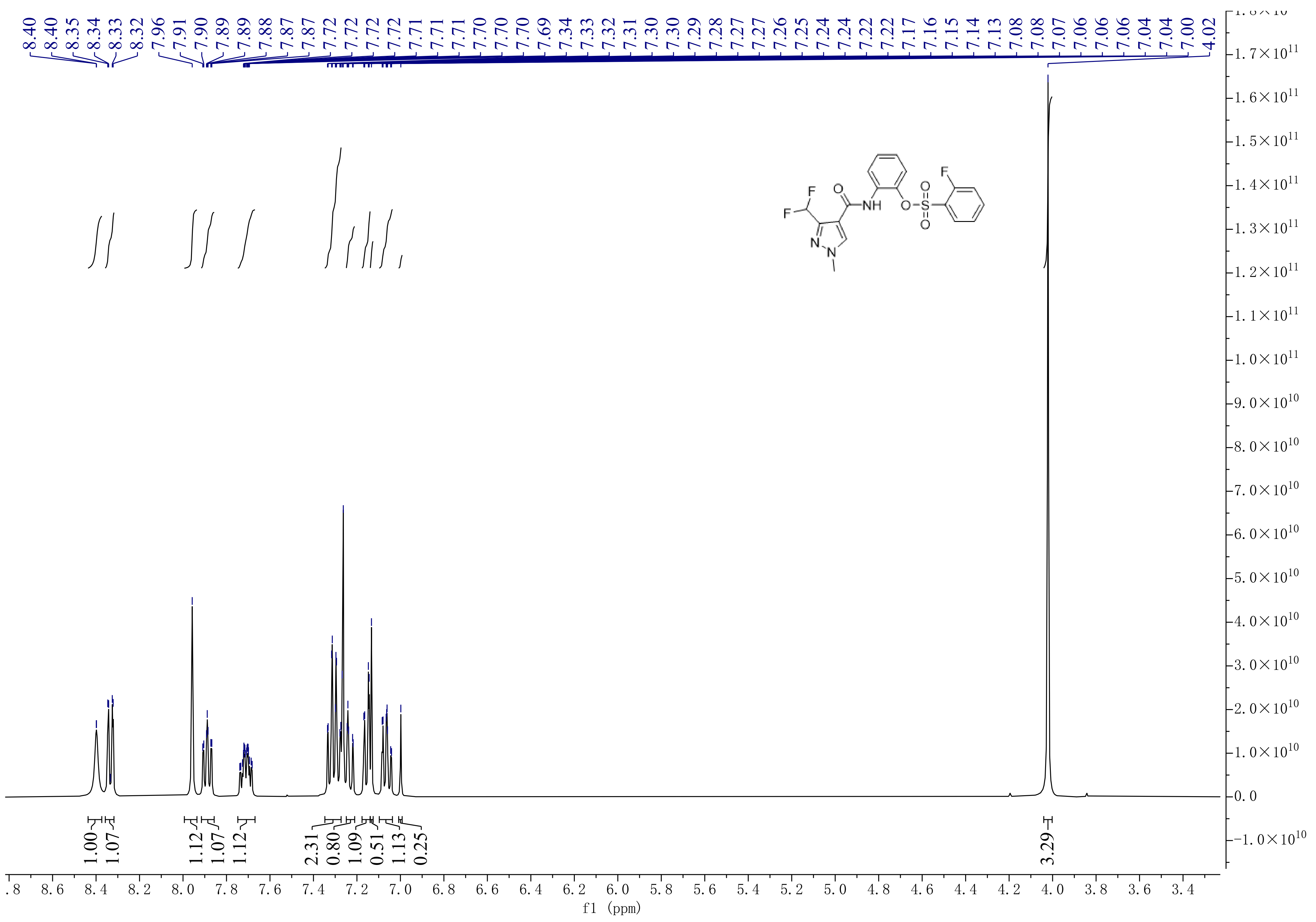
8.40
8.40
8.35
8.34
8.33
8.32
7.96
7.91
7.90
7.89
7.89
7.88
7.87
7.87
7.72
7.72
7.72
7.72
7.71
7.71
7.71
7.70
7.70
7.70
7.69
7.34
7.33
7.32
7.31
7.30
7.30
7.29
7.28
7.27
7.27
7.26
7.25
7.24
7.24
7.22
7.22
7.17
7.16
7.15
7.14
7.13
7.08
7.08
7.07
7.06
7.06
7.06
7.04
7.04
7.00
4.02
F
F
O
NH
N
N
F
O
O
S
O
1.00
1.07
1.12
1.07
1.12
2.31
0.80
1.09
0.51
1.13
0.25
3.29
f1 (ppm)

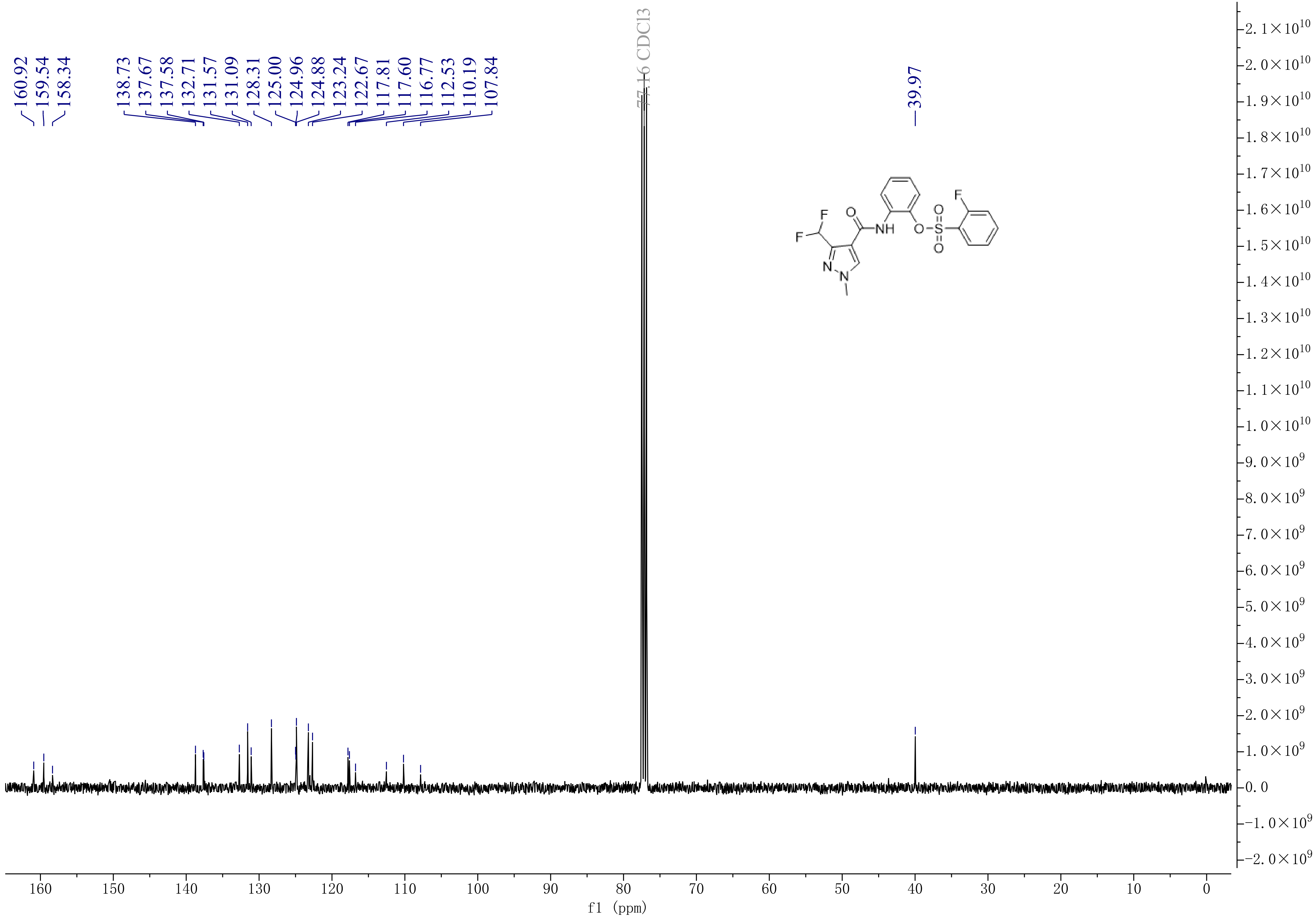
160.92
159.54
158.34
138.73
137.67
137.58
132.71
131.57
131.09
128.31
125.00
124.96
124.88
123.24
122.67
117.81
117.60
116.77
112.53
110.19
107.84
77.16 CDCl3
39.97
F
F
O
NH
O
S
O
O
F
N
N
160
150
140
130
120
110
100
90
80
70
60
50
40
30
20
10
0
f1 (ppm)

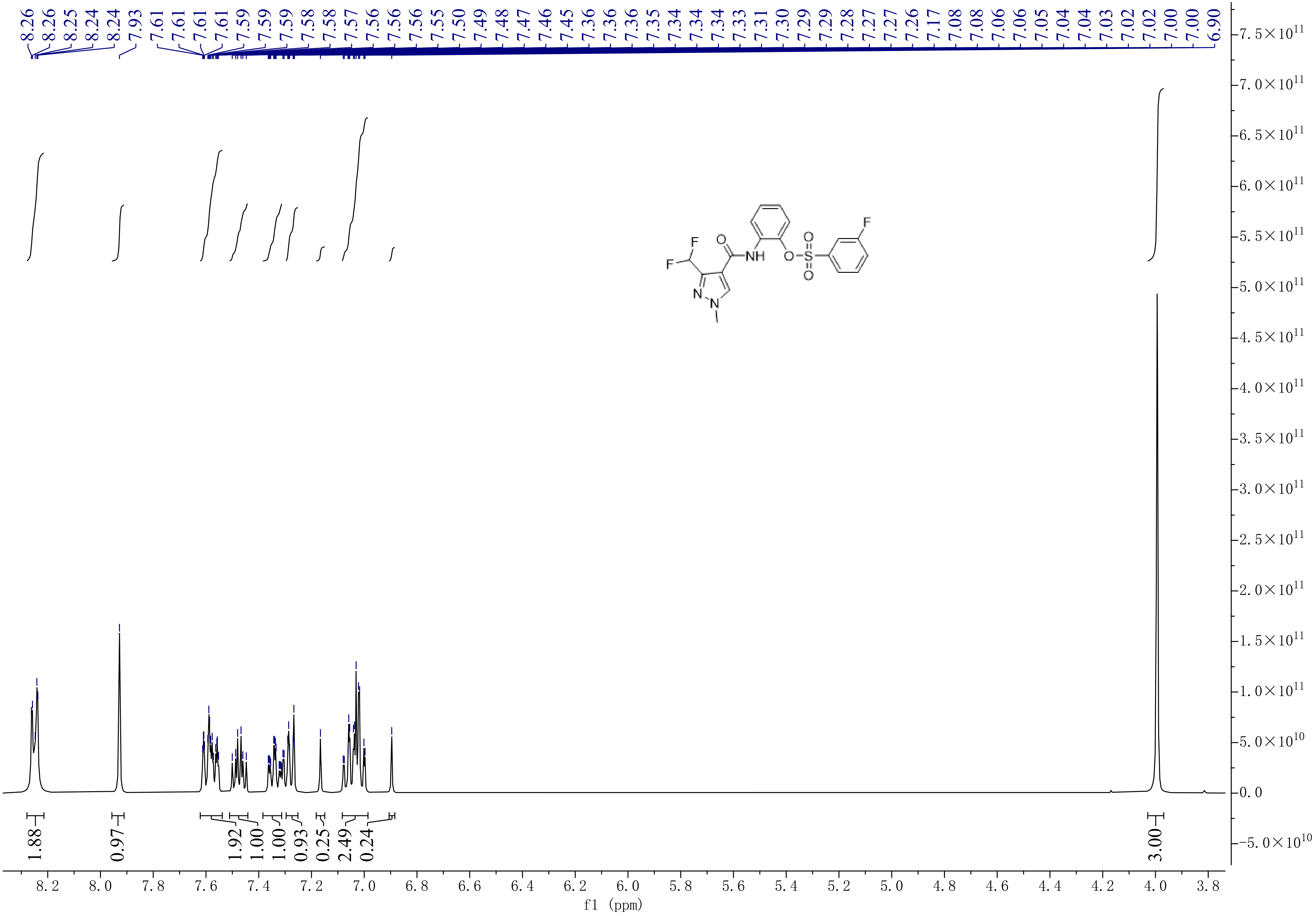
8.26 8.26 8.25 8.24 8.24 7.93 7.61 7.61 7.61 7.61 7.59 7.59 7.59 7.58 7.58 7.57 7.56 7.56 7.56 7.55 7.50 7.49 7.48 7.47 7.46 7.45 7.36 7.36 7.36 7.35 7.34 7.34 7.34 7.33 7.31 7.30 7.29 7.29 7.28 7.27 7.27 7.26 7.17 7.08 7.08 7.06 7.06 7.05 7.04 7.04 7.03 7.02 7.02 7.00 7.00 6.90
1.88
0.97
1.92
1.00
1.00
0.93
0.25
2.49
0.24
3.00
F
F
O
NH
O
S
O
O
F
N
N
f1 (ppm)

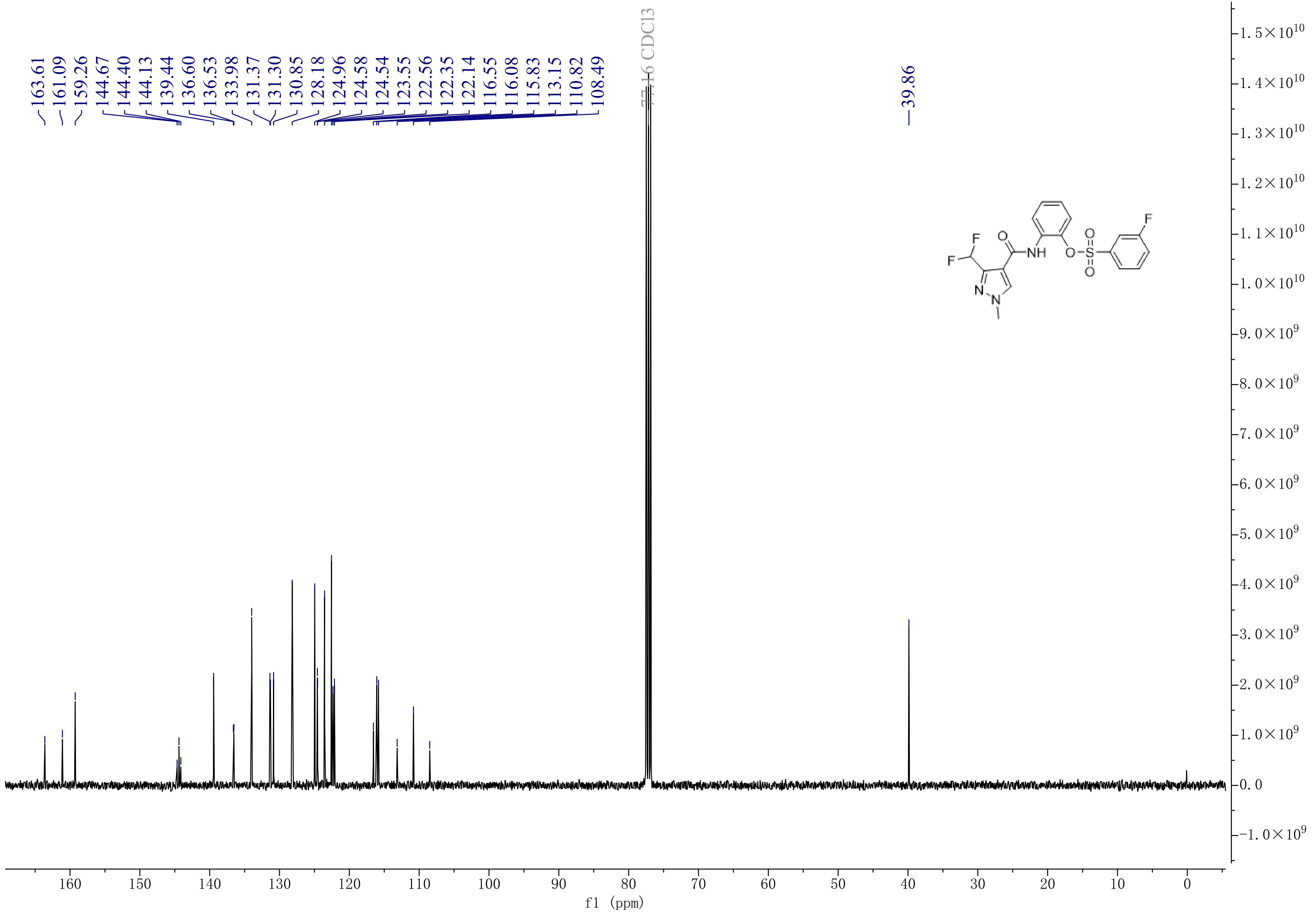
163.61
161.09
159.26
144.67
144.40
144.13
139.44
136.60
136.53
133.98
131.37
131.30
130.85
128.18
124.96
124.58
124.54
123.55
122.56
122.35
122.14
116.55
116.08
115.83
113.15
110.82
108.49
77.16 CDCl3
39.86
f1 (ppm)

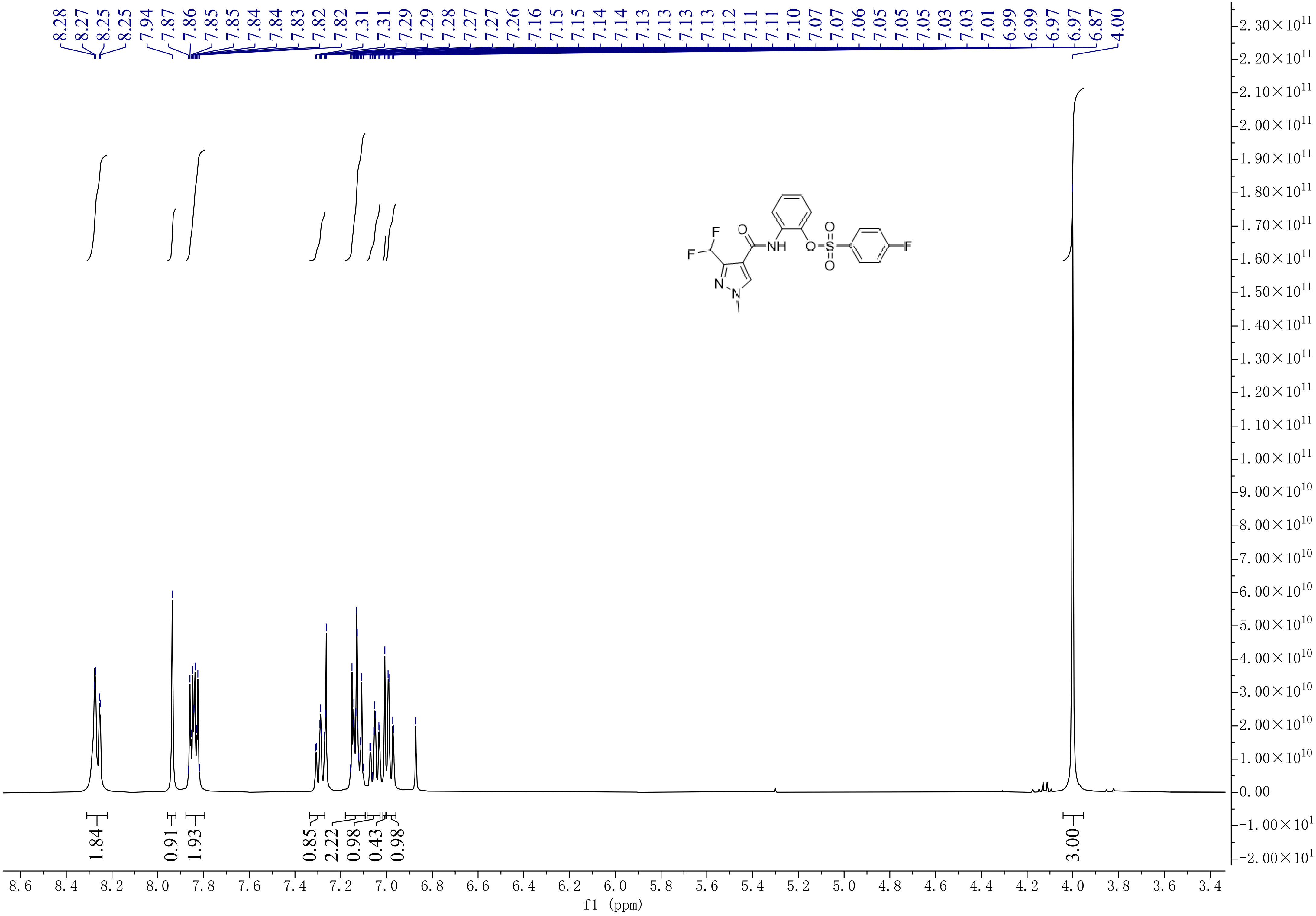
8.28 8.27 8.25 8.25 7.94 7.87 7.86 7.85 7.85 7.84 7.84 7.83 7.82 7.82 7.31 7.31 7.29 7.29 7.28 7.27 7.27 7.26 7.16 7.15 7.15 7.14 7.14 7.13 7.13 7.13 7.13 7.12 7.11 7.11 7.10 7.07 7.07 7.06 7.05 7.05 7.05 7.03 7.03 7.01 6.99 6.99 6.97 6.97 6.87 4.00
1.84
0.91
1.93
0.85
2.22
0.98
0.43
0.98
3.00
f1 (ppm)

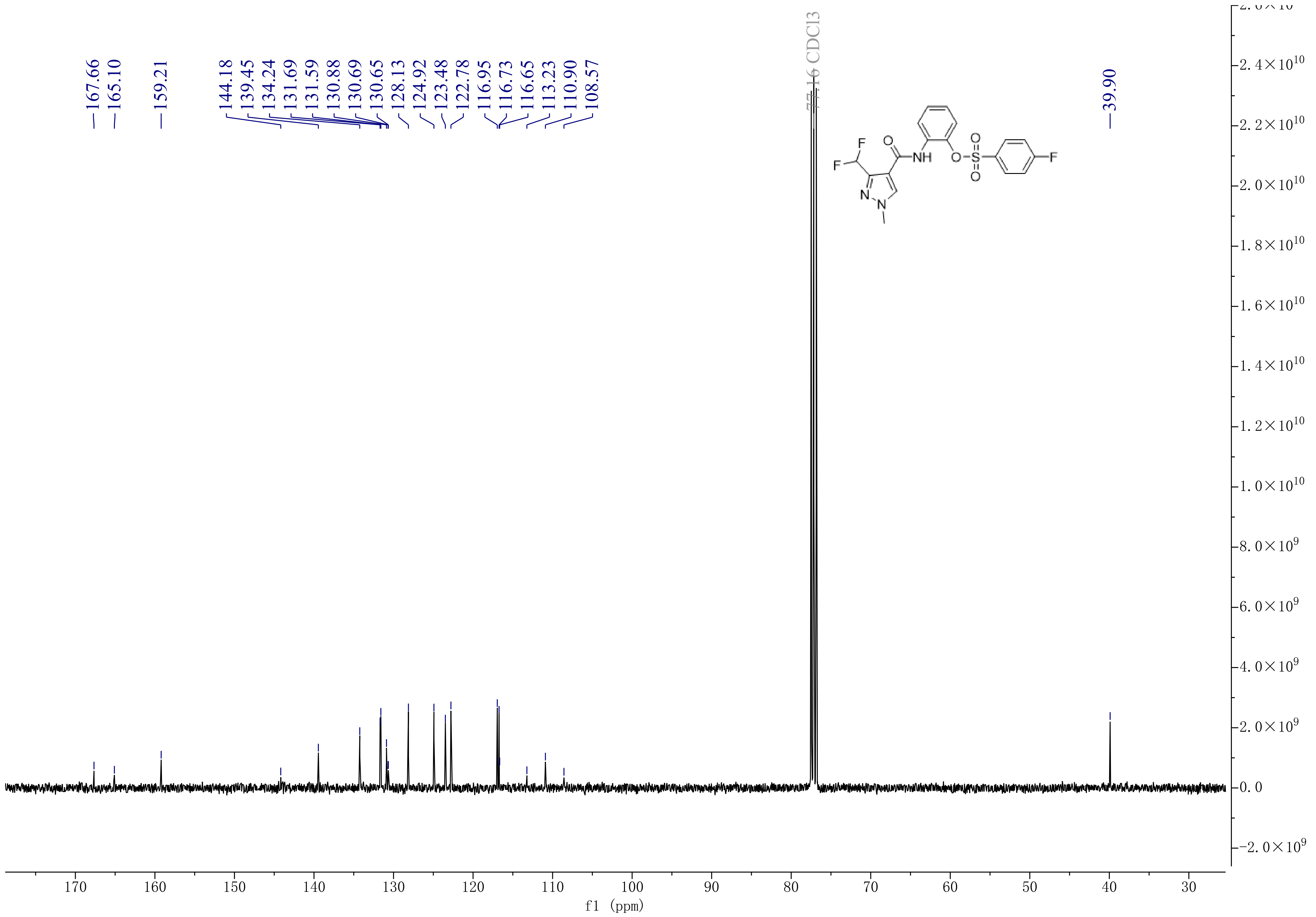
167.66
165.10
159.21
144.18
139.45
134.24
131.69
131.59
130.88
130.69
130.65
128.13
124.92
123.48
122.78
116.95
116.73
116.65
113.23
110.90
108.57
77.16 CDCl3
39.90
F
F
O
NH
O
S
O
O
F
N
N
170
160
150
140
130
120
110
100
90
80
70
60
50
40
30
f1 (ppm)
2.4×10^10
2.2×10^10
2.0×10^10
1.8×10^10
1.6×10^10
1.4×10^10
1.2×10^10
1.0×10^10
8.0×10^9
6.0×10^9
4.0×10^9
2.0×10^9
0.0
-2.0×10^9

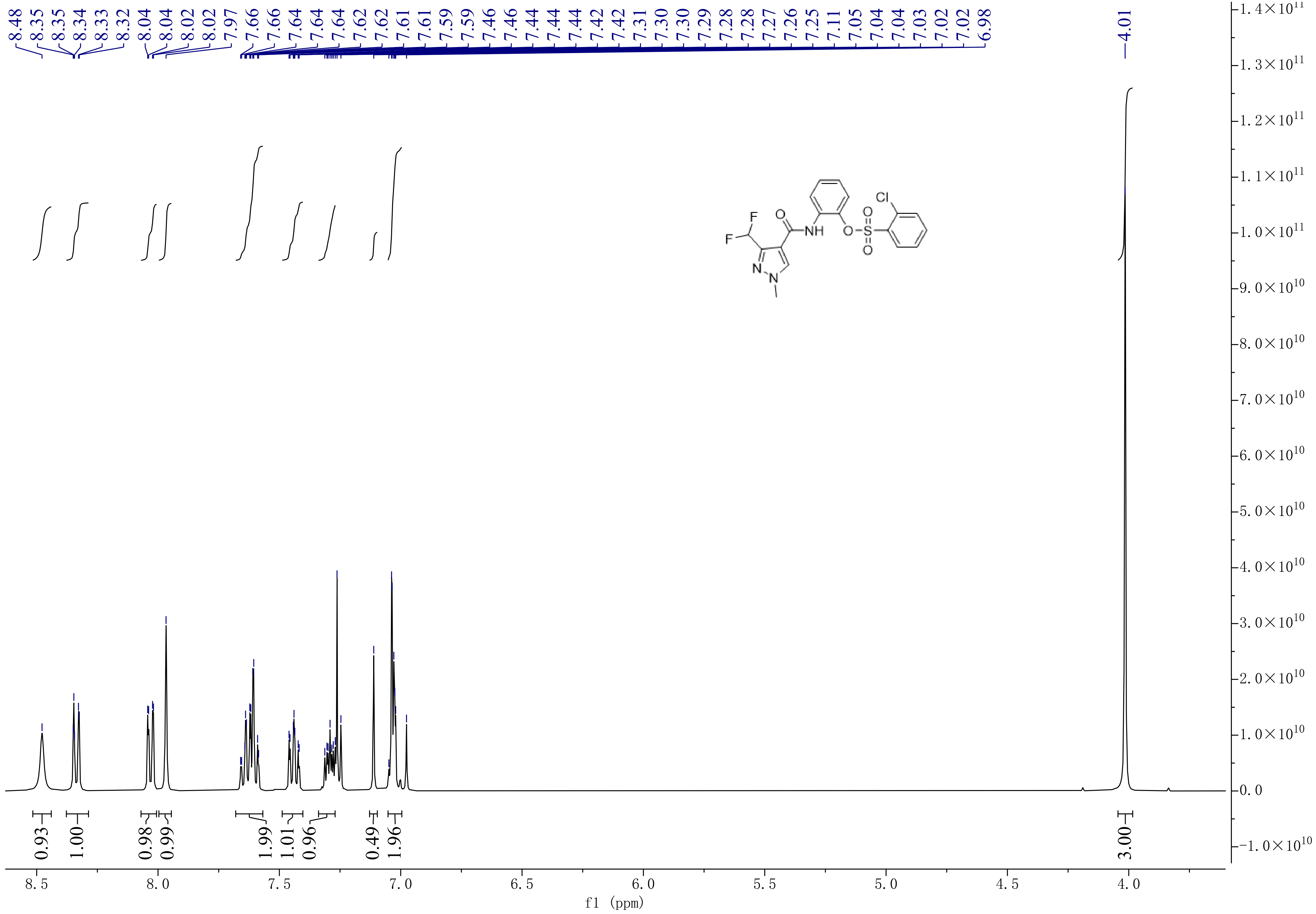
8.48 8.35 8.35 8.34 8.33 8.32 8.04 8.04 8.02 8.02 7.97 7.66 7.66 7.64 7.64 7.64 7.62 7.62 7.61 7.61 7.59 7.59 7.46 7.46 7.44 7.44 7.44 7.42 7.42 7.31 7.30 7.30 7.29 7.28 7.28 7.27 7.26 7.25 7.11 7.05 7.04 7.04 7.03 7.02 7.02 6.98
4.01
F
F
O
NH
O
S
O
O
Cl
N
N
0.93 1.00 0.98 0.99 1.99 1.01 0.96 0.49 1.96 3.00
8.5 8.0 7.5 7.0 6.5 6.0 5.5 5.0 4.5 4.0
f1 (ppm)
1.4×10^11 1.3×10^11 1.2×10^11 1.1×10^11 1.0×10^11 9.0×10^10 8.0×10^10 7.0×10^10 6.0×10^10 5.0×10^10 4.0×10^10 3.0×10^10 2.0×10^10 1.0×10^10 0.0 −1.0×10^10

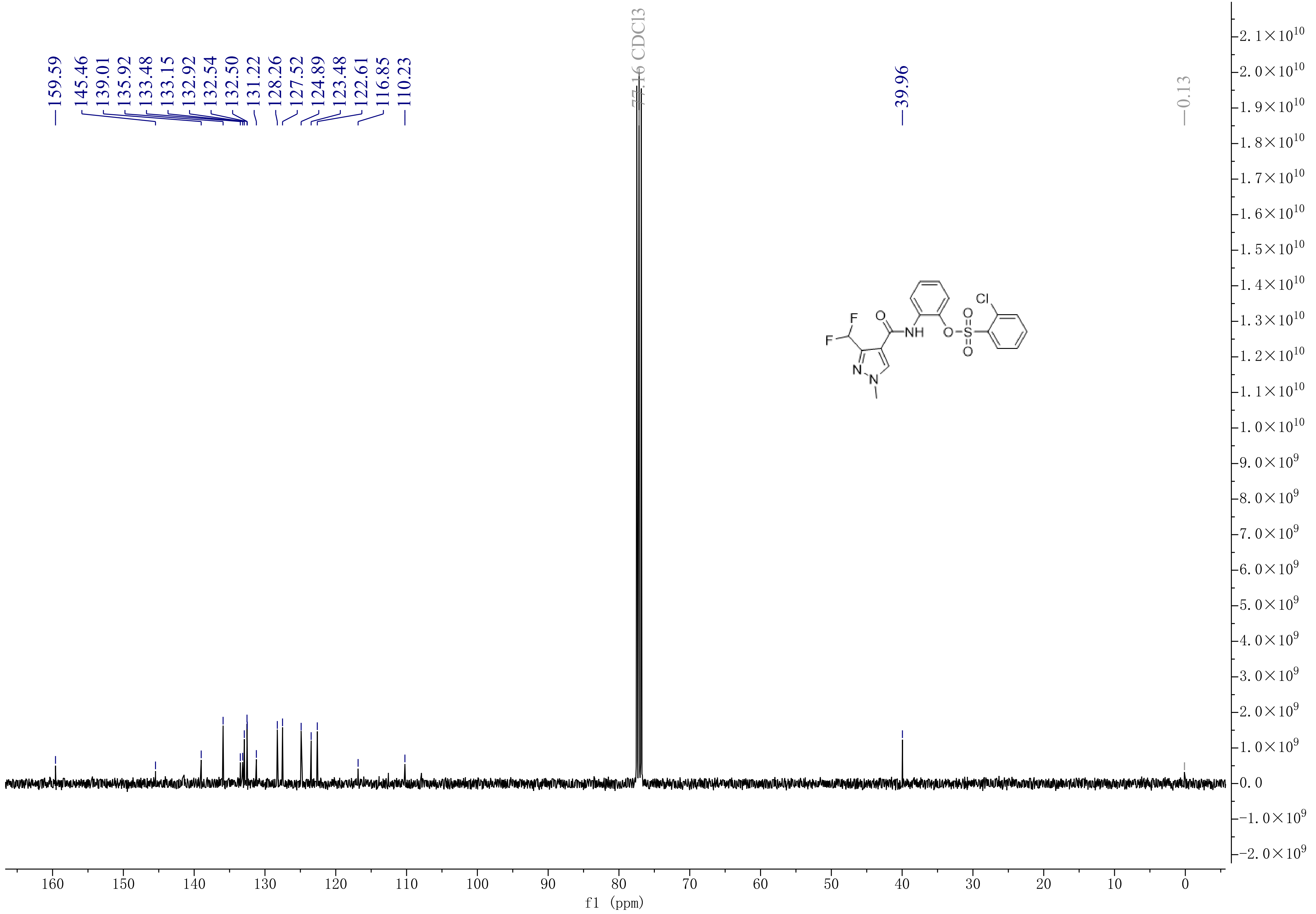
159.59
145.46
139.01
135.92
133.48
133.15
132.92
132.54
132.50
131.22
128.26
127.52
124.89
123.48
122.61
116.85
110.23
77.16 CDCl3
39.96
0.13
F
F
O
NH
O
S
O
O
Cl
N
N
f1 (ppm)

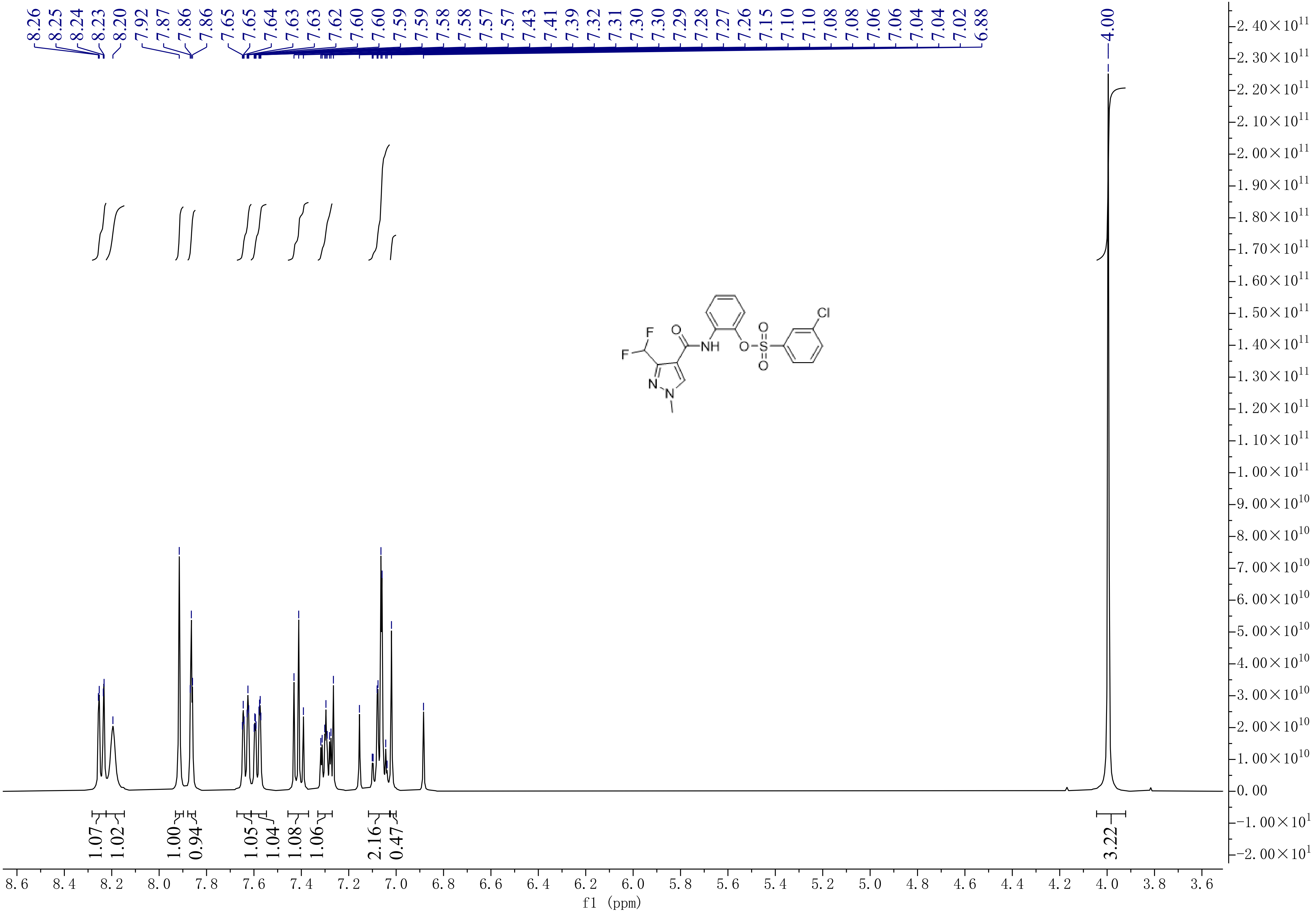
8.26 8.25 8.24 8.23 8.20 7.92 7.87 7.86 7.86 7.65 7.65 7.64 7.63 7.63 7.62 7.60 7.60 7.59 7.59 7.58 7.58 7.57 7.57 7.43 7.41 7.39 7.32 7.31 7.30 7.30 7.29 7.28 7.27 7.26 7.15 7.10 7.10 7.08 7.08 7.06 7.06 7.04 7.04 7.02 6.88
4.00
1.07 1.02 1.00 0.94 1.05 1.04 1.08 1.06 2.16 0.47 3.22
F
F
O
NH
O
S
O
O
Cl
N
N
f1 (ppm)

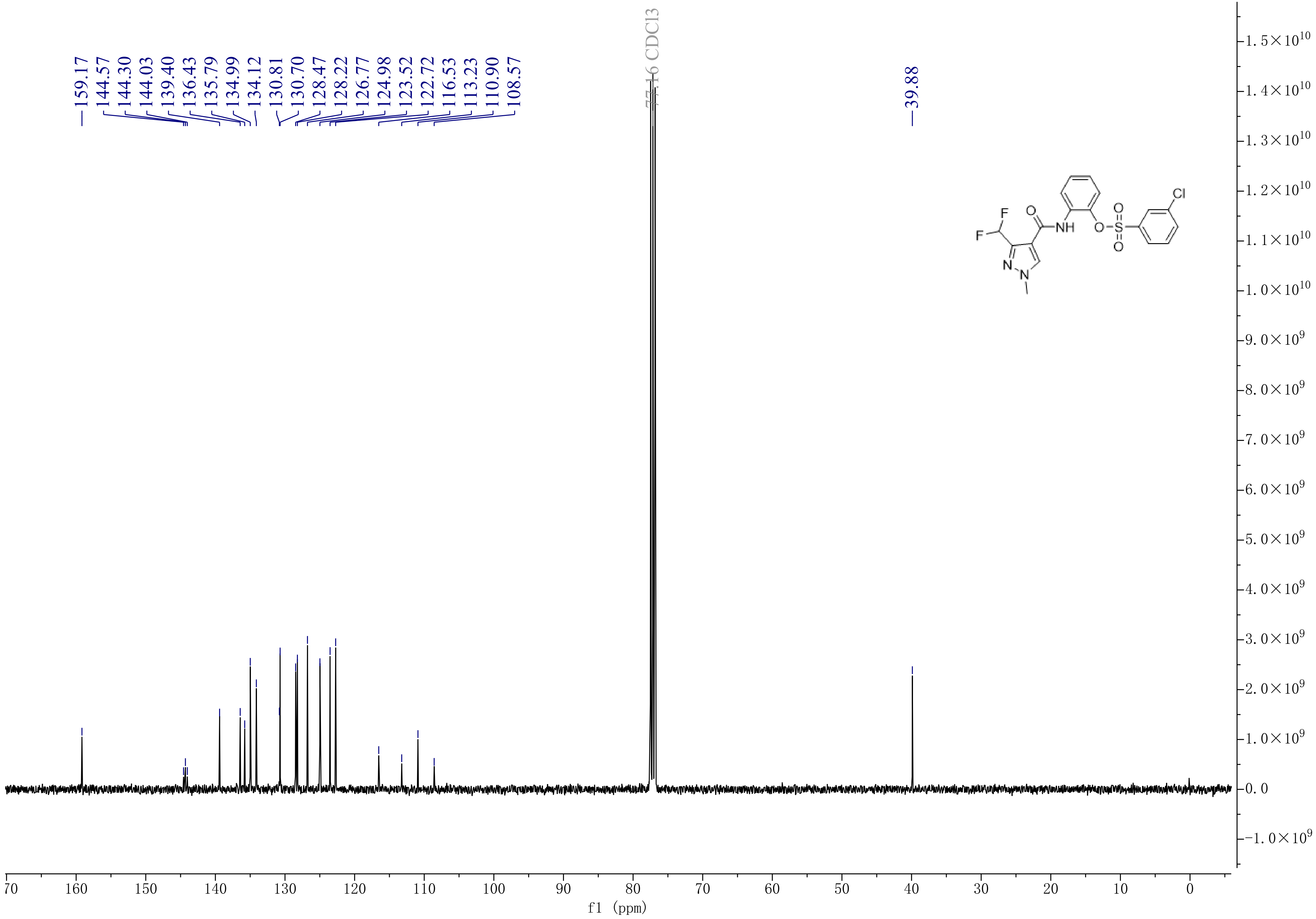
159.17
144.57
144.30
144.03
139.40
136.43
135.79
134.99
134.12
130.81
130.70
128.47
128.22
126.77
124.98
123.52
122.72
116.53
113.23
110.90
108.57
77.16 CDCl3
39.88
F
F
O
NH
O
S
O
O
Cl
N
N
f1 (ppm)

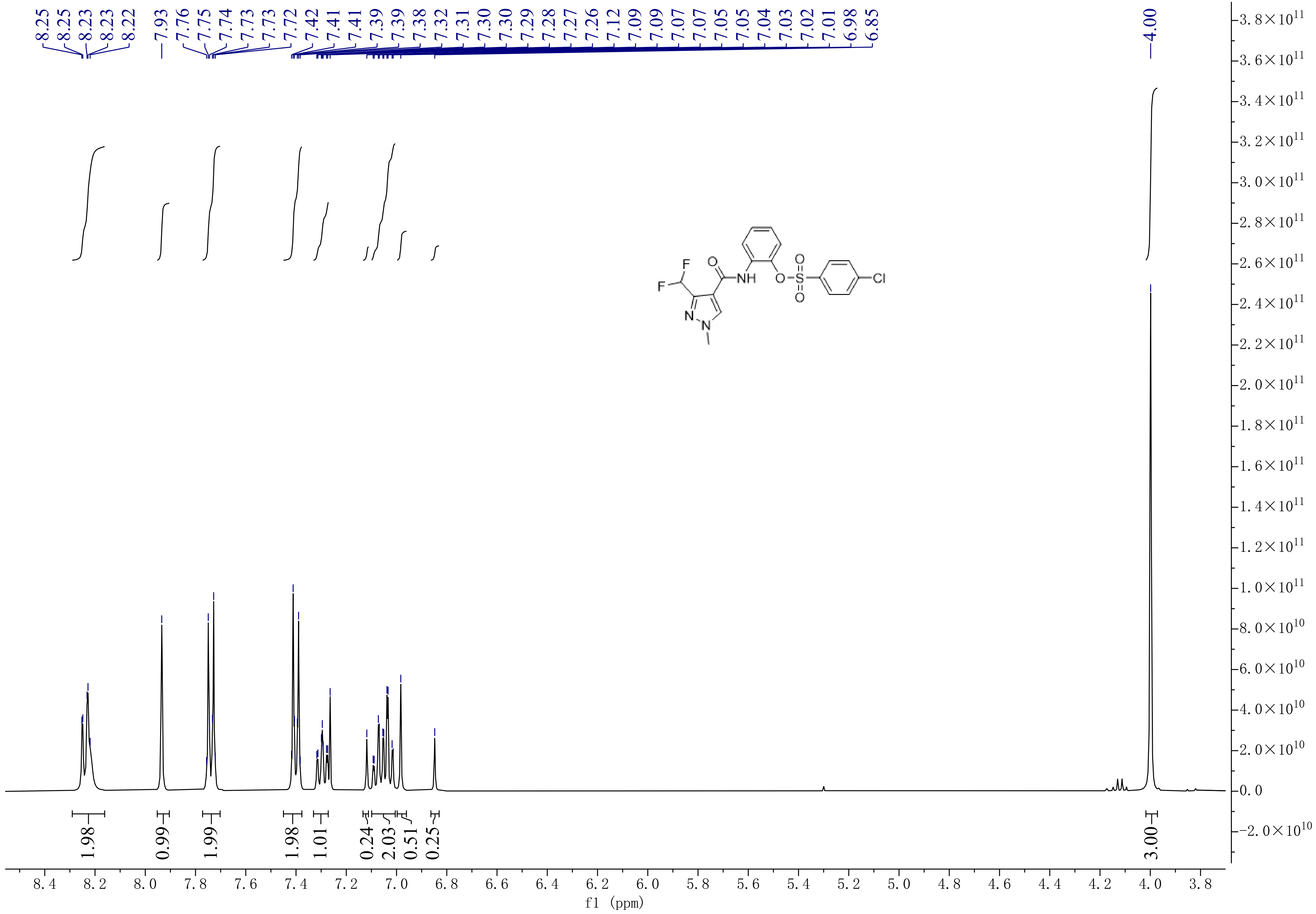
8.25
8.25
8.23
8.23
8.22
7.93
7.76
7.75
7.74
7.73
7.73
7.72
7.42
7.41
7.41
7.39
7.39
7.38
7.32
7.31
7.30
7.30
7.29
7.28
7.27
7.26
7.12
7.09
7.09
7.07
7.07
7.05
7.05
7.04
7.03
7.02
7.01
6.98
6.85
4.00
F
F
O
NH
O
S
O
O
Cl
N
N
1.98
0.99
1.99
1.98
1.01
0.24
2.03
0.51
0.25
3.00
f1 (ppm)

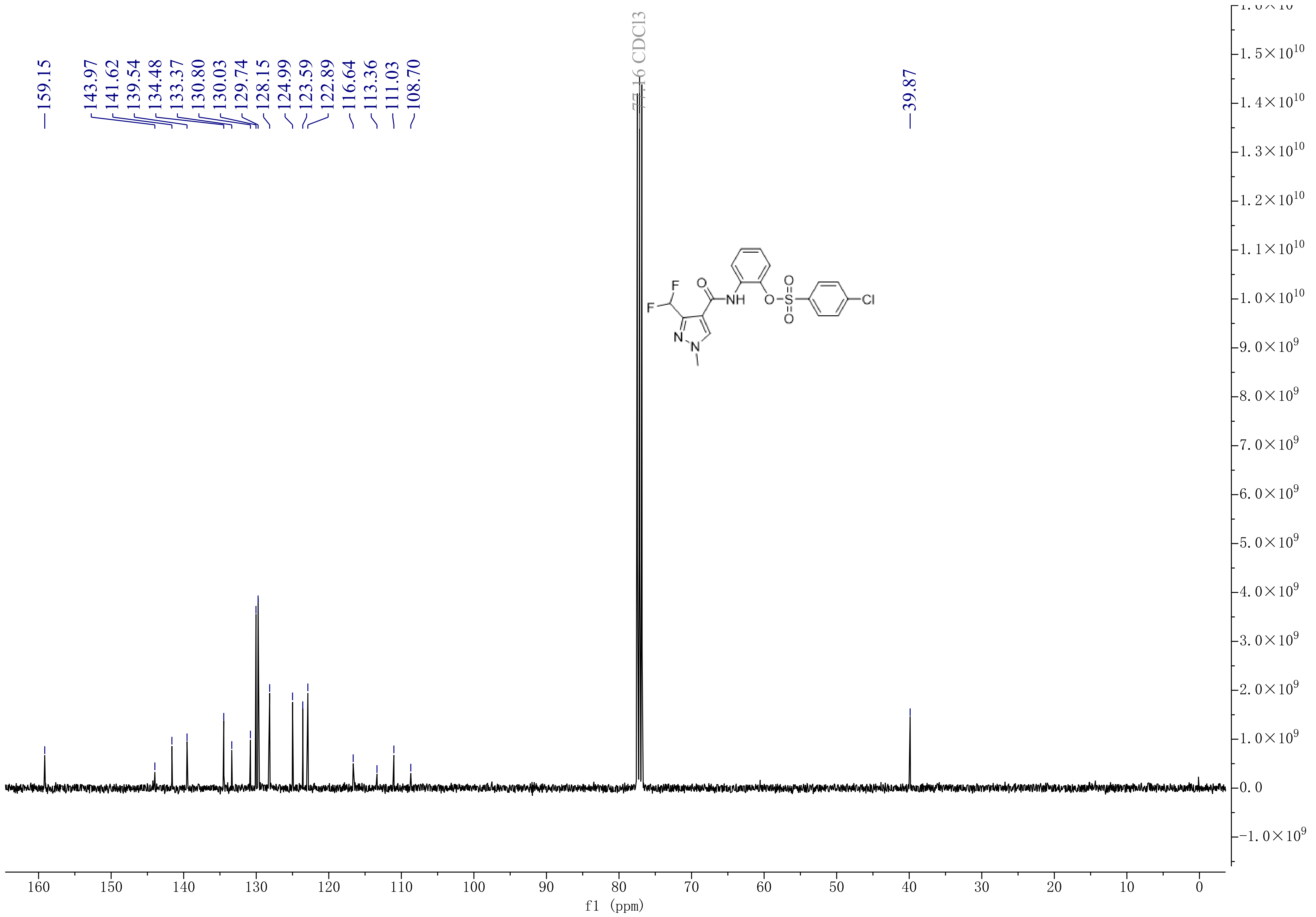
159.15
143.97
141.62
139.54
134.48
133.37
130.80
130.03
129.74
128.15
124.99
123.59
122.89
116.64
113.36
111.03
108.70
77.16 CDCl3
39.87
F
F
O
NH
O
S
O
O
Cl
N
N
f1 (ppm)

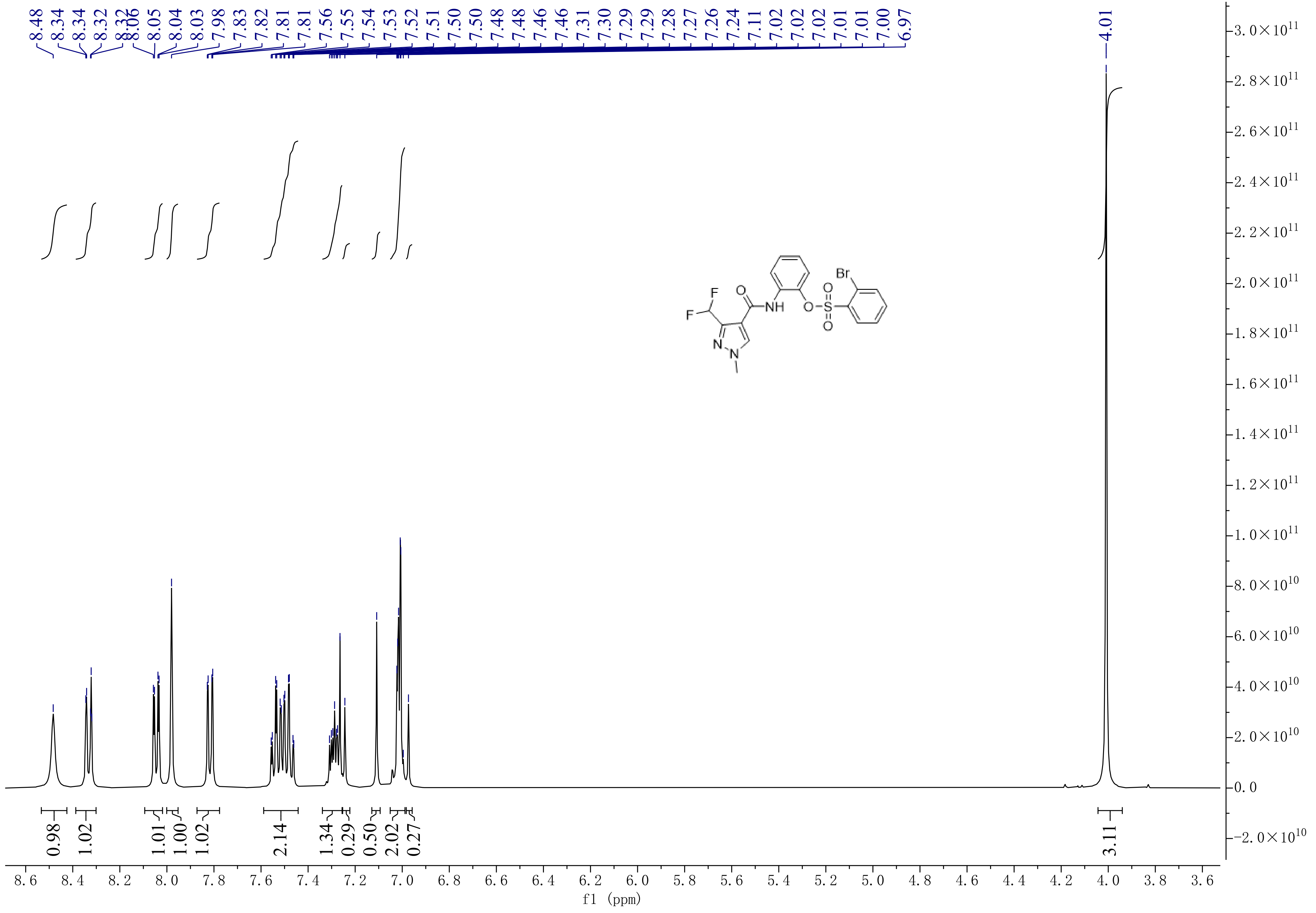

8.48 8.34 8.34 8.32 8.32 8.06 8.05 8.04 8.03 7.98 7.83 7.82 7.81 7.81 7.56 7.55 7.54 7.53 7.52 7.51 7.50 7.50 7.48 7.48 7.46 7.46 7.31 7.30 7.29 7.29 7.28 7.27 7.26 7.24 7.11 7.02 7.02 7.02 7.01 7.01 7.00 6.97
4.01
0.98 1.02 1.01 1.00 1.02 2.14 1.34 0.29 0.50 2.02 0.27 3.11
f1 (ppm)
F
F
O
NH
Br
O
S
O
O
N
N

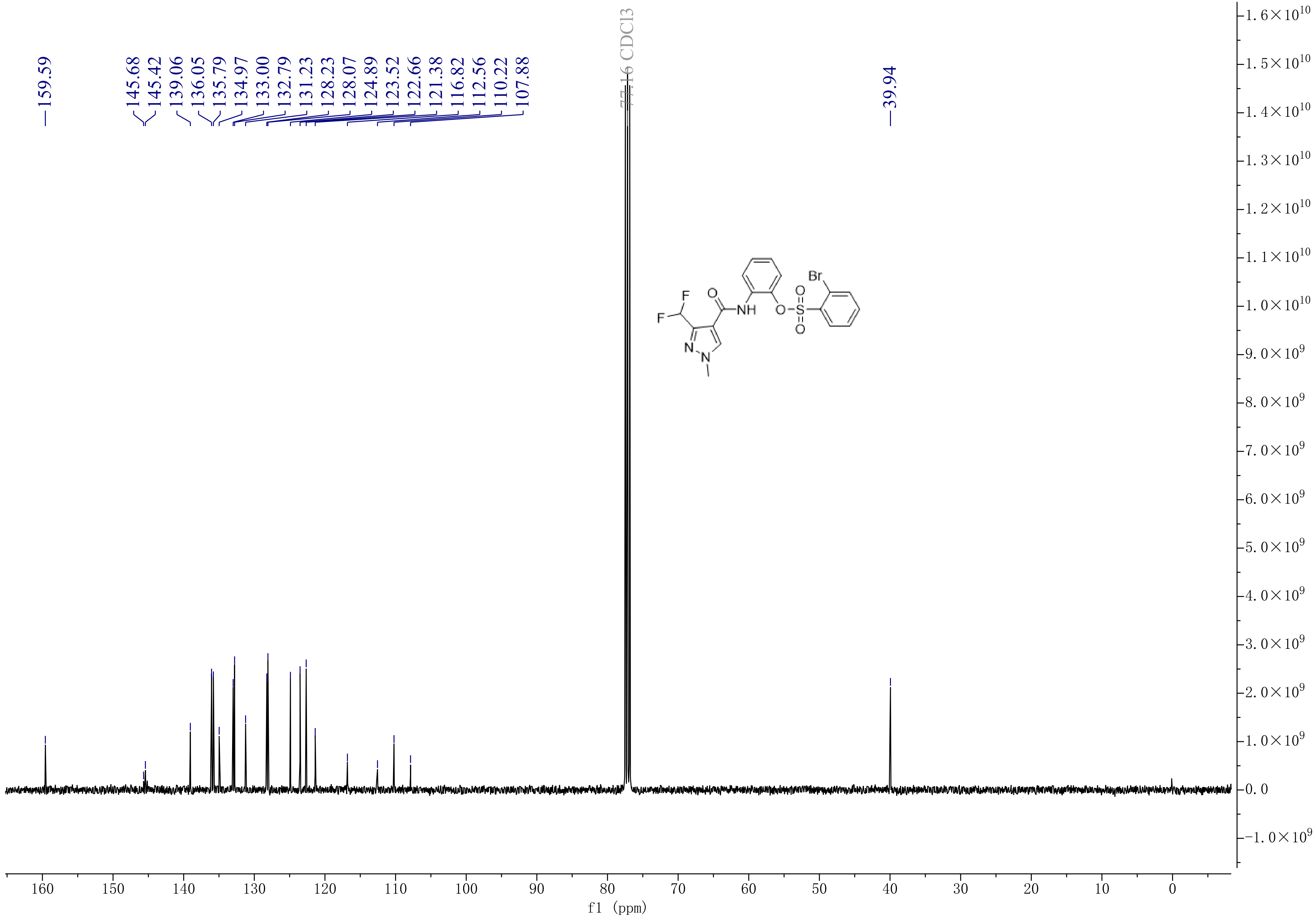

159.59
145.68
145.42
139.06
136.05
135.79
134.97
133.00
132.79
131.23
128.23
128.07
124.89
123.52
122.66
121.38
116.82
112.56
110.22
107.88
77.16 CDCl3
39.94
F
F
O
NH
O
Br
O
S
O
N
N
1.6×10^10
1.5×10^10
1.4×10^10
1.3×10^10
1.2×10^10
1.1×10^10
1.0×10^10
9.0×10^9
8.0×10^9
7.0×10^9
6.0×10^9
5.0×10^9
4.0×10^9
3.0×10^9
2.0×10^9
1.0×10^9
0.0
-1.0×10^9
160
150
140
130
120
110
100
90
80
70
60
50
40
30
20
10
0
f1 (ppm)

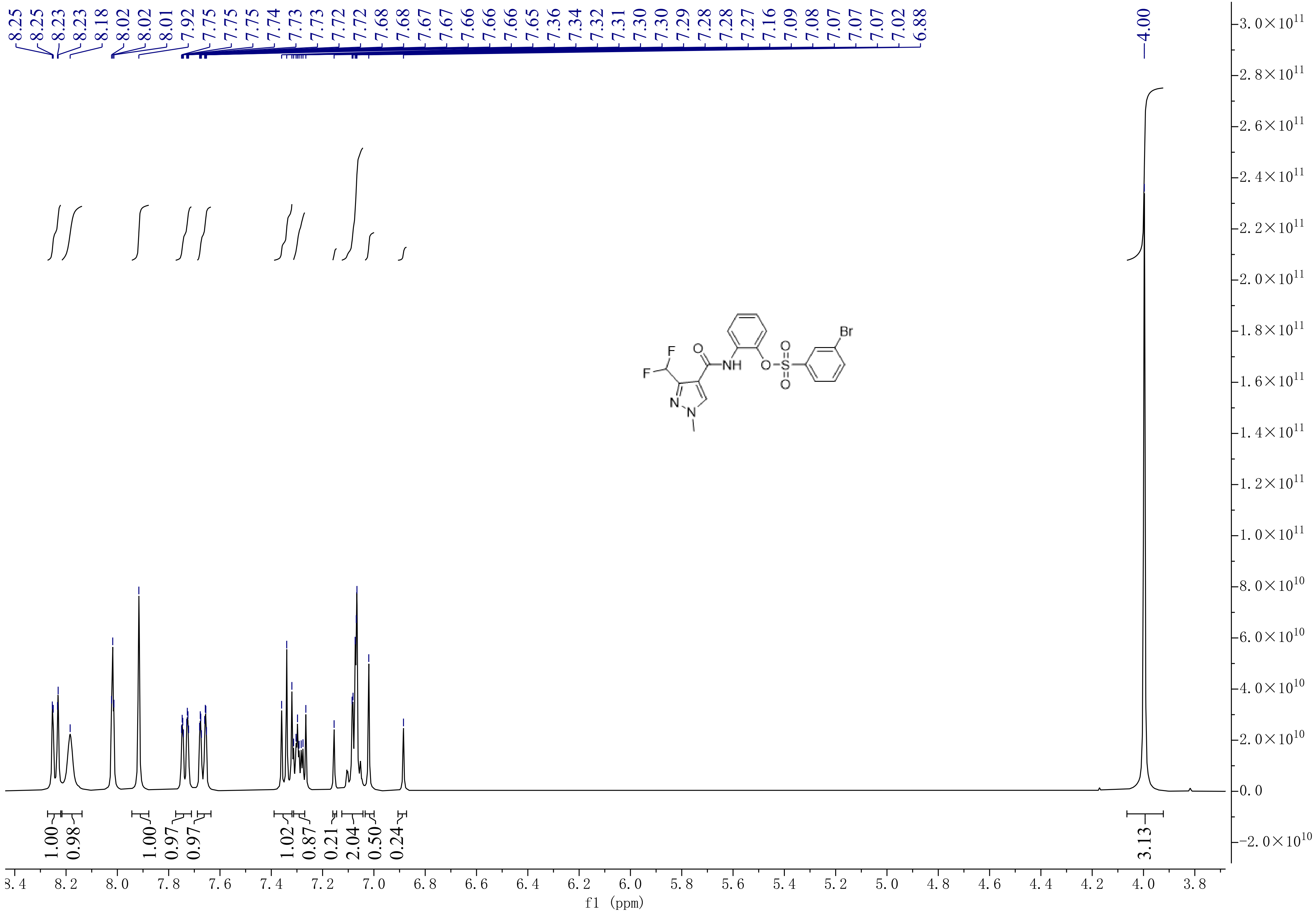

8.25 8.25 8.23 8.23 8.18 8.02 8.02 8.01 7.92 7.75 7.75 7.75 7.74 7.73 7.73 7.72 7.72 7.68 7.68 7.67 7.67 7.66 7.66 7.66 7.65 7.36 7.34 7.32 7.31 7.30 7.30 7.29 7.28 7.28 7.27 7.16 7.09 7.08 7.07 7.07 7.07 7.02 6.88
4.00
1.00 0.98 1.00 0.97 0.97 1.02 0.87 0.21 2.04 0.50 0.24
3.13
F
F
O
NH
O
S
O
O
Br
N
N
f1 (ppm)

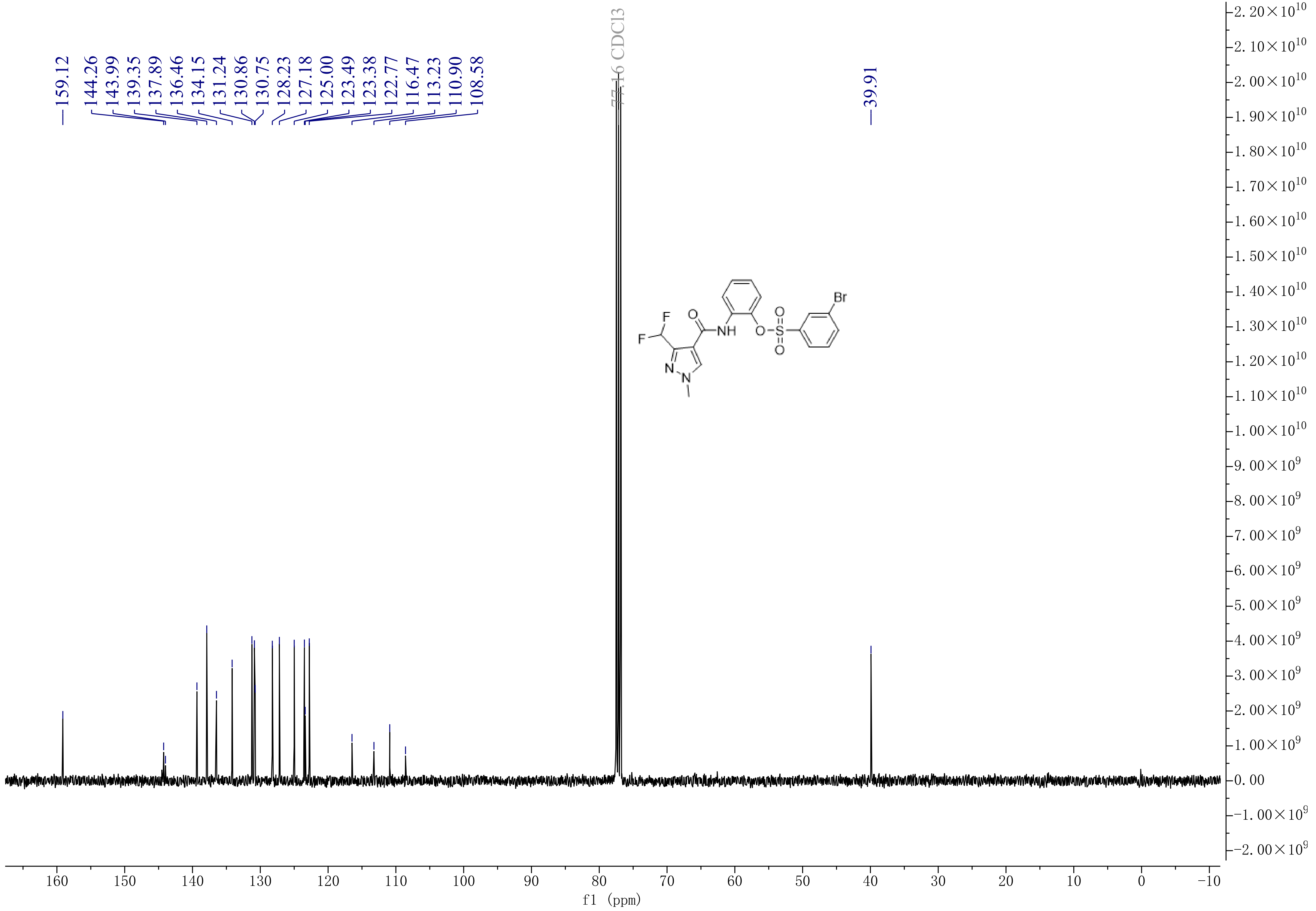

159.12
144.26
143.99
139.35
137.89
136.46
134.15
131.24
130.86
130.75
128.23
127.18
125.00
123.49
123.38
122.77
116.47
113.23
110.90
108.58
77.16 CDCl3
39.91
F
F
O
NH
N
N
O
S
O
O
Br
f1 (ppm)

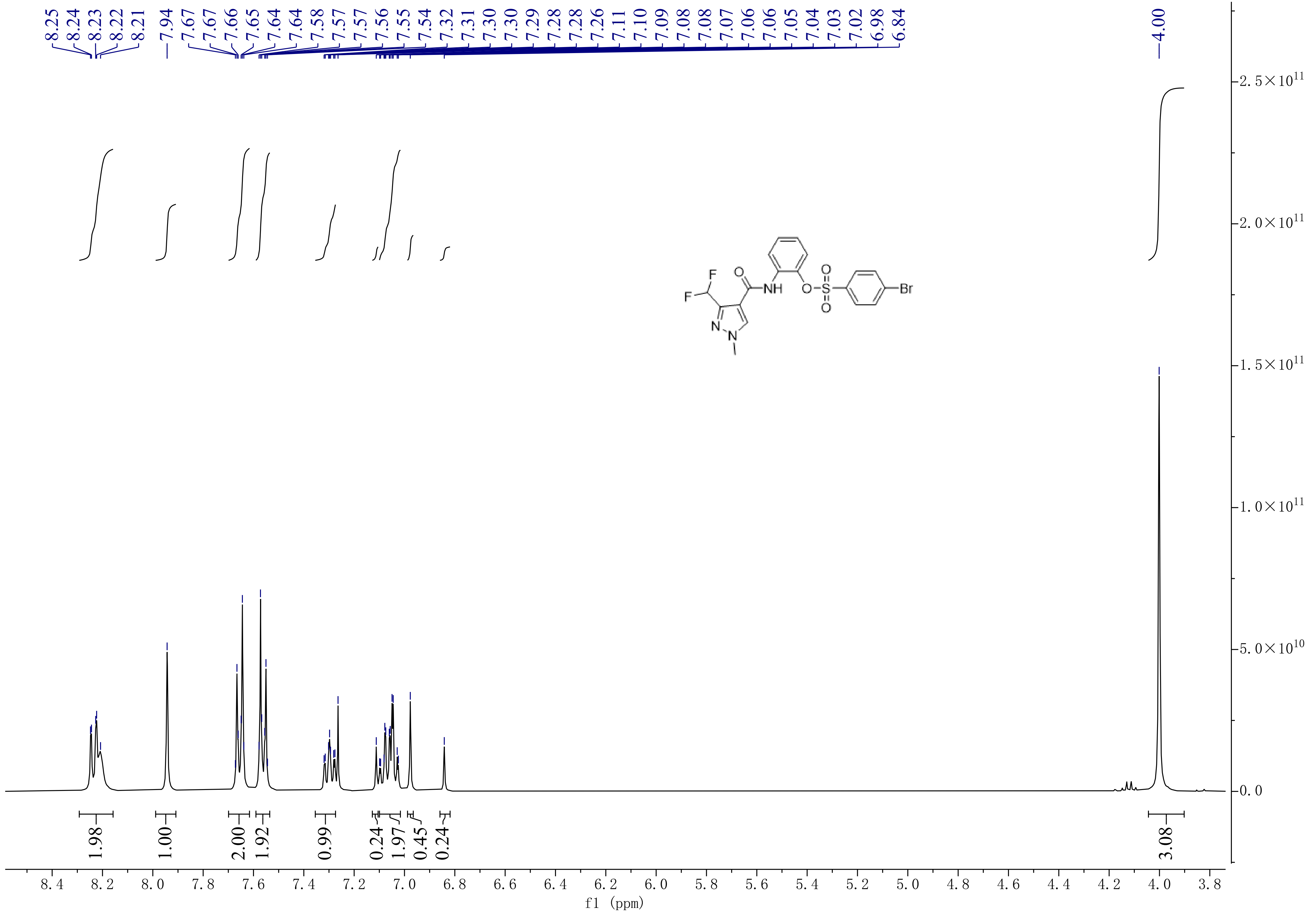
8.25 8.24 8.23 8.22 8.21 7.94 7.67 7.67 7.66 7.65 7.64 7.64 7.58 7.57 7.57 7.56 7.55 7.54 7.32 7.31 7.30 7.30 7.29 7.28 7.28 7.26 7.11 7.10 7.09 7.08 7.08 7.07 7.06 7.06 7.05 7.04 7.03 7.02 6.98 6.84
4.00
F
F
O
NH
O
S
O
O
Br
N
N
1.98
1.00
2.00
1.92
0.99
0.24
1.97
0.45
0.24
3.08
2.5×10^11
2.0×10^11
1.5×10^11
1.0×10^11
5.0×10^10
0.0
8.4 8.2 8.0 7.8 7.6 7.4 7.2 7.0 6.8 6.6 6.4 6.2 6.0 5.8 5.6 5.4 5.2 5.0 4.8 4.6 4.4 4.2 4.0 3.8
f1 (ppm)

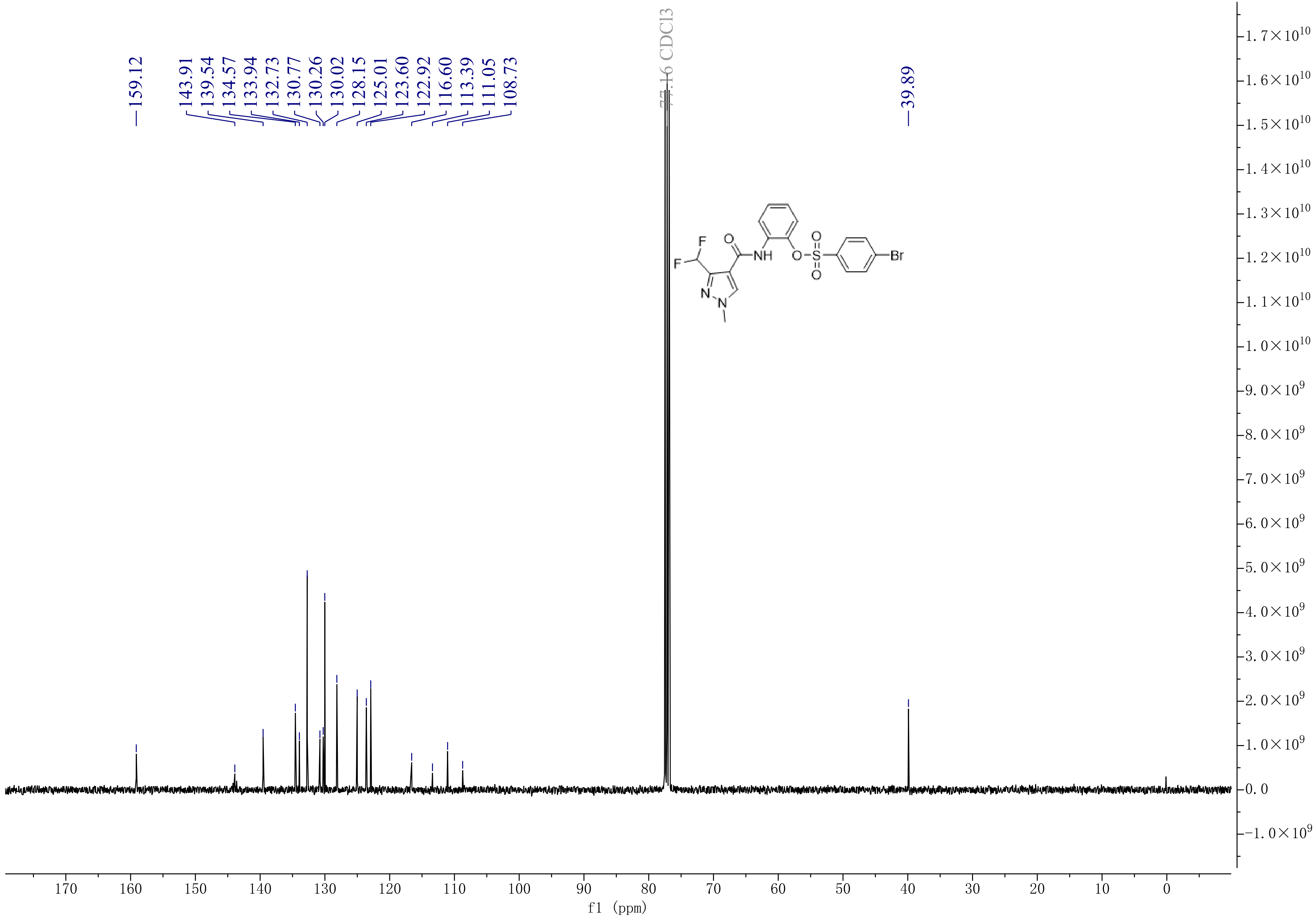

159.12
143.91
139.54
134.57
133.94
132.73
130.77
130.26
130.02
128.15
125.01
123.60
122.92
116.60
113.39
111.05
108.73
77.16 CDCl3
39.89
F
F
O
NH
N
N
O
S
O
O
Br
1.7×10^10
1.6×10^10
1.5×10^10
1.4×10^10
1.3×10^10
1.2×10^10
1.1×10^10
1.0×10^10
9.0×10^9
8.0×10^9
7.0×10^9
6.0×10^9
5.0×10^9
4.0×10^9
3.0×10^9
2.0×10^9
1.0×10^9
0.0
−1.0×10^9
170
160
150
140
130
120
110
100
90
80
70
60
50
40
30
20
10
0
f1 (ppm)

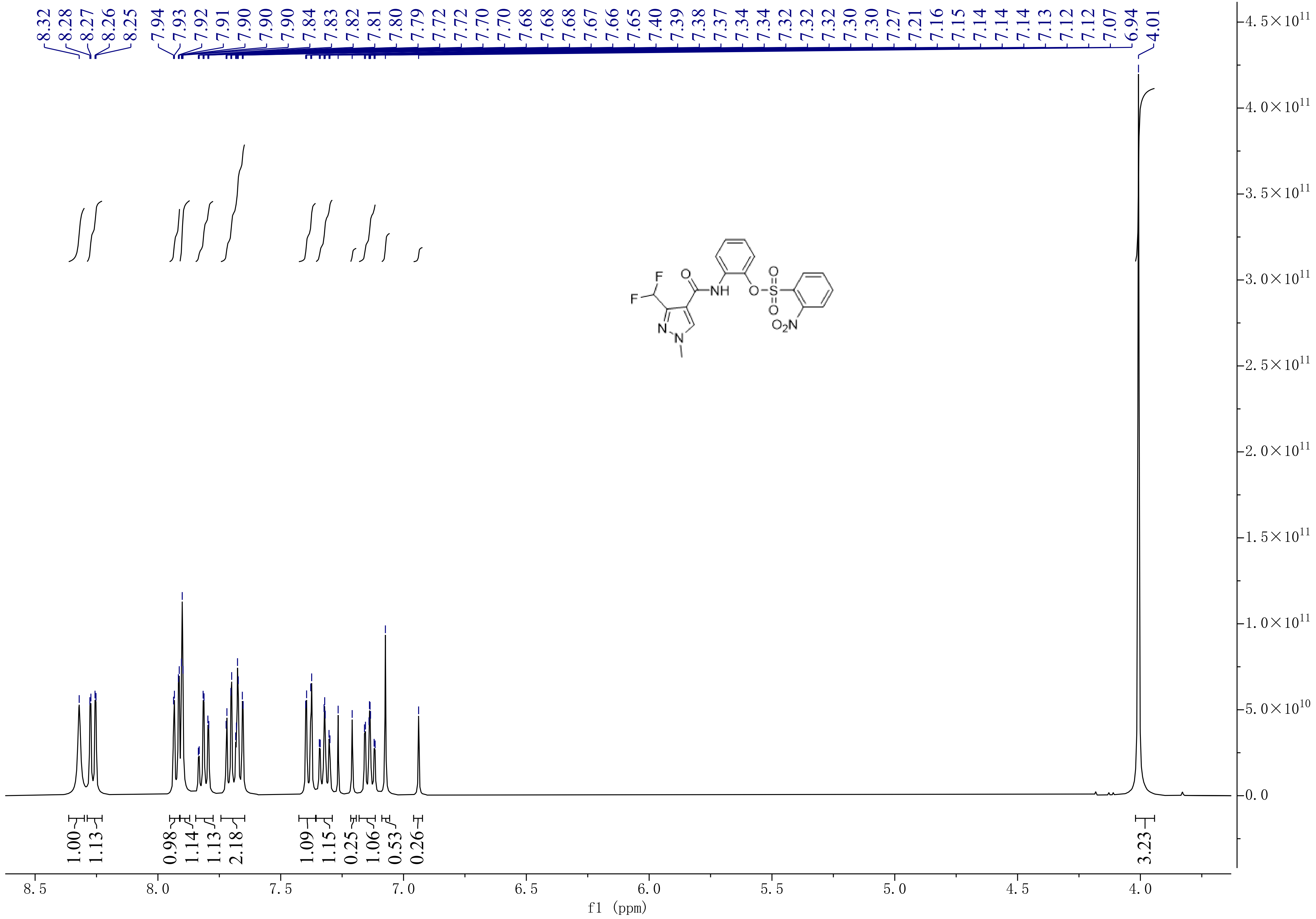
8.32
8.28
8.27
8.26
8.25
7.94
7.93
7.92
7.91
7.90
7.90
7.90
7.84
7.83
7.82
7.81
7.80
7.79
7.72
7.72
7.70
7.70
7.68
7.68
7.68
7.67
7.66
7.65
7.40
7.39
7.38
7.37
7.34
7.34
7.32
7.32
7.32
7.30
7.30
7.27
7.21
7.16
7.15
7.14
7.14
7.14
7.13
7.12
7.12
7.07
6.94
4.01
1.00
1.13
0.98
1.14
1.13
2.18
1.09
1.15
0.25
1.06
0.53
0.26
3.23
F
F
O
NH
O
S
O
O
N
N
O2N
f1 (ppm)

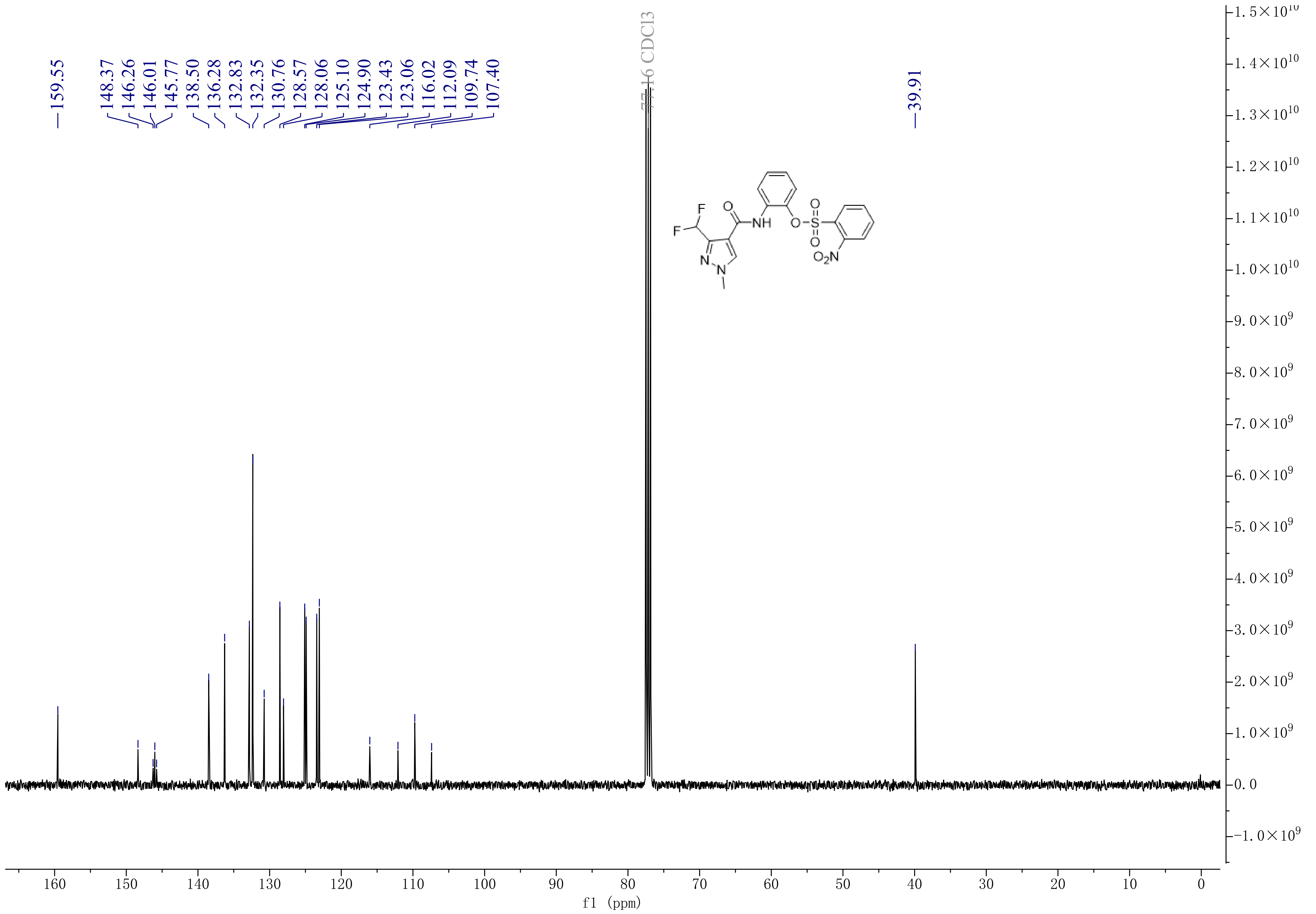

159.55
148.37
146.26
146.01
145.77
138.50
136.28
132.83
132.35
130.76
128.57
128.06
125.10
124.90
123.43
123.06
116.02
112.09
109.74
107.40
77.16 CDCl3
39.91
F
F
O
NH
O
S
O
O
N
N
O2N
1.5×10^10
1.4×10^10
1.3×10^10
1.2×10^10
1.1×10^10
1.0×10^10
9.0×10^9
8.0×10^9
7.0×10^9
6.0×10^9
5.0×10^9
4.0×10^9
3.0×10^9
2.0×10^9
1.0×10^9
0.0
−1.0×10^9
160
150
140
130
120
110
100
90
80
70
60
50
40
30
20
10
0
f1 (ppm)

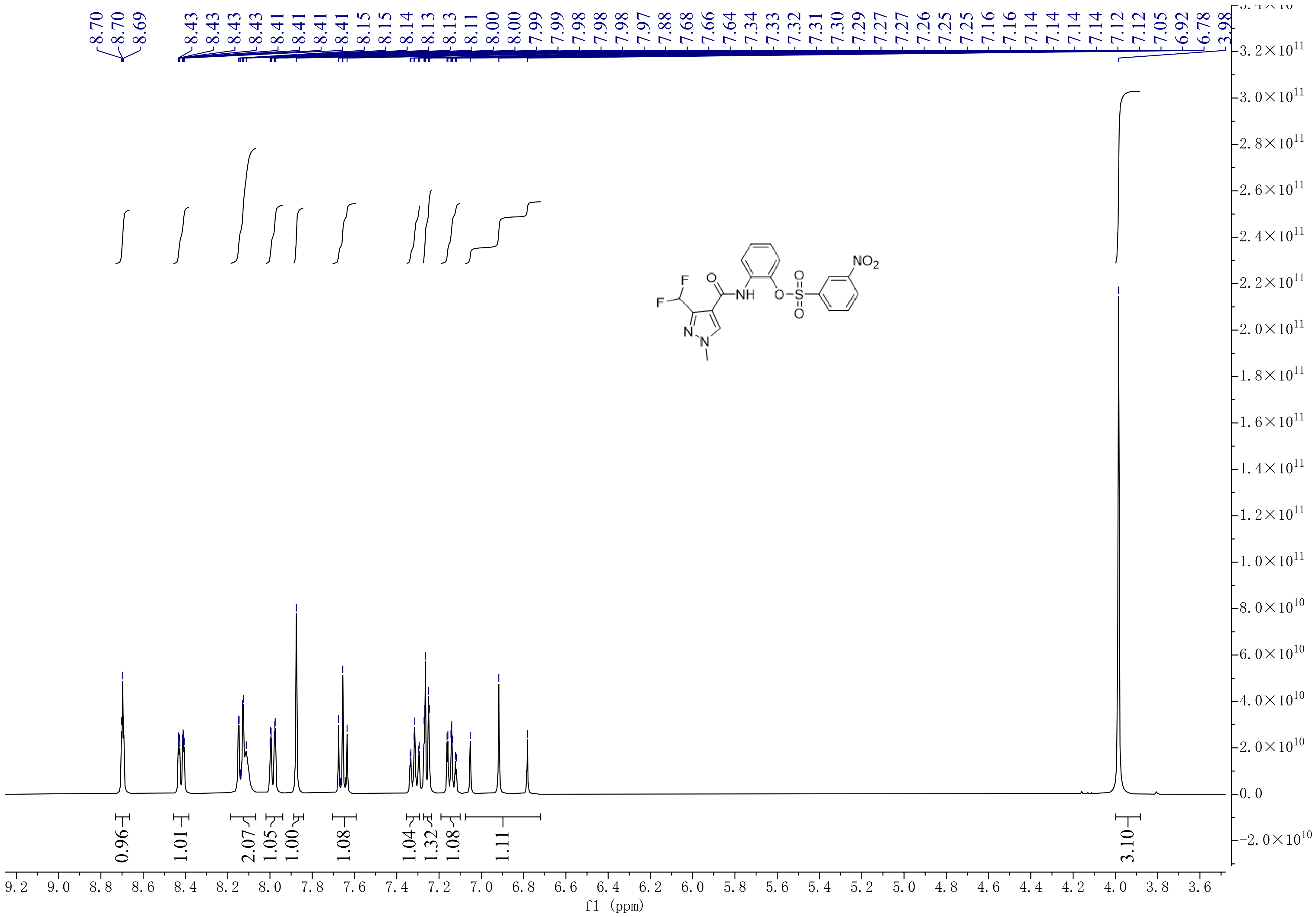
8.70
8.70
8.69
8.43
8.43
8.43
8.43
8.41
8.41
8.41
8.41
8.15
8.15
8.14
8.13
8.13
8.11
8.00
8.00
7.99
7.99
7.98
7.98
7.98
7.97
7.88
7.68
7.66
7.64
7.34
7.33
7.32
7.31
7.30
7.29
7.27
7.27
7.26
7.25
7.25
7.16
7.16
7.14
7.14
7.14
7.14
7.12
7.12
7.05
6.92
6.78
3.98
0.96
1.01
2.07
1.05
1.00
1.08
1.04
1.32
1.08
1.11
3.10
F
F
O
NH
O
S
O
O
NO2
N
N
f1 (ppm)

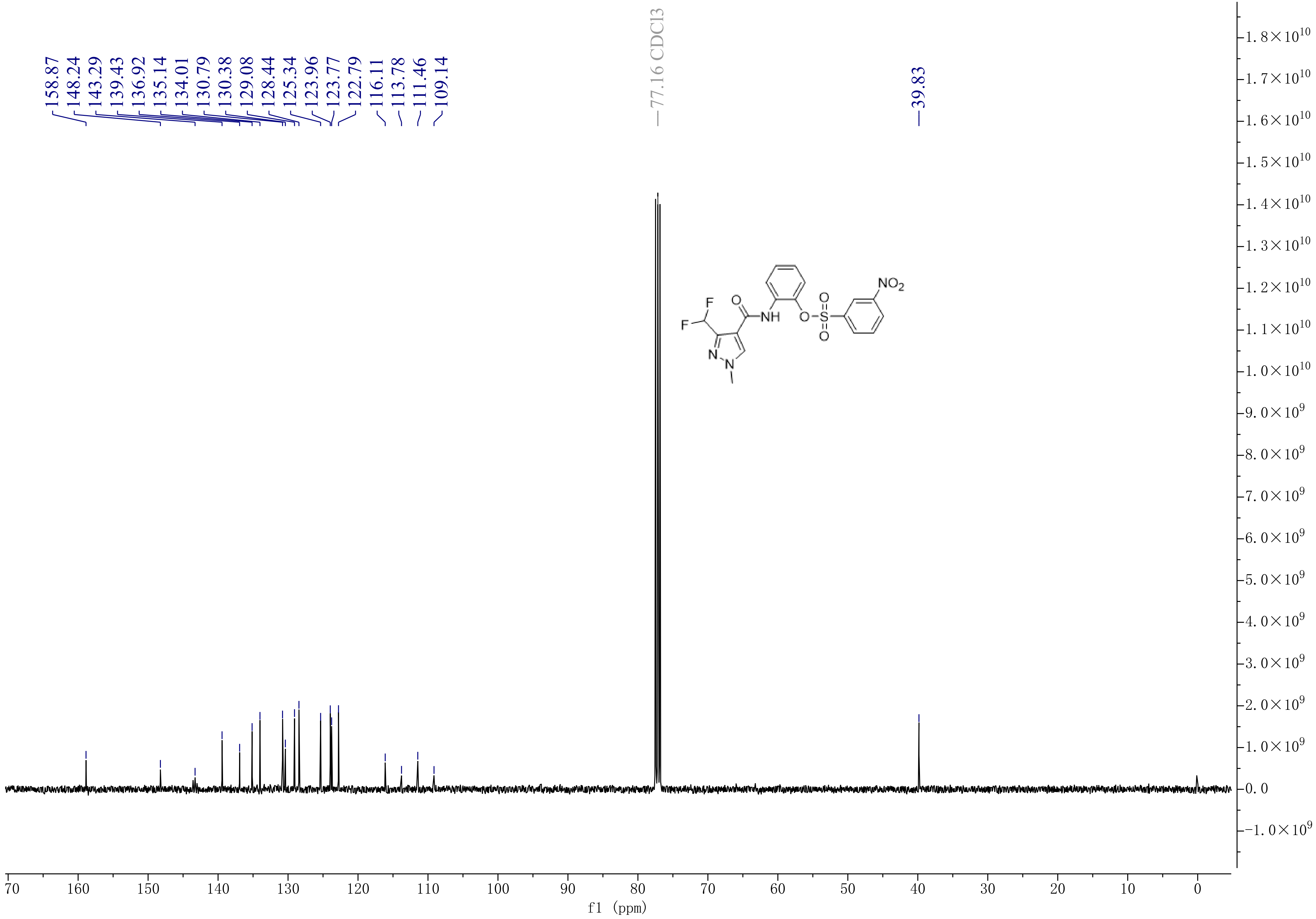

158.87
148.24
143.29
139.43
136.92
135.14
134.01
130.79
130.38
129.08
128.44
125.34
123.96
123.77
122.79
116.11
113.78
111.46
109.14
77.16 CDCl3
39.83
F
F
O
NH
O
S
O
O
NO2
N
N
f1 (ppm)

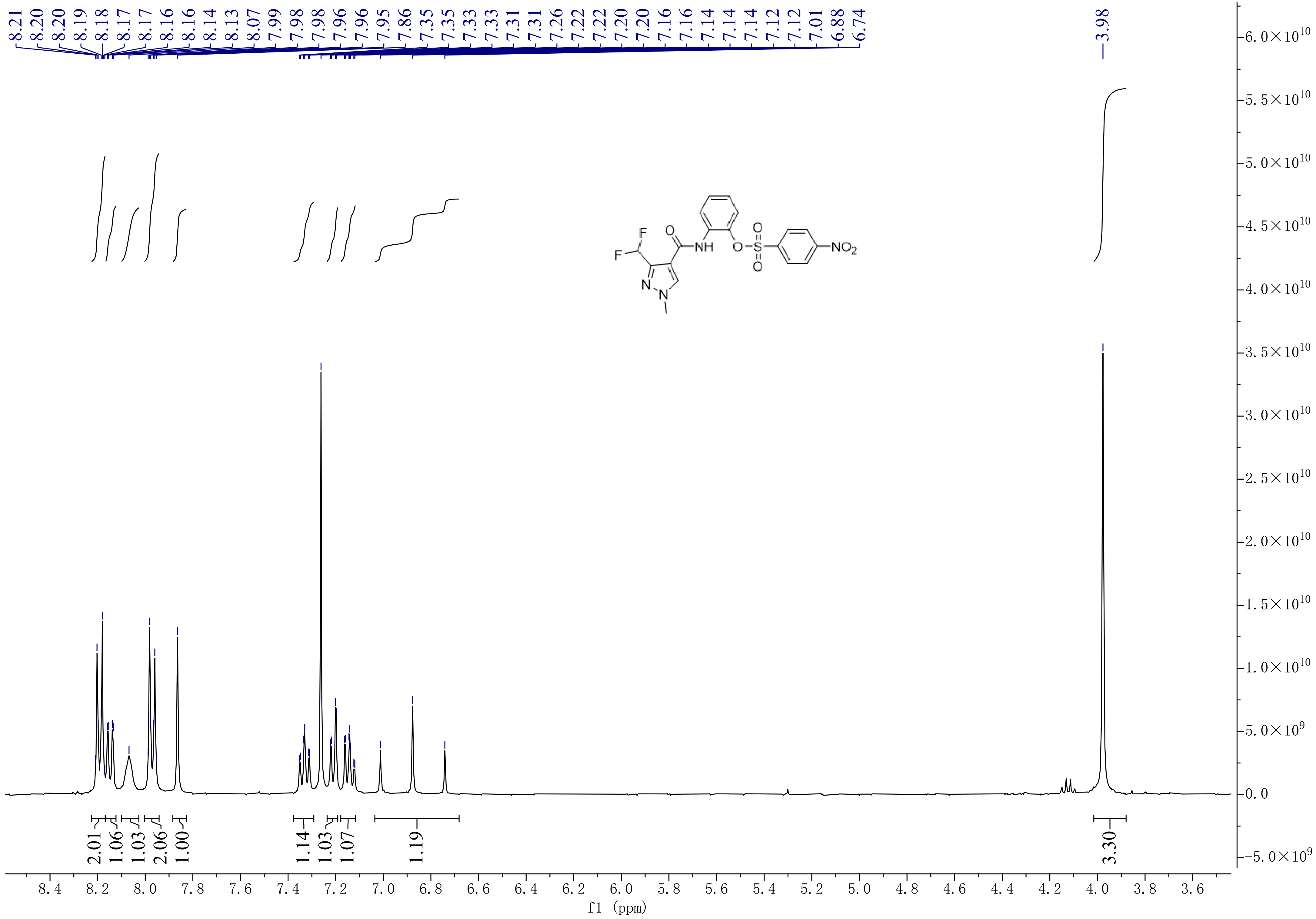
8.21
8.20
8.20
8.19
8.18
8.17
8.17
8.16
8.16
8.14
8.13
8.07
7.99
7.98
7.98
7.96
7.96
7.95
7.86
7.35
7.35
7.33
7.33
7.31
7.31
7.26
7.22
7.22
7.20
7.20
7.16
7.16
7.14
7.14
7.14
7.12
7.12
7.01
6.88
6.74
3.98
2.01
1.06
1.03
2.06
1.00
1.14
1.03
1.07
1.19
3.30
F
F
O
NH
O
S
O
O
N
N
NO_2
f1 (ppm)

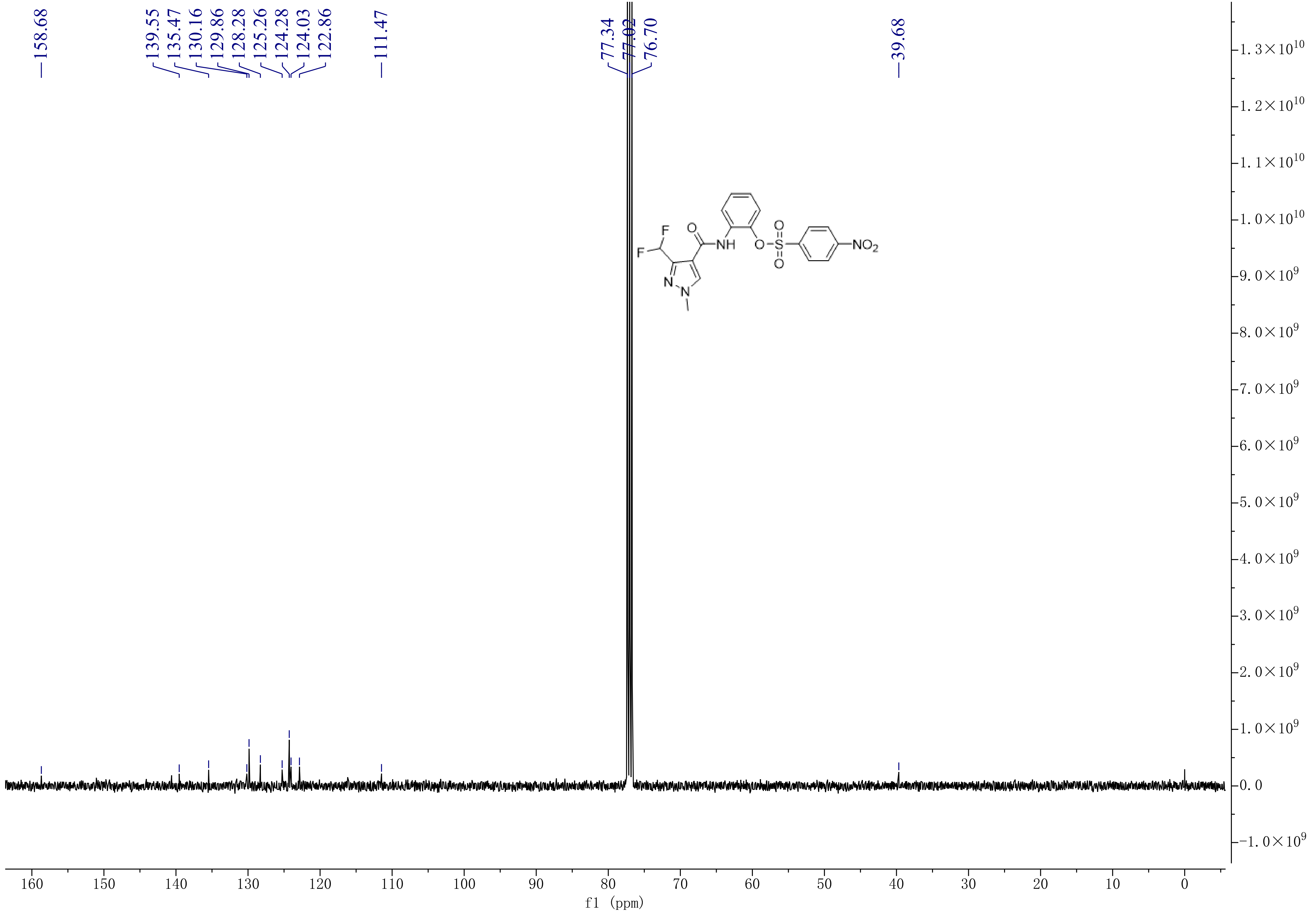
158.68
139.55
135.47
130.16
129.86
128.28
125.26
124.28
124.03
122.86
111.47
77.34
77.02
76.70
39.68
F
F
O
NH
N
N
O
S
O
O
NO2
1.3×10^10
1.2×10^10
1.1×10^10
1.0×10^10
9.0×10^9
8.0×10^9
7.0×10^9
6.0×10^9
5.0×10^9
4.0×10^9
3.0×10^9
2.0×10^9
1.0×10^9
0.0
−1.0×10^9
160 150 140 130 120 110 100 90 80 70 60 50 40 30 20 10 0
f1 (ppm)

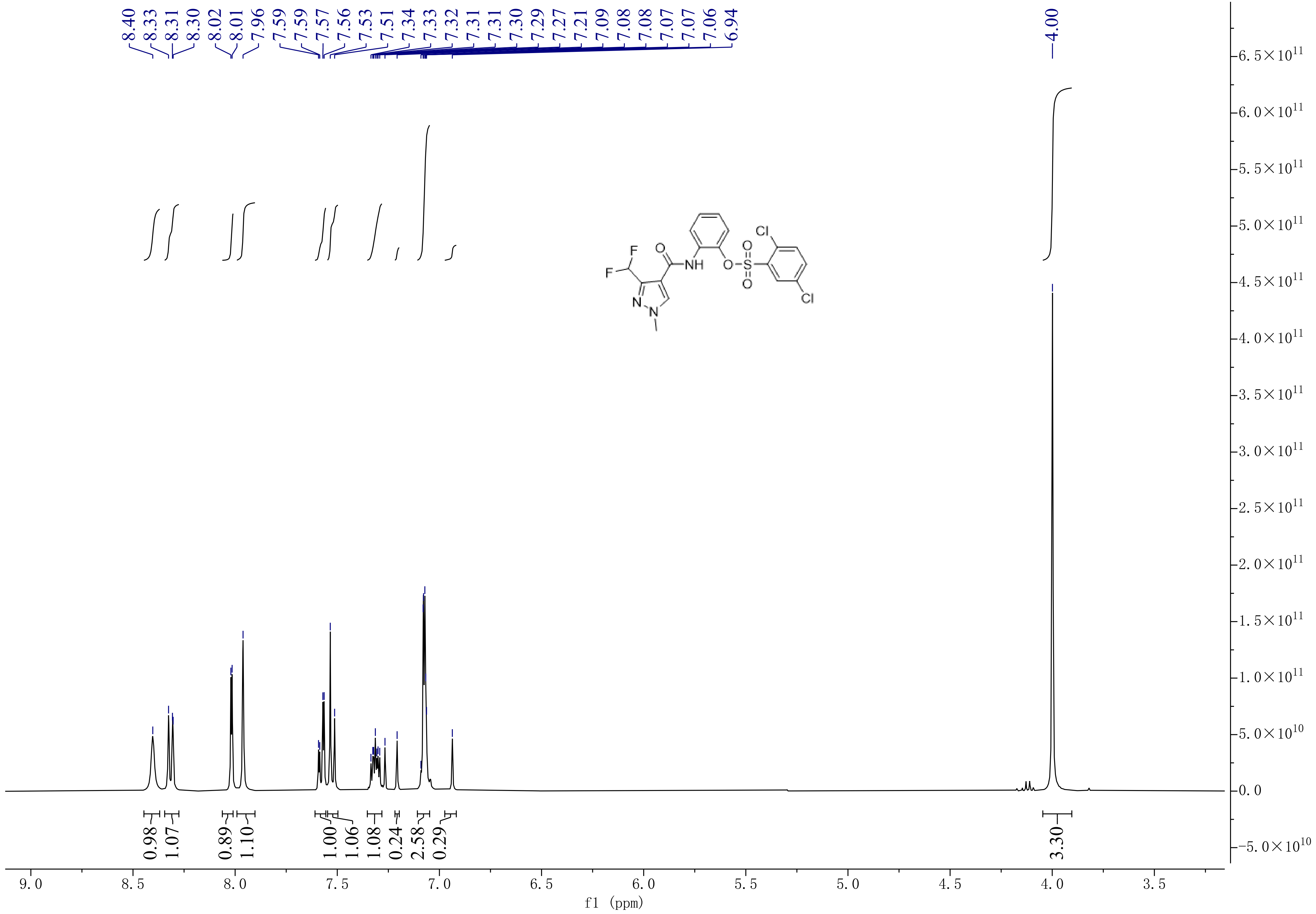
8.40 8.33 8.31 8.30 8.02 8.01 7.96 7.59 7.59 7.57 7.56 7.53 7.51 7.34 7.33 7.32 7.31 7.31 7.30 7.29 7.27 7.21 7.09 7.08 7.08 7.07 7.07 7.06 6.94
4.00
F
F
O
NH
N
N
O
S
O
O
Cl
Cl
0.98 1.07 0.89 1.10 1.00 1.06 1.08 0.24 2.58 0.29
3.30
9.0 8.5 8.0 7.5 7.0 6.5 6.0 5.5 5.0 4.5 4.0 3.5
f1 (ppm)
6.5×10^11 6.0×10^11 5.5×10^11 5.0×10^11 4.5×10^11 4.0×10^11 3.5×10^11 3.0×10^11 2.5×10^11 2.0×10^11 1.5×10^11 1.0×10^11 5.0×10^10 0.0 −5.0×10^10

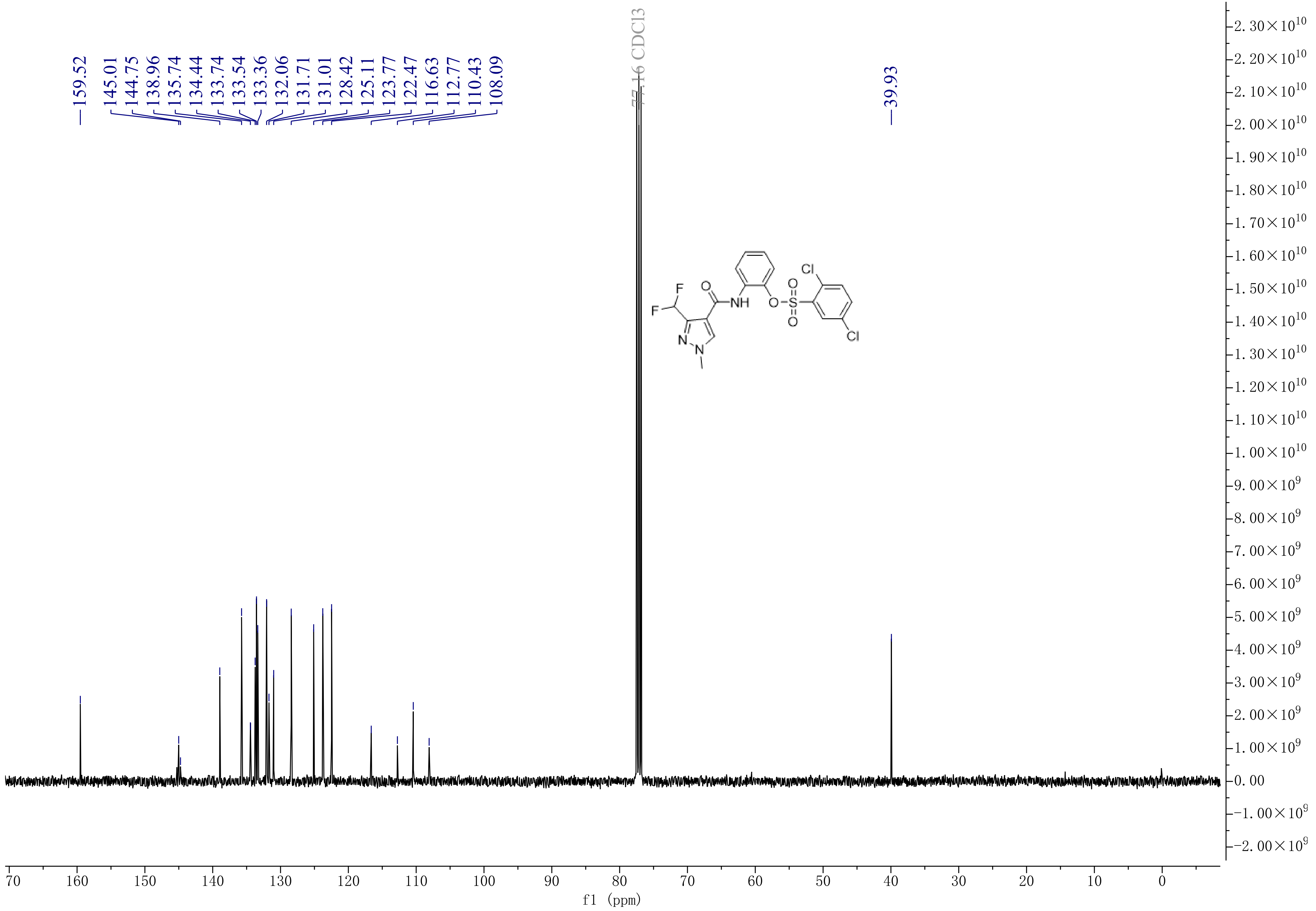
159.52
145.01
144.75
138.96
135.74
134.44
133.74
133.54
133.36
132.06
131.71
131.01
128.42
125.11
123.77
122.47
116.63
112.77
110.43
108.09
77.16 CDCl3
39.93
F
F
O
NH
O
S
O
O
Cl
Cl
N
N
f1 (ppm)

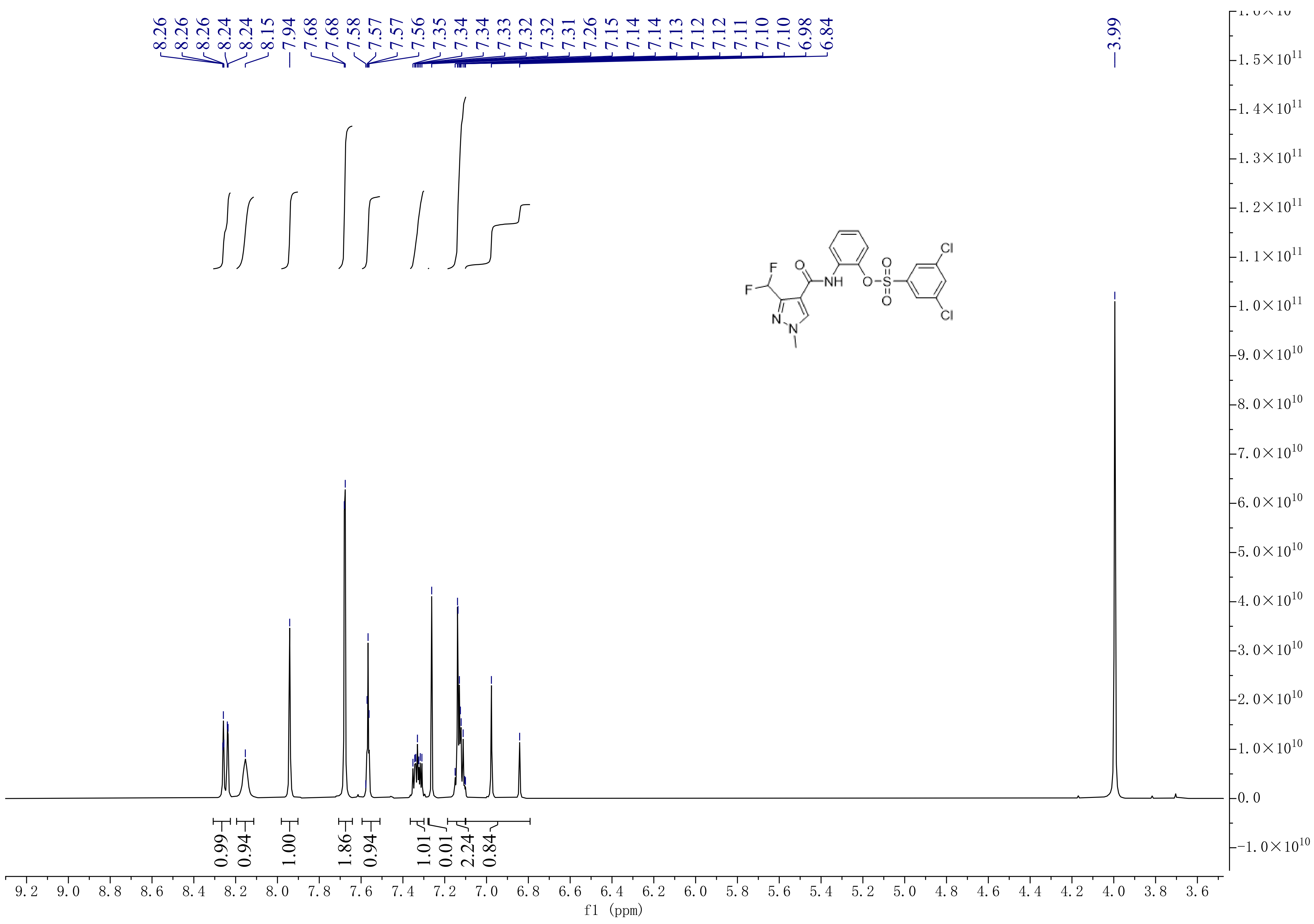
8.26 8.26 8.26 8.24 8.24 8.15 7.94 7.68 7.68 7.58 7.57 7.57 7.56 7.35 7.34 7.34 7.33 7.32 7.32 7.31 7.26 7.15 7.14 7.14 7.13 7.12 7.12 7.11 7.10 7.10 6.98 6.84
3.99
0.99 0.94 1.00 1.86 0.94 1.01 0.01 2.24 0.84
F
F
O
NH
O
S
O
O
Cl
Cl
N
N
f1 (ppm)

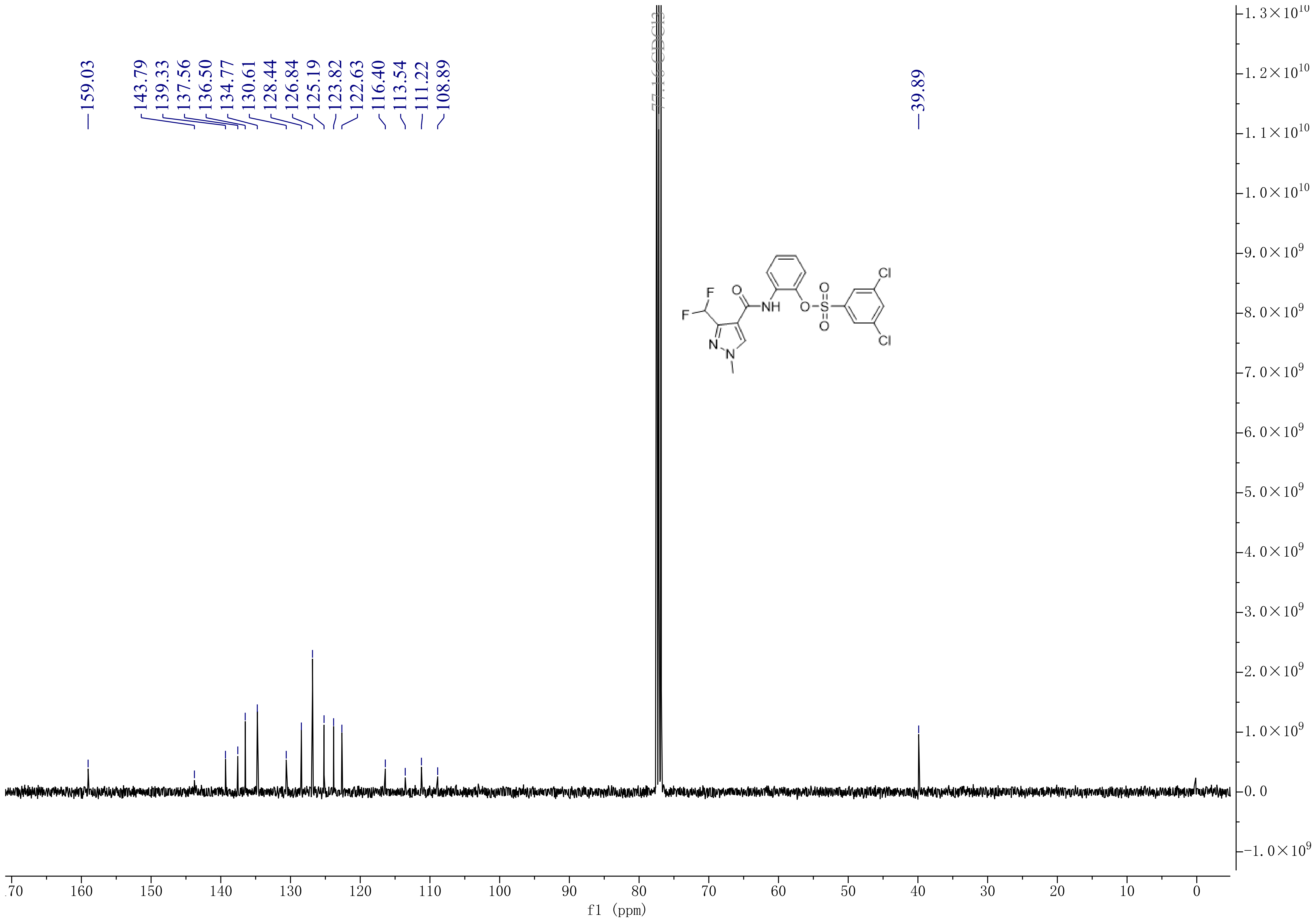

159.03
143.79
139.33
137.56
136.50
134.77
130.61
128.44
126.84
125.19
123.82
122.63
116.40
113.54
111.22
108.89
77.16 CDCl3
39.89
f1 (ppm)

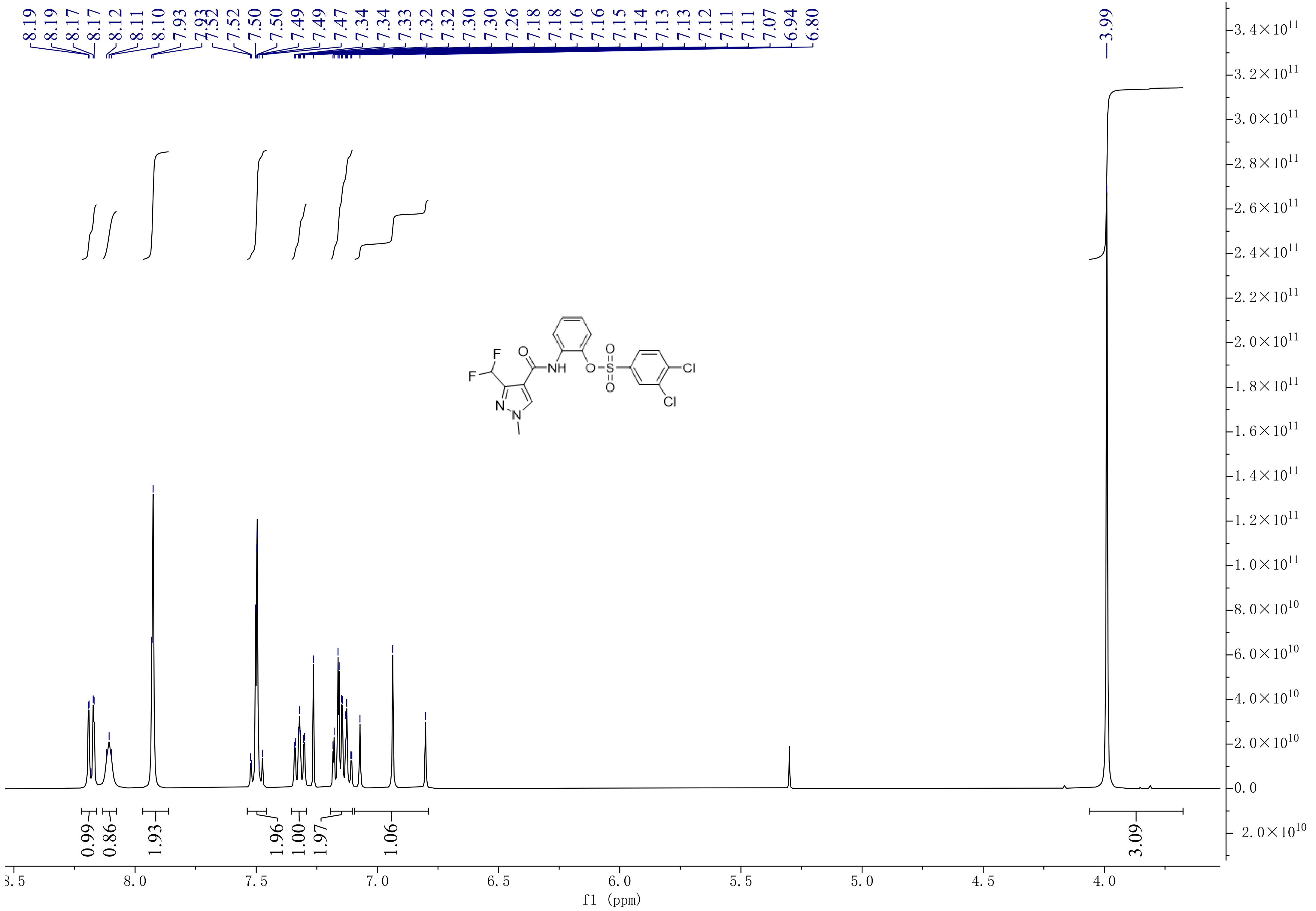
8.19
8.19
8.17
8.17
8.12
8.11
8.10
7.93
7.93
7.52
7.52
7.50
7.50
7.49
7.49
7.47
7.34
7.34
7.33
7.32
7.32
7.30
7.30
7.26
7.18
7.18
7.16
7.16
7.15
7.14
7.13
7.13
7.12
7.11
7.11
7.07
6.94
6.80
3.99
F
F
O
NH
O
S
O
O
Cl
Cl
N
N
0.99
0.86
1.93
1.96
1.00
1.97
1.06
3.09
f1 (ppm)

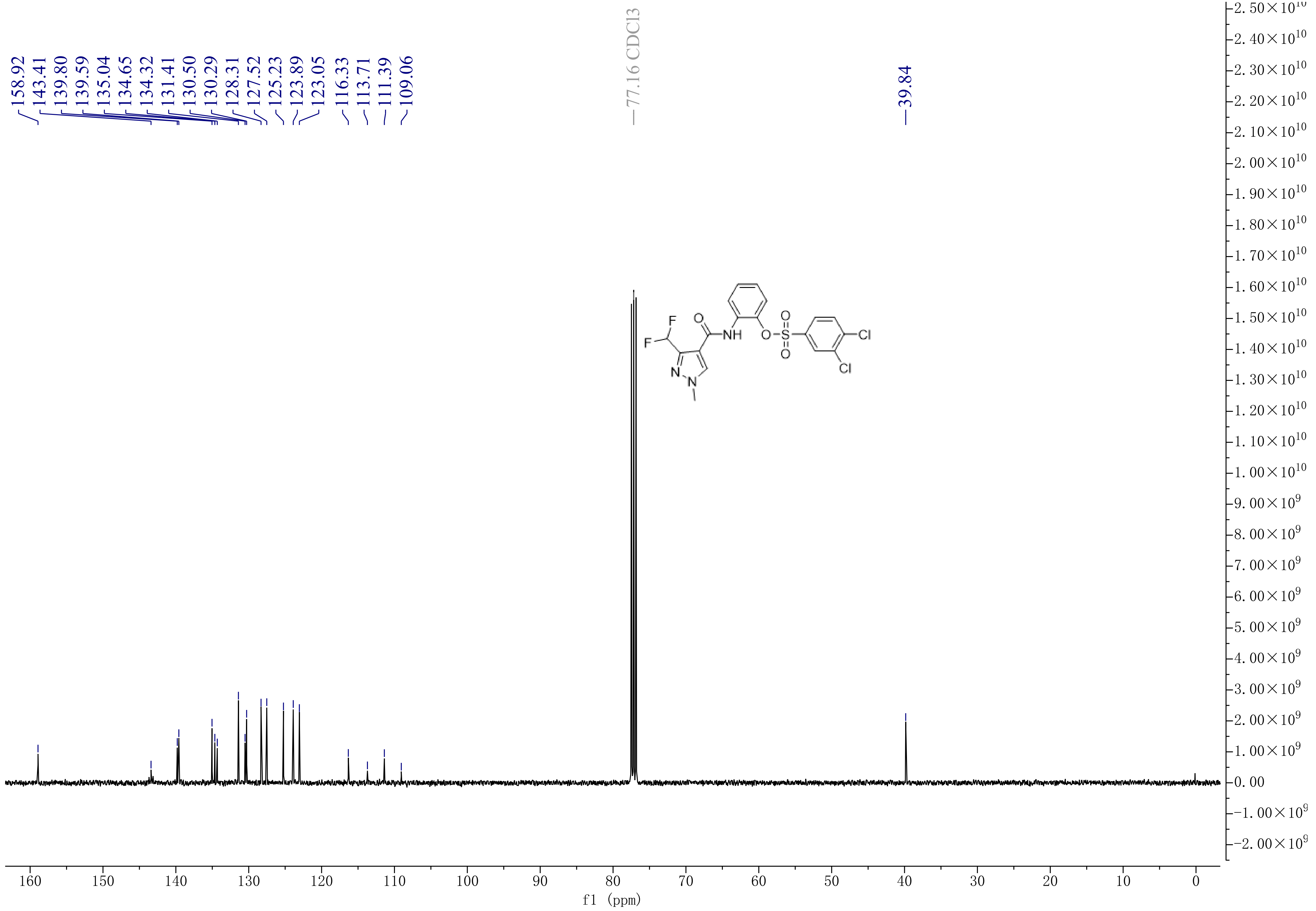
158.92
143.41
139.80
139.59
135.04
134.65
134.32
131.41
130.50
130.29
128.31
127.52
125.23
123.89
123.05
116.33
113.71
111.39
109.06
77.16 CDCl3
39.84
F
F
N
N
O
NH
O
S
O
O
Cl
Cl
160 150 140 130 120 110 100 90 80 70 60 50 40 30 20 10 0
f1 (ppm)
2.50×10^10
2.40×10^10
2.30×10^10
2.20×10^10
2.10×10^10
2.00×10^10
1.90×10^10
1.80×10^10
1.70×10^10
1.60×10^10
1.50×10^10
1.40×10^10
1.30×10^10
1.20×10^10
1.10×10^10
1.00×10^10
9.00×10^9
8.00×10^9
7.00×10^9
6.00×10^9
5.00×10^9
4.00×10^9
3.00×10^9
2.00×10^9
1.00×10^9
0.00
−1.00×10^9
−2.00×10^9

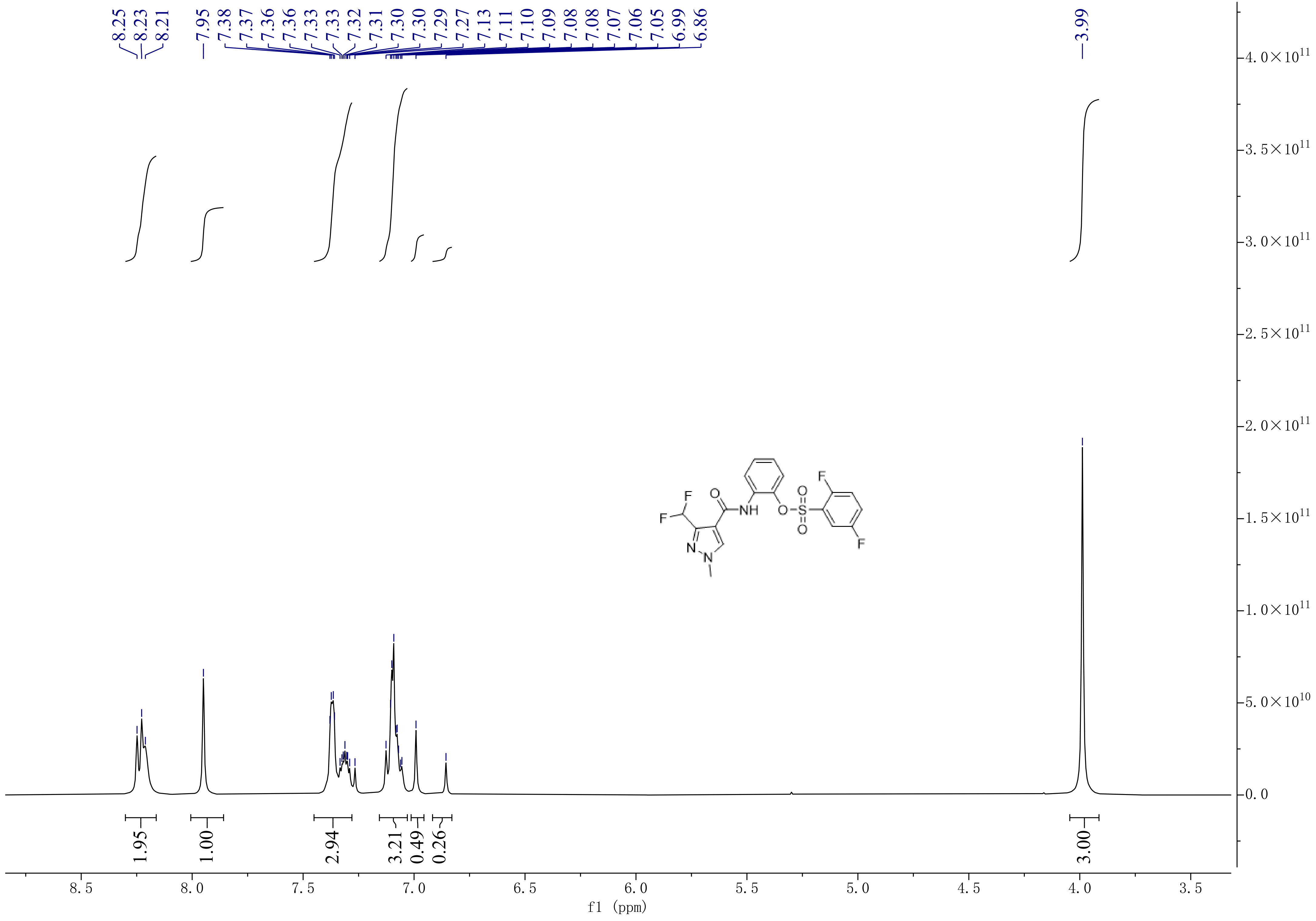
8.25
8.23
8.21
7.95
7.38
7.37
7.36
7.36
7.33
7.33
7.32
7.31
7.30
7.30
7.29
7.27
7.13
7.11
7.10
7.09
7.08
7.08
7.07
7.06
7.05
6.99
6.86
3.99
1.95
1.00
2.94
3.21
0.49
0.26
3.00
F
F
O
NH
O
S
O
O
F
F
N
N
4.0×10^11
3.5×10^11
3.0×10^11
2.5×10^11
2.0×10^11
1.5×10^11
1.0×10^11
5.0×10^10
0.0
8.5
8.0
7.5
7.0
6.5
6.0
5.5
5.0
4.5
4.0
3.5
f1 (ppm)

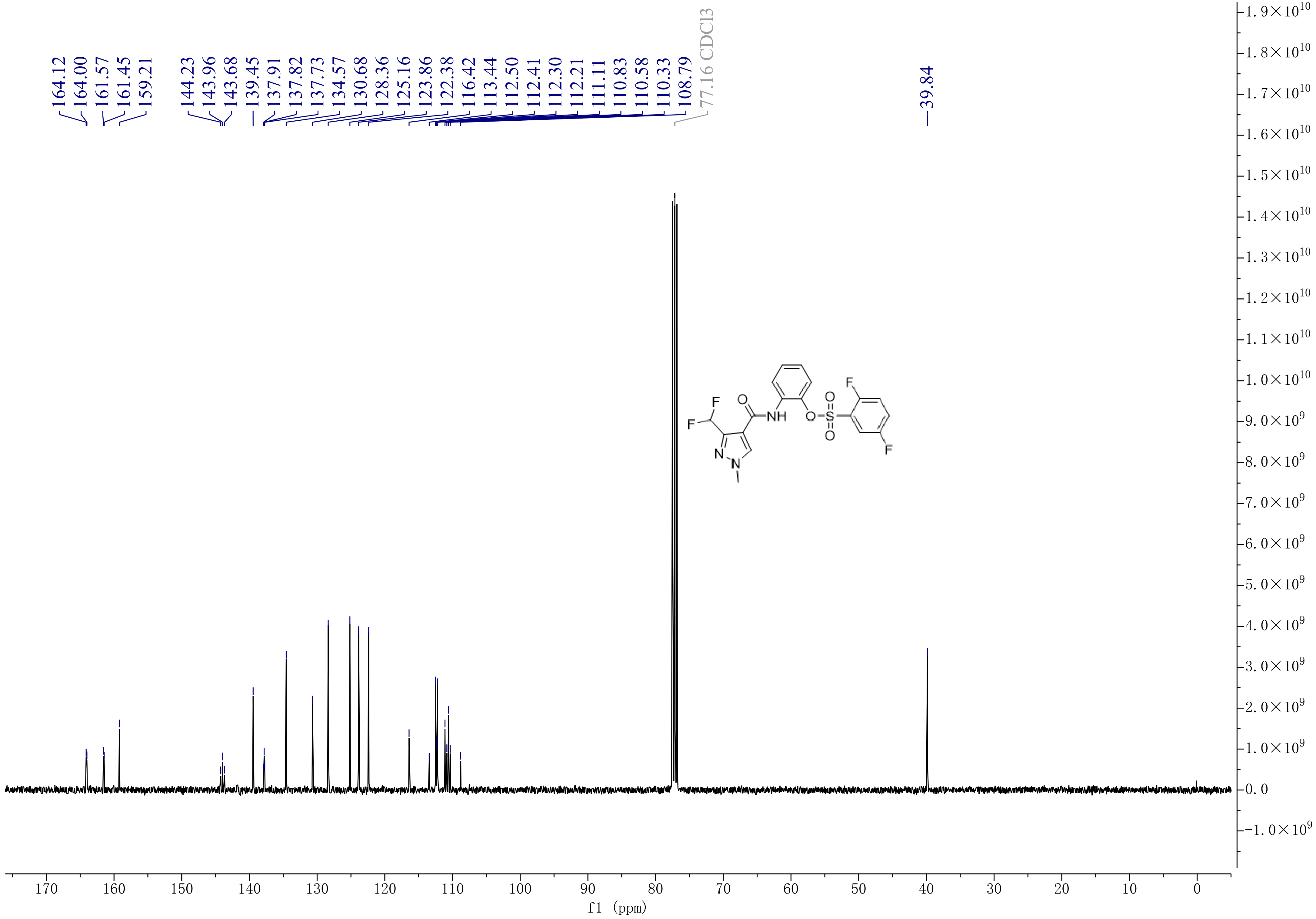

164.12
164.00
161.57
161.45
159.21
144.23
143.96
143.68
139.45
137.91
137.82
137.73
134.57
130.68
128.36
125.16
123.86
122.38
116.42
113.44
112.50
112.41
112.30
112.21
111.11
110.83
110.58
110.33
108.79
77.16 CDCl3
39.84
f1 (ppm)

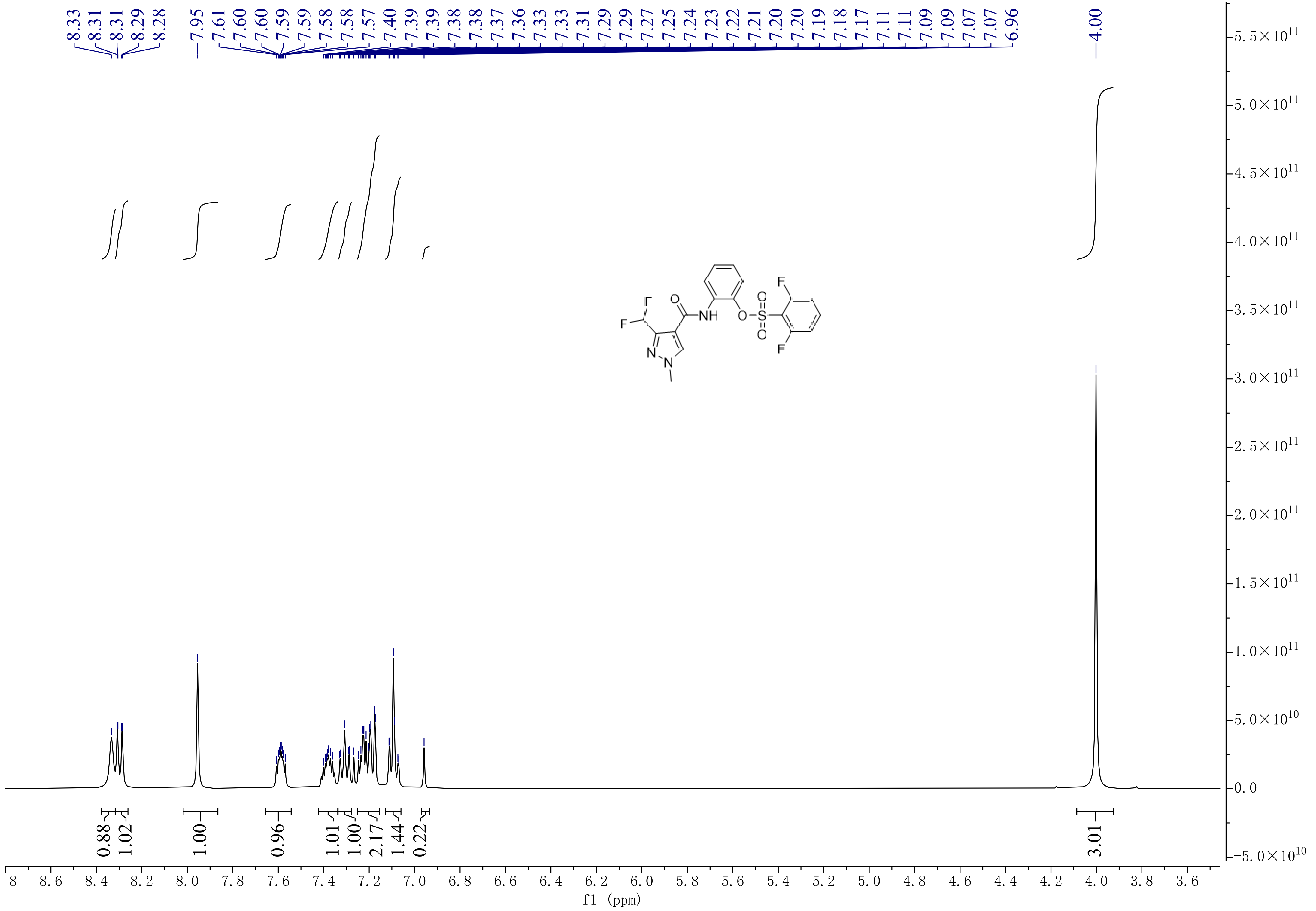

8.33
8.31
8.31
8.29
8.28
7.95
7.61
7.60
7.60
7.59
7.59
7.58
7.58
7.57
7.40
7.39
7.39
7.38
7.38
7.37
7.36
7.33
7.33
7.31
7.29
7.29
7.27
7.25
7.24
7.23
7.22
7.21
7.20
7.20
7.19
7.18
7.17
7.11
7.11
7.09
7.09
7.07
7.07
6.96
4.00
F
F
O
NH
O
S
O
O
F
F
N
N
0.88
1.02
1.00
0.96
1.01
1.00
2.17
1.44
0.22
3.01
f1 (ppm)

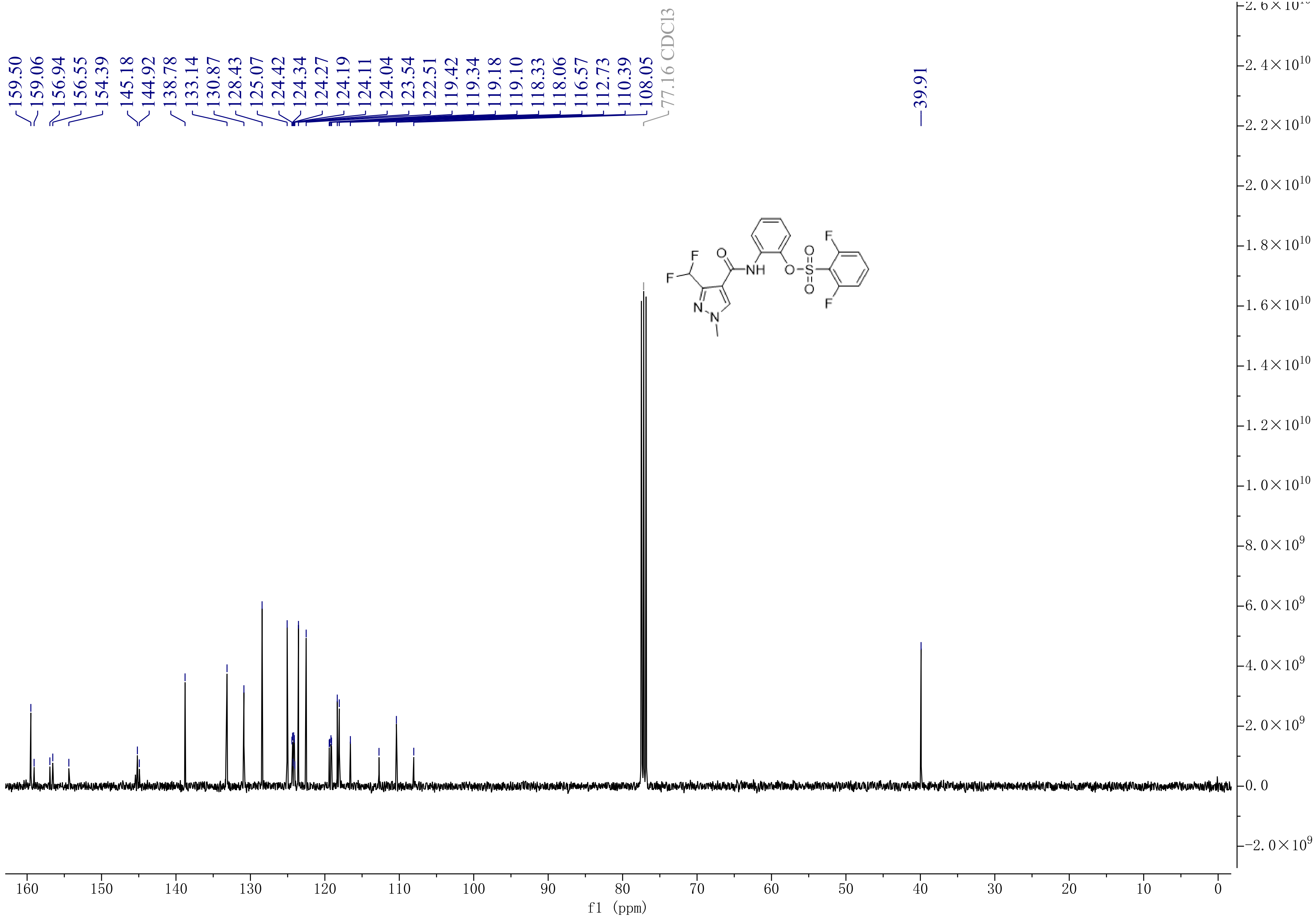
159.50
159.06
156.94
156.55
154.39
145.18
144.92
138.78
133.14
130.87
128.43
125.07
124.42
124.34
124.27
124.19
124.11
124.04
123.54
122.51
119.42
119.34
119.18
119.10
118.33
118.06
116.57
112.73
110.39
108.05
77.16 CDCl3
39.91
f1 (ppm)

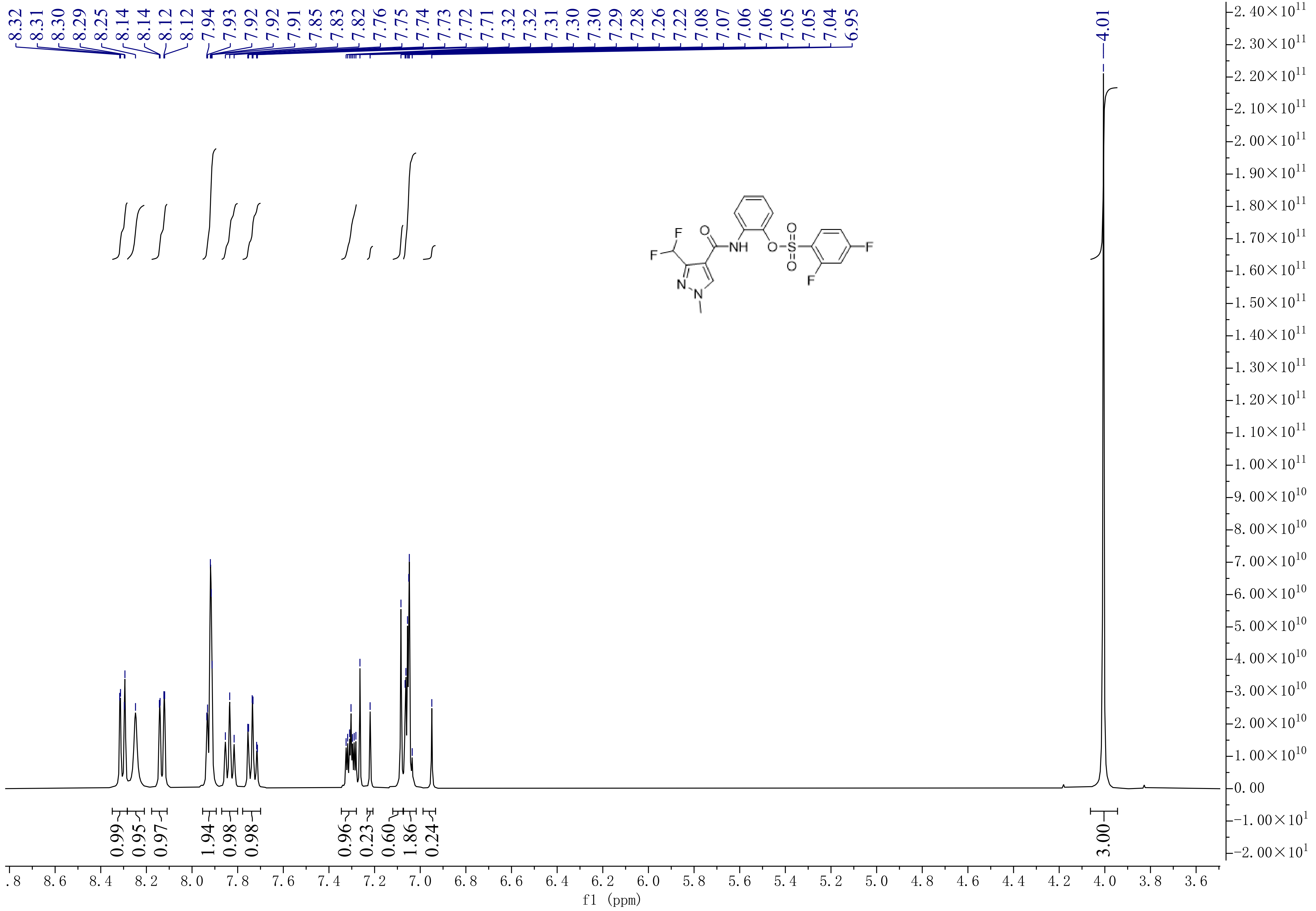
8.32 8.31 8.30 8.29 8.25 8.14 8.14 8.12 8.12 7.94 7.93 7.92 7.92 7.91 7.85 7.83 7.82 7.76 7.75 7.74 7.73 7.72 7.71 7.32 7.32 7.31 7.30 7.30 7.29 7.28 7.26 7.22 7.08 7.07 7.06 7.06 7.05 7.05 7.04 6.95
4.01
0.99 0.95 0.97 1.94 0.98 0.98 0.96 0.23 0.60 1.86 0.24 3.00
f1 (ppm)

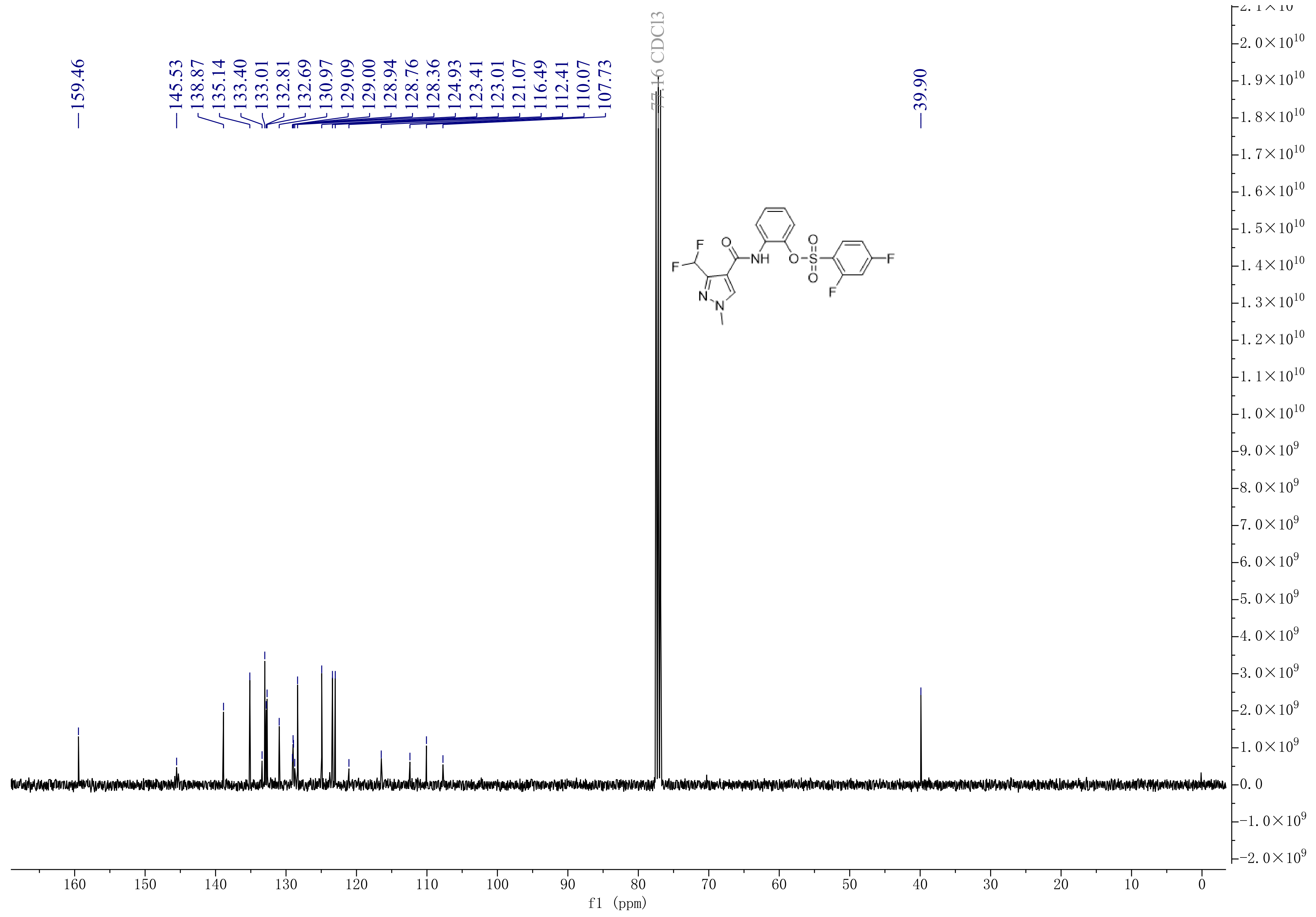
159.46
145.53
138.87
135.14
133.40
133.01
132.81
132.69
130.97
129.09
129.00
128.94
128.76
128.36
124.93
123.41
123.01
121.07
116.49
112.41
110.07
107.73
77.16 CDCl3
39.90
f1 (ppm)

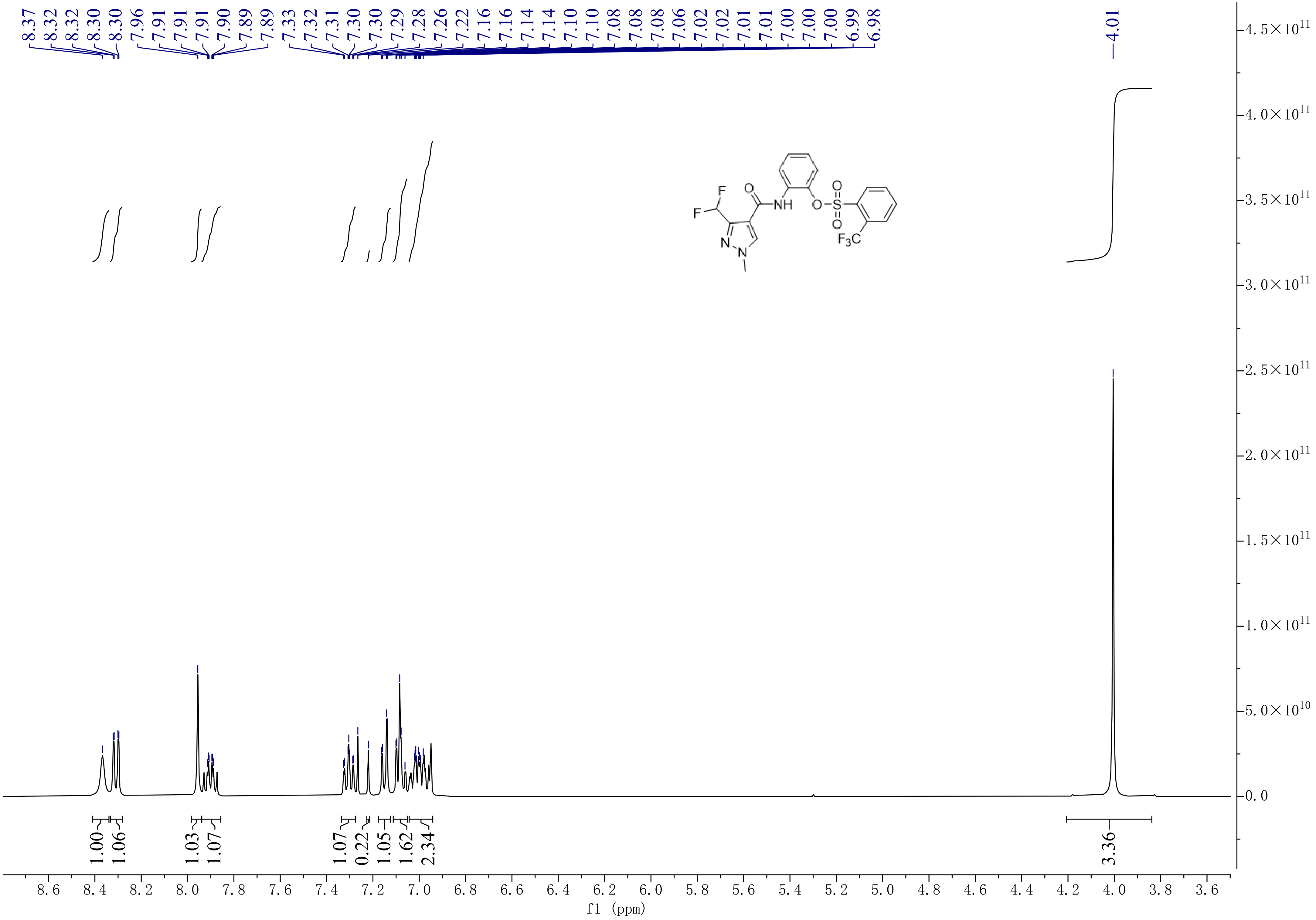
8.37 8.32 8.32 8.30 8.30 7.96 7.91 7.91 7.91 7.90 7.89 7.89 7.33 7.32 7.31 7.30 7.30 7.29 7.28 7.26 7.22 7.16 7.16 7.14 7.14 7.10 7.10 7.08 7.08 7.08 7.06 7.02 7.02 7.01 7.01 7.00 7.00 7.00 6.99 6.98
4.01
F
F
O
NH
O
S
O
O
N
N
F_3C
1.00 1.06 1.03 1.07 1.07 0.22 1.05 1.62 2.34 3.36
f1 (ppm)

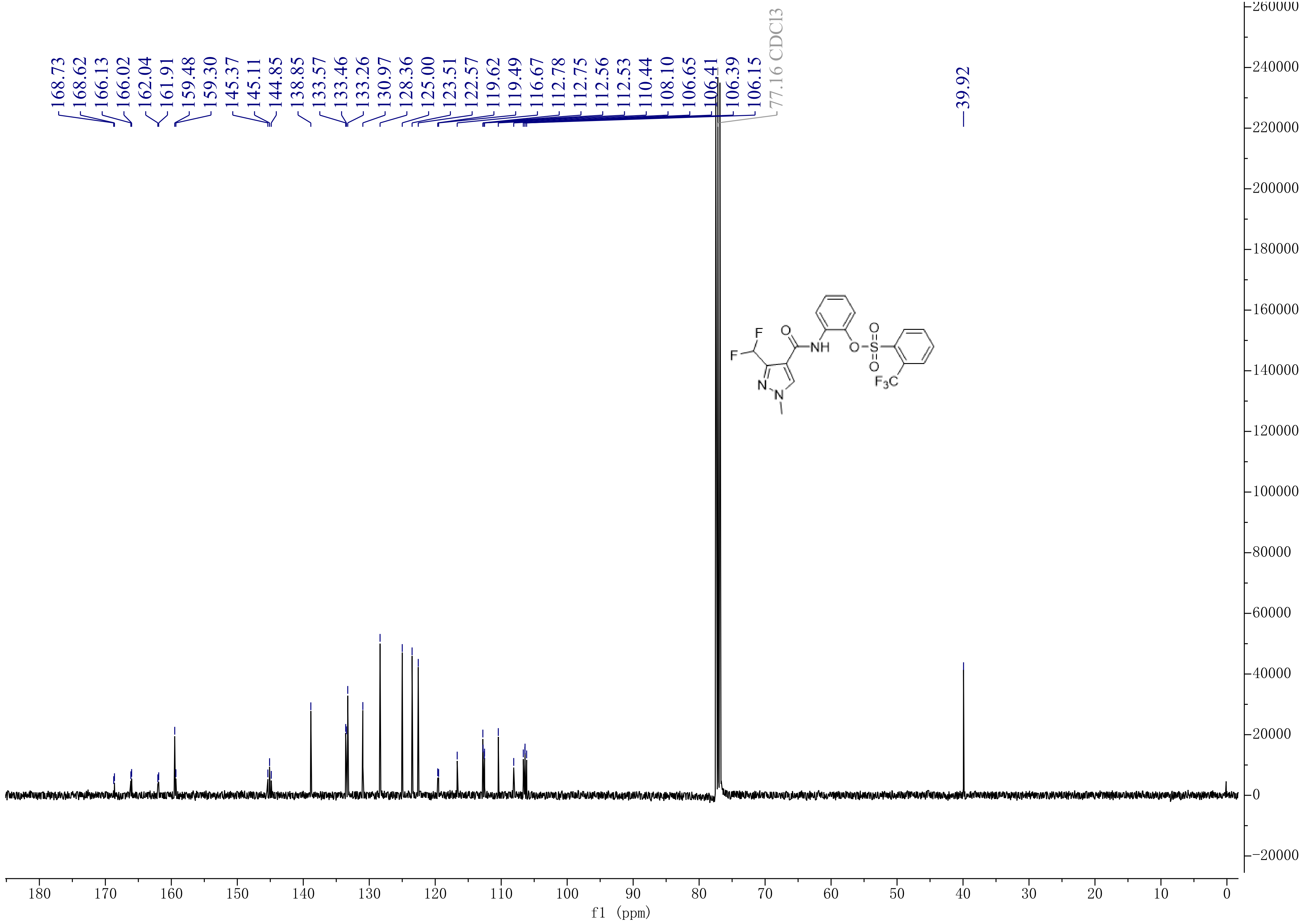
168.73
168.62
166.13
166.02
162.04
161.91
159.48
159.30
145.37
145.11
144.85
138.85
133.57
133.46
133.26
130.97
128.36
125.00
123.51
122.57
119.62
119.49
116.67
112.78
112.75
112.56
112.53
110.44
108.10
106.65
106.41
106.39
106.15
77.16 CDCl3
39.92
F
F
O
NH
O
S
O
O
N
N
F3C
260000
240000
220000
200000
180000
160000
140000
120000
100000
80000
60000
40000
20000
0
-20000
180
170
160
150
140
130
120
110
100
90
80
70
60
50
40
30
20
10
0
f1 (ppm)

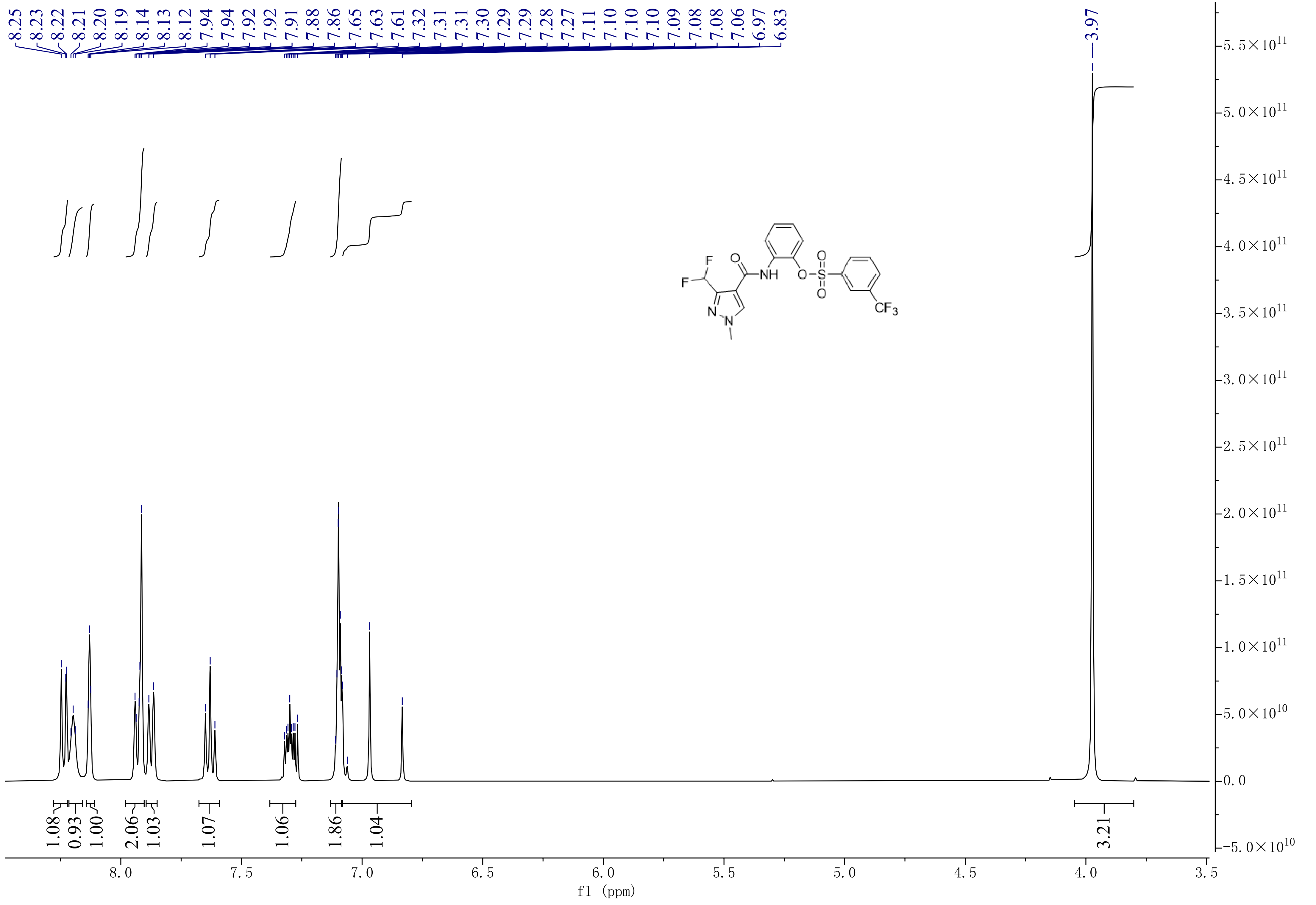
8.25 8.23 8.22 8.21 8.20 8.19 8.14 8.13 8.12 7.94 7.94 7.92 7.92 7.91 7.88 7.86 7.65 7.63 7.61 7.32 7.31 7.31 7.30 7.29 7.29 7.28 7.27 7.11 7.10 7.10 7.10 7.09 7.08 7.08 7.06 6.97 6.83
3.97
1.08 0.93 1.00 2.06 1.03 1.07 1.06 1.86 1.04 3.21
F
F
O
NH
O
S
O
O
N
N
CF3
f1 (ppm)

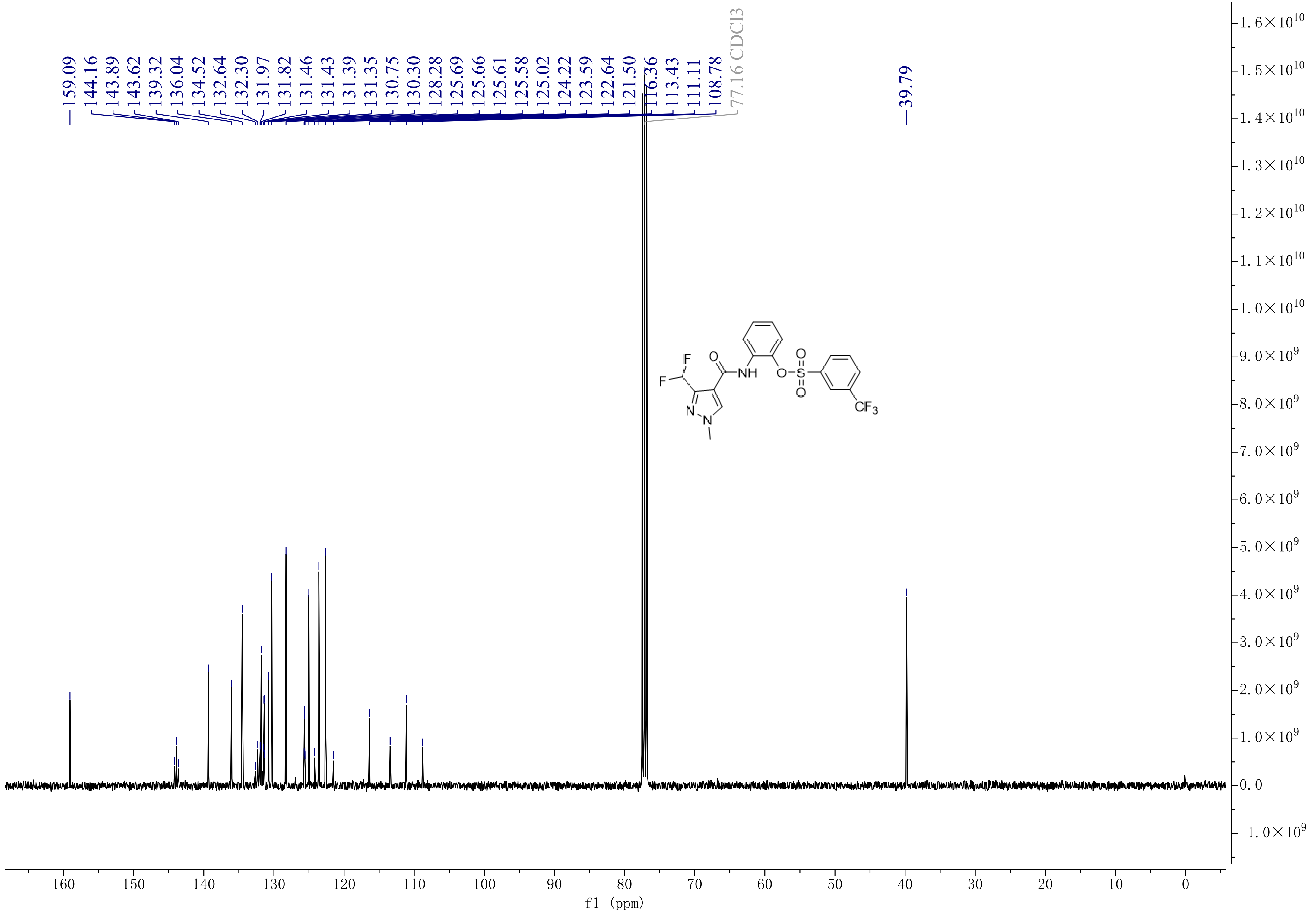

159.09
144.16
143.89
143.62
139.32
136.04
134.52
132.64
132.30
131.97
131.82
131.46
131.43
131.39
131.35
130.75
130.30
128.28
125.69
125.66
125.61
125.58
125.02
124.22
123.59
122.64
121.50
116.36
113.43
111.11
108.78
77.16 CDCl3
39.79
F
F
O
NH
O
S
O
O
N
N
CF3
1.6×10^10
1.5×10^10
1.4×10^10
1.3×10^10
1.2×10^10
1.1×10^10
1.0×10^10
9.0×10^9
8.0×10^9
7.0×10^9
6.0×10^9
5.0×10^9
4.0×10^9
3.0×10^9
2.0×10^9
1.0×10^9
0.0
-1.0×10^9
160
150
140
130
120
110
100
90
80
70
60
50
40
30
20
10
0
f1 (ppm)

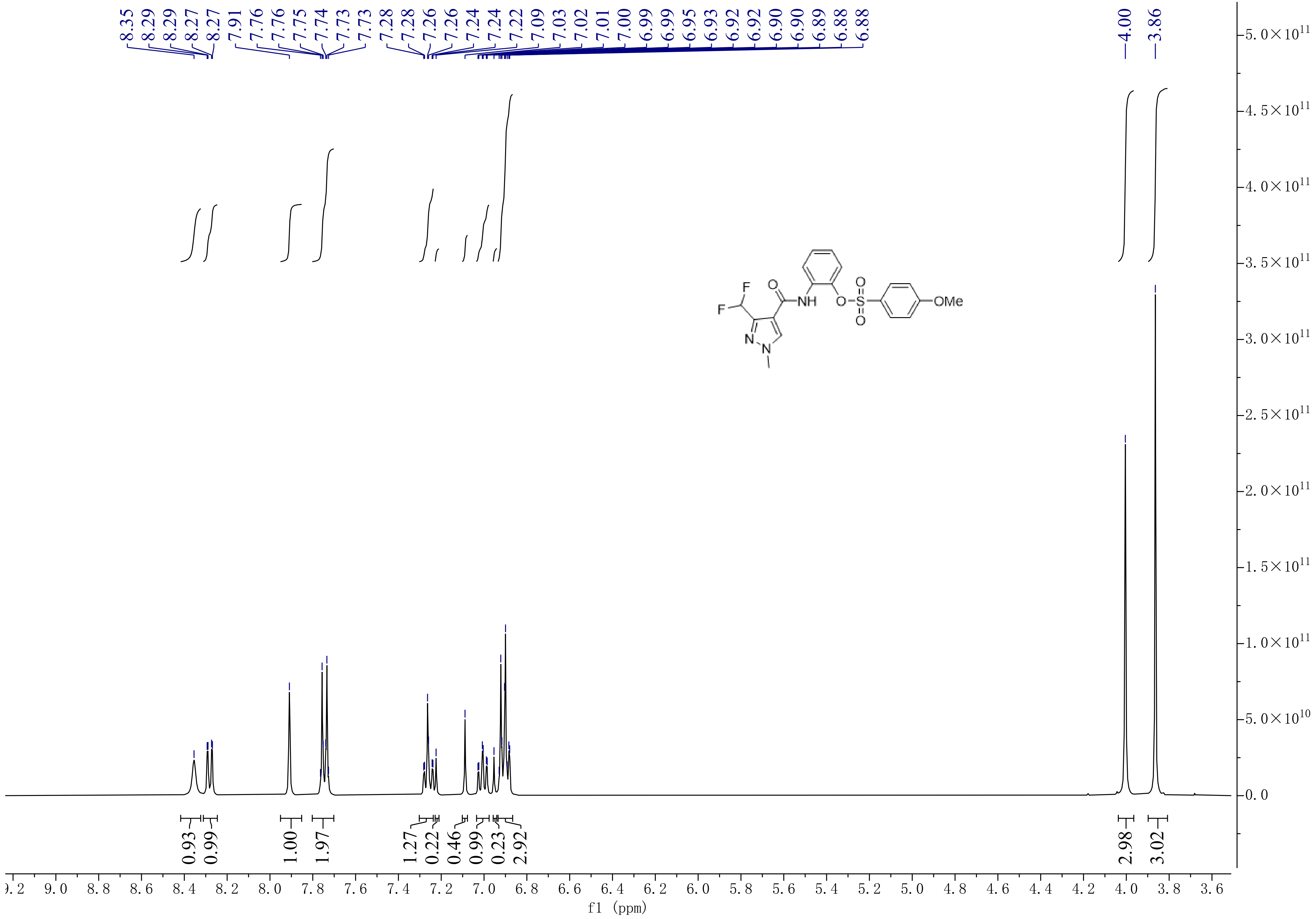
8.35
8.29
8.29
8.27
8.27
7.91
7.76
7.76
7.75
7.74
7.73
7.73
7.28
7.28
7.26
7.26
7.24
7.24
7.22
7.09
7.03
7.02
7.01
7.00
6.99
6.99
6.95
6.93
6.92
6.92
6.90
6.90
6.89
6.88
6.88
4.00
3.86
F
F
O
NH
O
S
O
O
OMe
N
N
0.93
0.99
1.00
1.97
1.27
0.22
0.46
0.99
0.23
2.92
2.98
3.02
f1 (ppm)

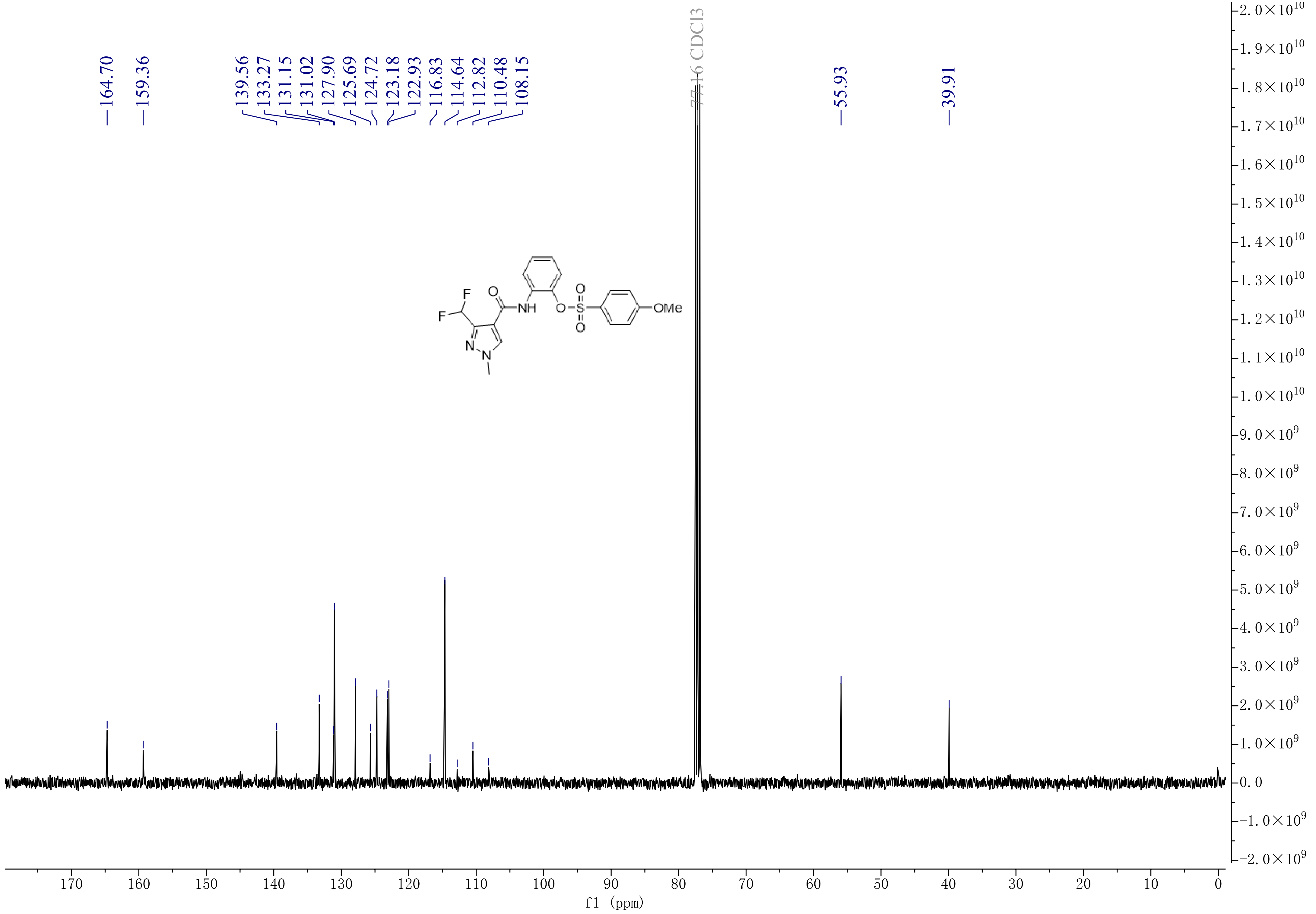

164.70
159.36
139.56
133.27
131.15
131.02
127.90
125.69
124.72
123.18
122.93
116.83
114.64
112.82
110.48
108.15
77.16 CDCl3
55.93
39.91
F
F
O
NH
O
S
O
O
OMe
N
N
f1 (ppm)

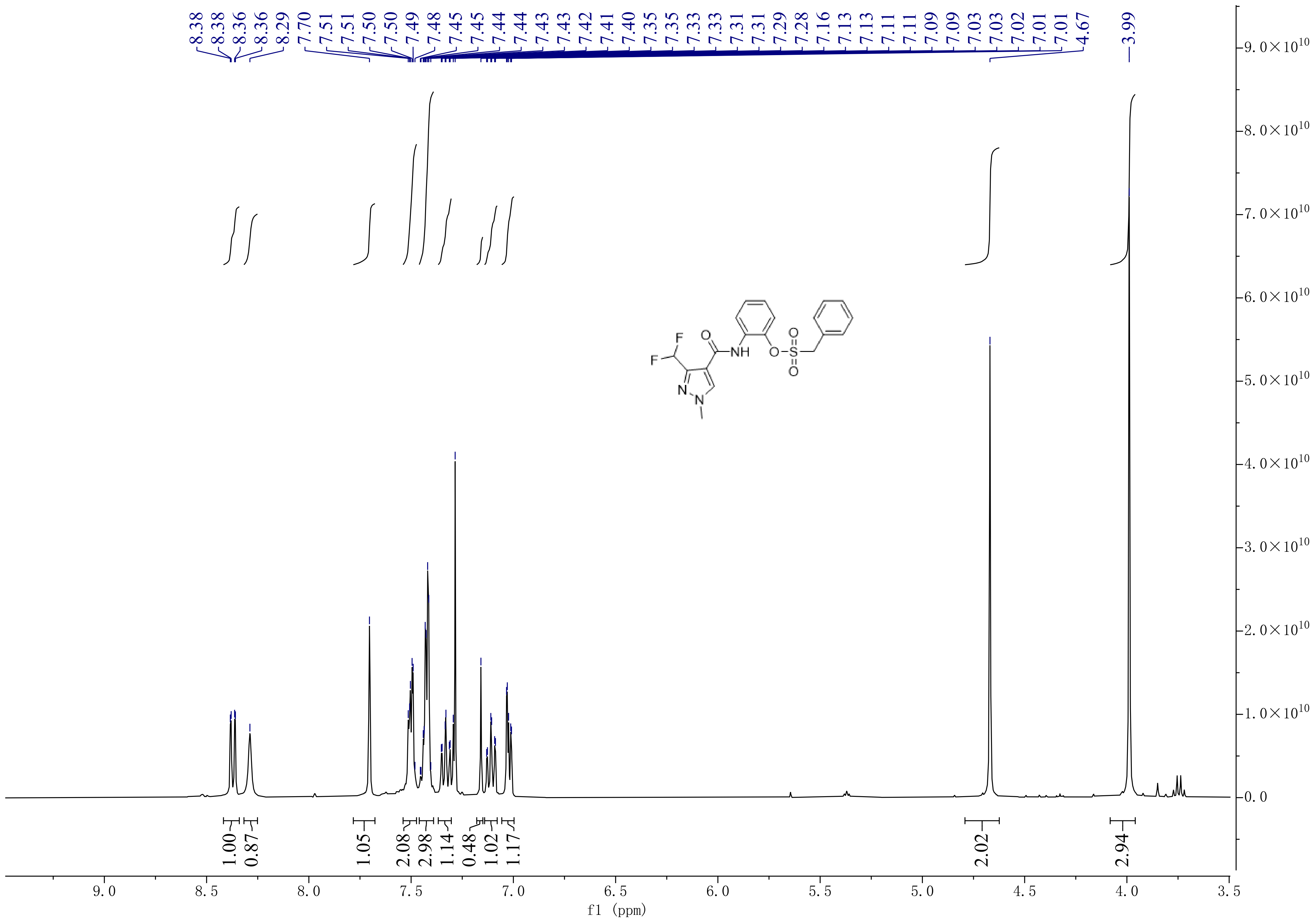

8.38
8.38
8.36
8.36
8.29
7.70
7.51
7.51
7.50
7.50
7.49
7.48
7.45
7.45
7.44
7.44
7.43
7.43
7.42
7.41
7.40
7.35
7.35
7.33
7.33
7.31
7.31
7.29
7.28
7.16
7.13
7.13
7.11
7.11
7.09
7.09
7.03
7.03
7.02
7.01
7.01
4.67
3.99
1.00
0.87
1.05
2.08
2.98
1.14
0.48
1.02
1.17
2.02
2.94
F
F
O
NH
O
S
O
O
N
N
9.0×10^10
8.0×10^10
7.0×10^10
6.0×10^10
5.0×10^10
4.0×10^10
3.0×10^10
2.0×10^10
1.0×10^10
0.0
9.0
8.5
8.0
7.5
7.0
6.5
6.0
5.5
5.0
4.5
4.0
3.5
f1 (ppm)

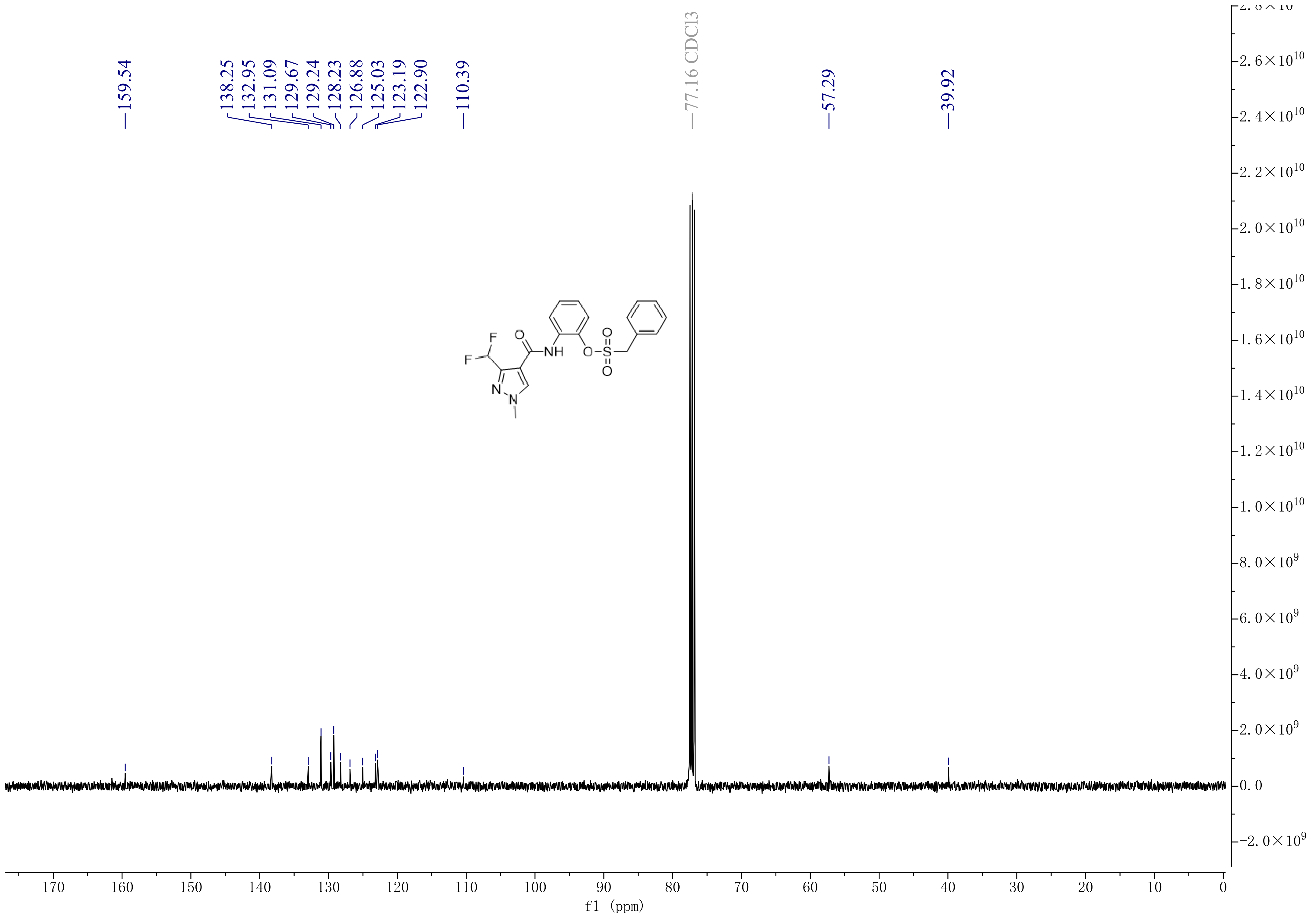

159.54
138.25
132.95
131.09
129.67
129.24
128.23
126.88
125.03
123.19
122.90
110.39
77.16 CDCl3
57.29
39.92
F
F
O
NH
O
S
O
O
N
N
f1 (ppm)

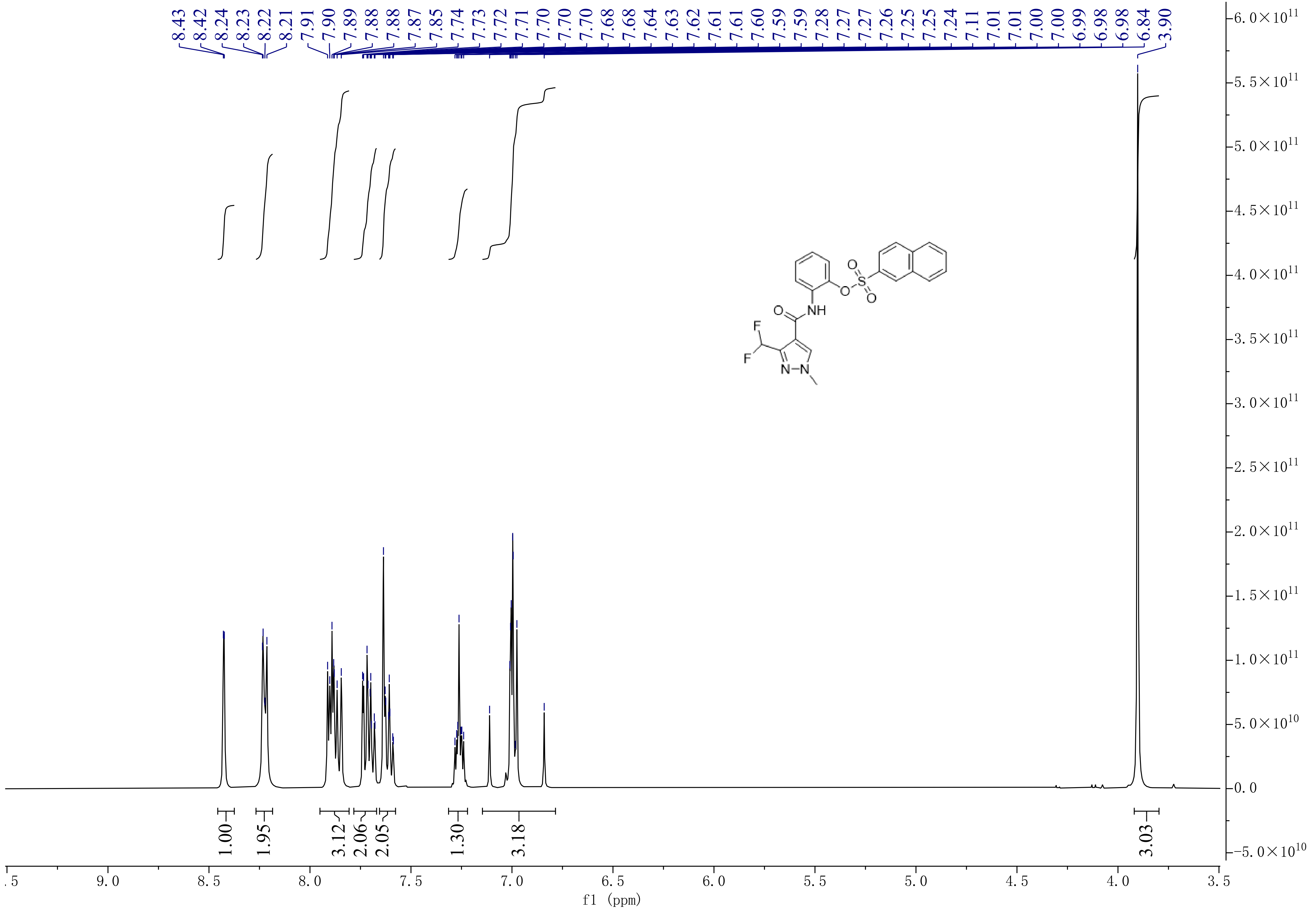

8.43
8.42
8.24
8.23
8.22
8.21
7.91
7.90
7.89
7.88
7.88
7.87
7.85
7.74
7.73
7.72
7.71
7.70
7.70
7.70
7.68
7.68
7.64
7.63
7.62
7.61
7.61
7.60
7.59
7.59
7.28
7.27
7.27
7.26
7.25
7.25
7.24
7.11
7.01
7.01
7.00
7.00
6.99
6.98
6.98
6.84
3.90
1.00
1.95
3.12
2.06
2.05
1.30
3.18
3.03
O
NH
O
S
O
O
F
F
N
N
f1 (ppm)
.5
9.0
8.5
8.0
7.5
7.0
6.5
6.0
5.5
5.0
4.5
4.0
3.5
6.0×10^11
5.5×10^11
5.0×10^11
4.5×10^11
4.0×10^11
3.5×10^11
3.0×10^11
2.5×10^11
2.0×10^11
1.5×10^11
1.0×10^11
5.0×10^10
0.0
−5.0×10^10

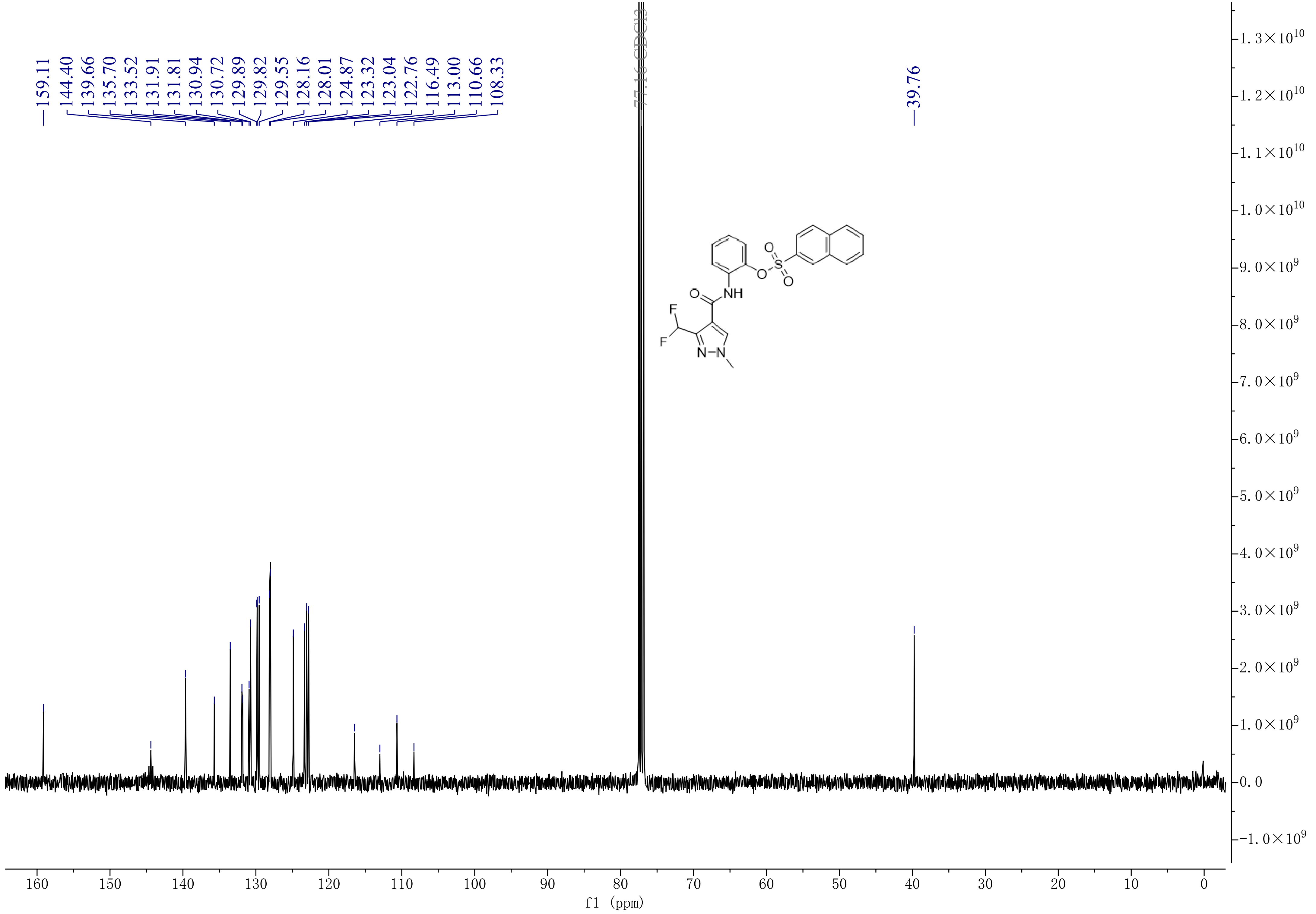
159.11
144.40
139.66
135.70
133.52
131.91
131.81
130.94
130.72
129.89
129.82
129.55
128.16
128.01
124.87
123.32
123.04
122.76
116.49
113.00
110.66
108.33
77.16 CDCl3
39.76
f1 (ppm)

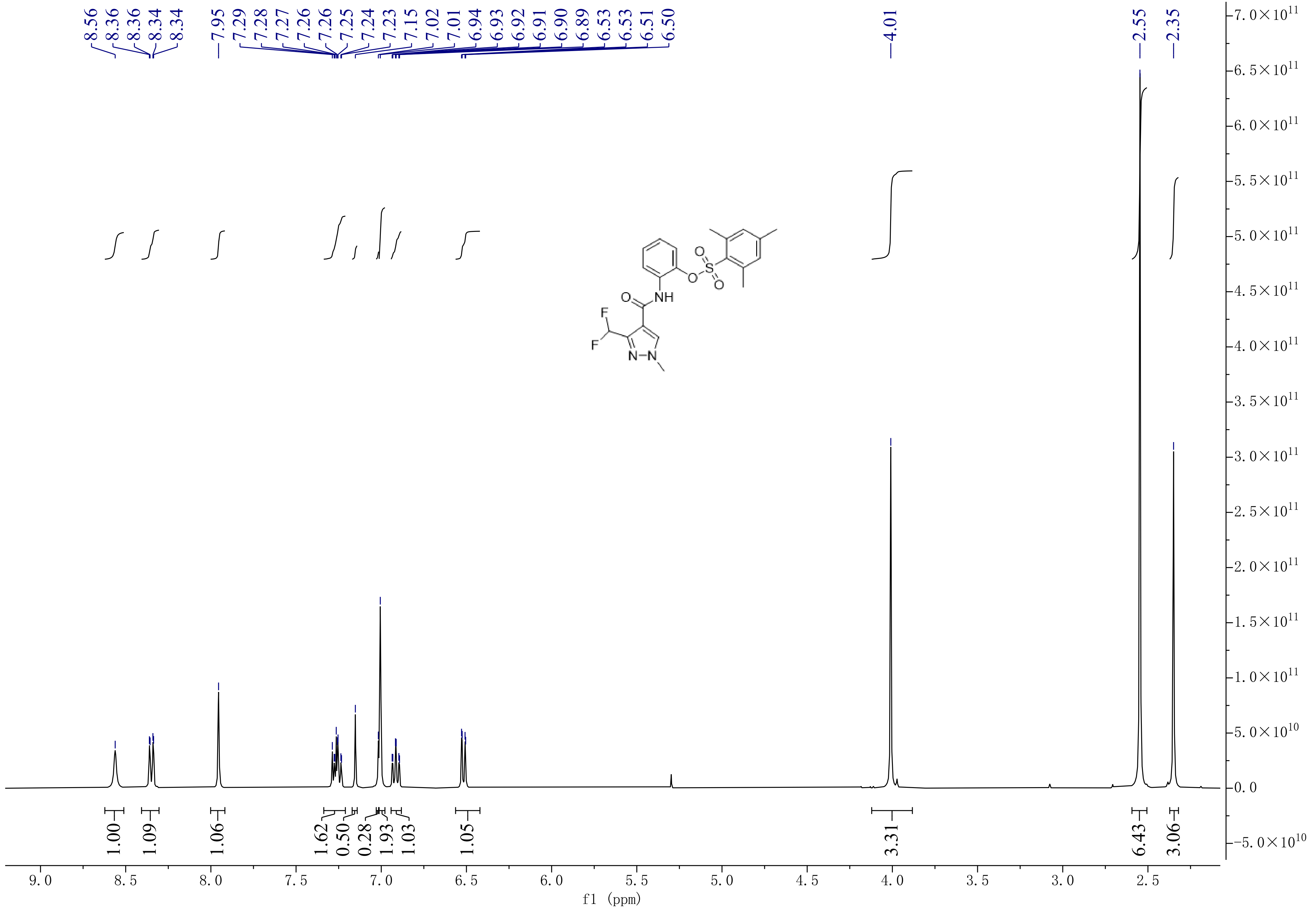

8.56 8.36 8.36 8.34 8.34 7.95 7.29 7.28 7.27 7.26 7.26 7.25 7.24 7.23 7.15 7.02 7.01 6.94 6.93 6.92 6.91 6.90 6.89 6.53 6.53 6.51 6.50
4.01
2.55 2.35
1.00 1.09 1.06 1.62 0.50 0.28 1.93 1.03 1.05 3.31 6.43 3.06
9.0 8.5 8.0 7.5 7.0 6.5 6.0 5.5 5.0 4.5 4.0 3.5 3.0 2.5
f1 (ppm)
7.0×10^11 6.5×10^11 6.0×10^11 5.5×10^11 5.0×10^11 4.5×10^11 4.0×10^11 3.5×10^11 3.0×10^11 2.5×10^11 2.0×10^11 1.5×10^11 1.0×10^11 5.0×10^10 0.0 -5.0×10^10
O
S
O
O
NH
O
F
F
N
N

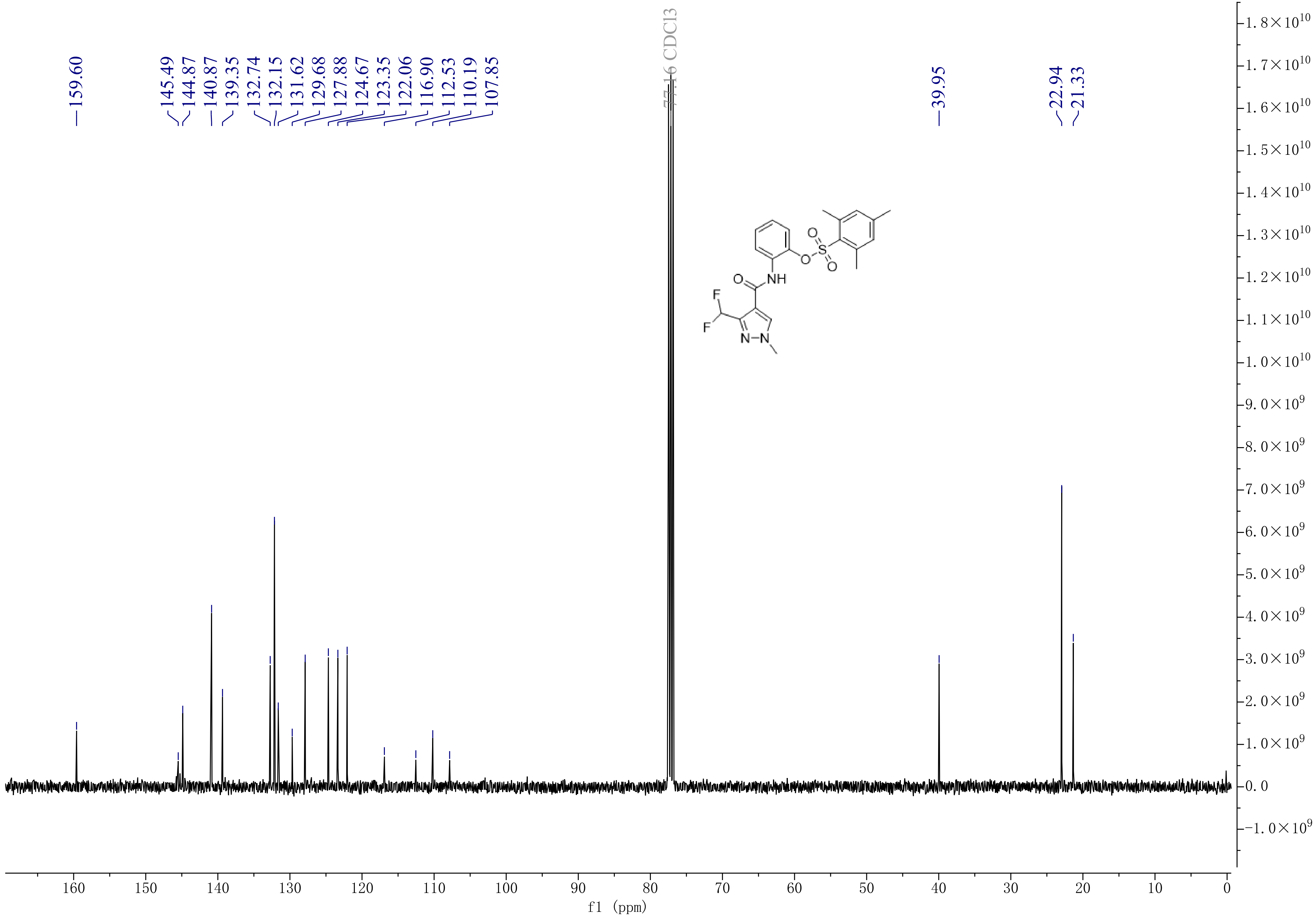
159.60
145.49
144.87
140.87
139.35
132.74
132.15
131.62
129.68
127.88
124.67
123.35
122.06
116.90
112.53
110.19
107.85
77.16 CDCl3
39.95
22.94
21.33
F
F
N
N
O
NH
O
S
O
O
f1 (ppm)
160
150
140
130
120
110
100
90
80
70
60
50
40
30
20
10
0
1.8×10^10
1.7×10^10
1.6×10^10
1.5×10^10
1.4×10^10
1.3×10^10
1.2×10^10
1.1×10^10
1.0×10^10
9.0×10^9
8.0×10^9
7.0×10^9
6.0×10^9
5.0×10^9
4.0×10^9
3.0×10^9
2.0×10^9
1.0×10^9
0.0
-1.0×10^9

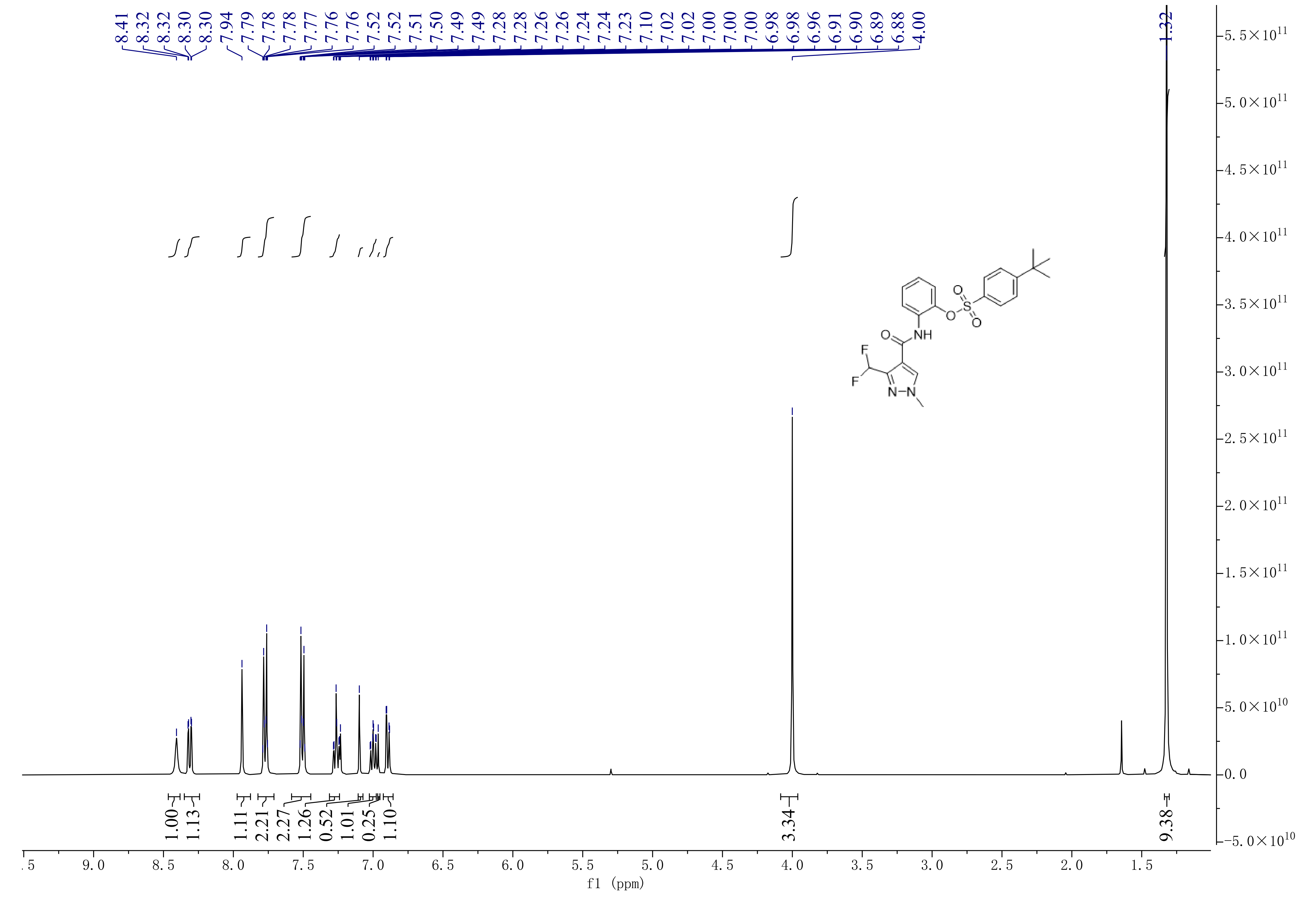
8.41 8.32 8.32 8.30 8.30 7.94 7.79 7.78 7.78 7.77 7.76 7.76 7.52 7.52 7.51 7.50 7.49 7.49 7.28 7.28 7.26 7.26 7.24 7.24 7.23 7.10 7.02 7.02 7.00 7.00 7.00 6.98 6.98 6.96 6.91 6.90 6.89 6.88 4.00
1.32
1.00 1.13 1.11 2.21 2.27 1.26 0.52 1.01 0.25 1.10
3.34
9.38
F
F
N
N
O
NH
O
S
O
O
f1 (ppm)

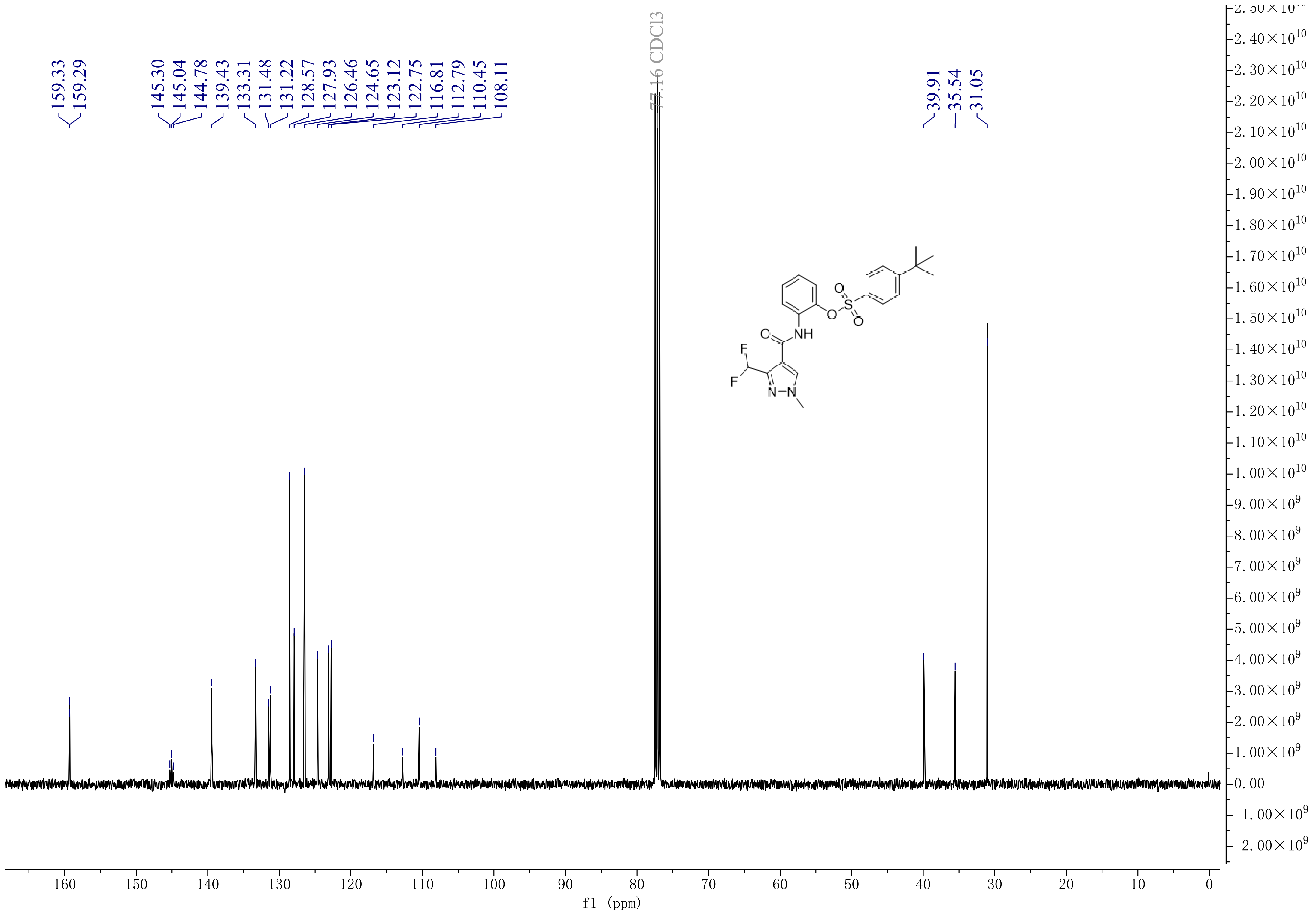

159.33
159.29
145.30
145.04
144.78
139.43
133.31
131.48
131.22
128.57
127.93
126.46
124.65
123.12
122.75
116.81
112.79
110.45
108.11
77.16 CDCl3
39.91
35.54
31.05
f1 (ppm)

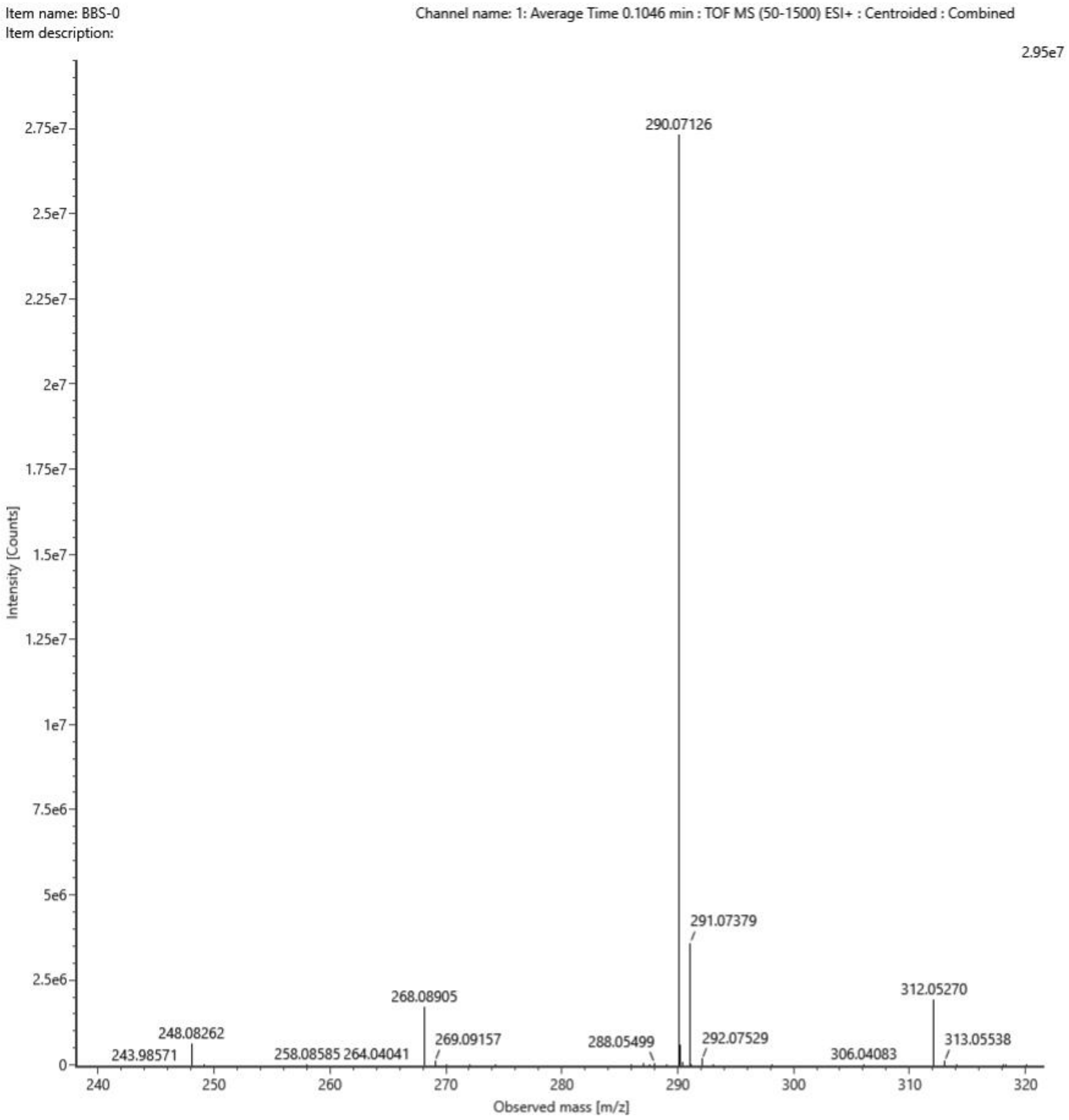

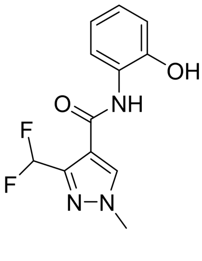

6

Add:Na+

Composition	i-FIT Confidence (%)	Predicted m/z	m/z error (PPM)
C12H11F2N3O2	99.999999	290.071154	0.396516

Item name: BBS-14
Item description:

Channel name: 1: Average Time 0.0746 min : TOF MS (50-1500) ESI+ : Centroided : Combined

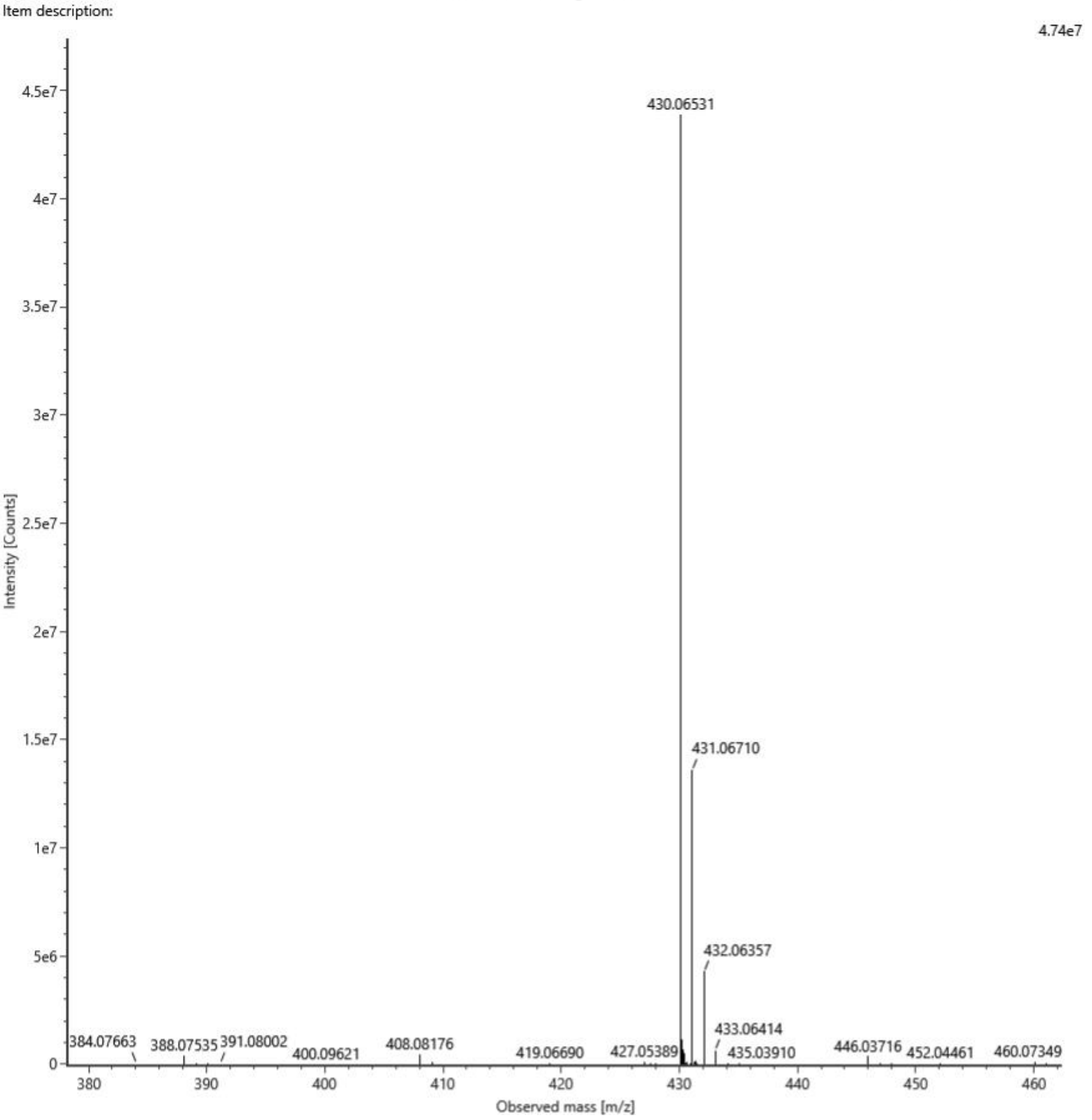

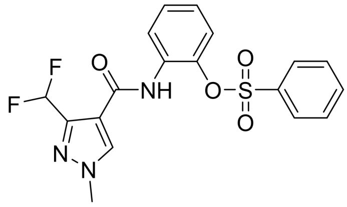

T1

Add:Na+

Composition	i-FIT Confidence (%)	Predicted m/z	m/z error (PPM)
C18H15F2N3O4S	100.000000	430.064354	2.347964

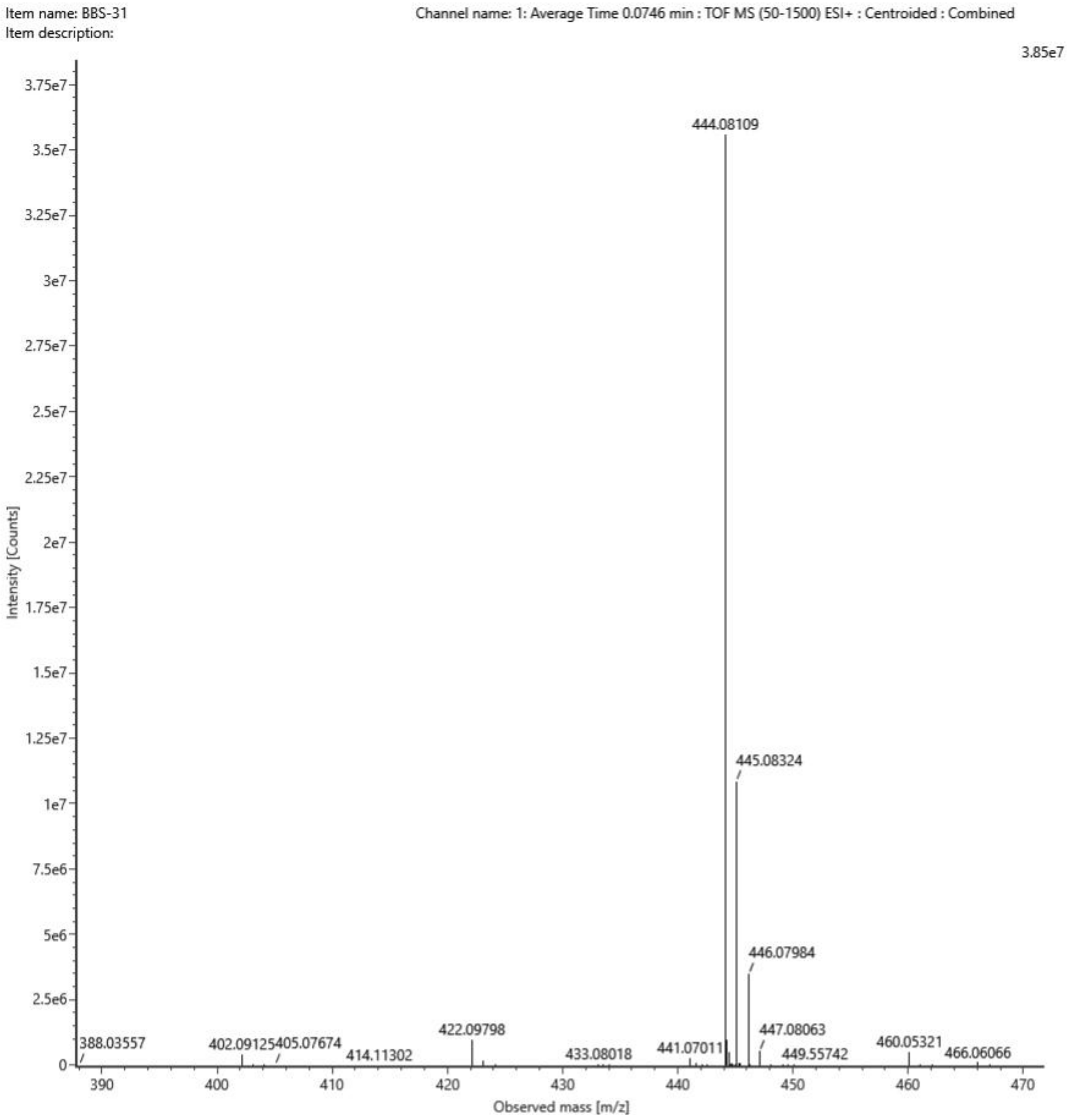

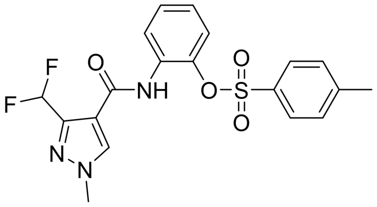

T2

Add:Na+

Composition	i-FIT Confidence (%)	Predicted m/z	m/z error (PPM)
C19H17F2N3O4S	100.000000	444.080004	2.578383

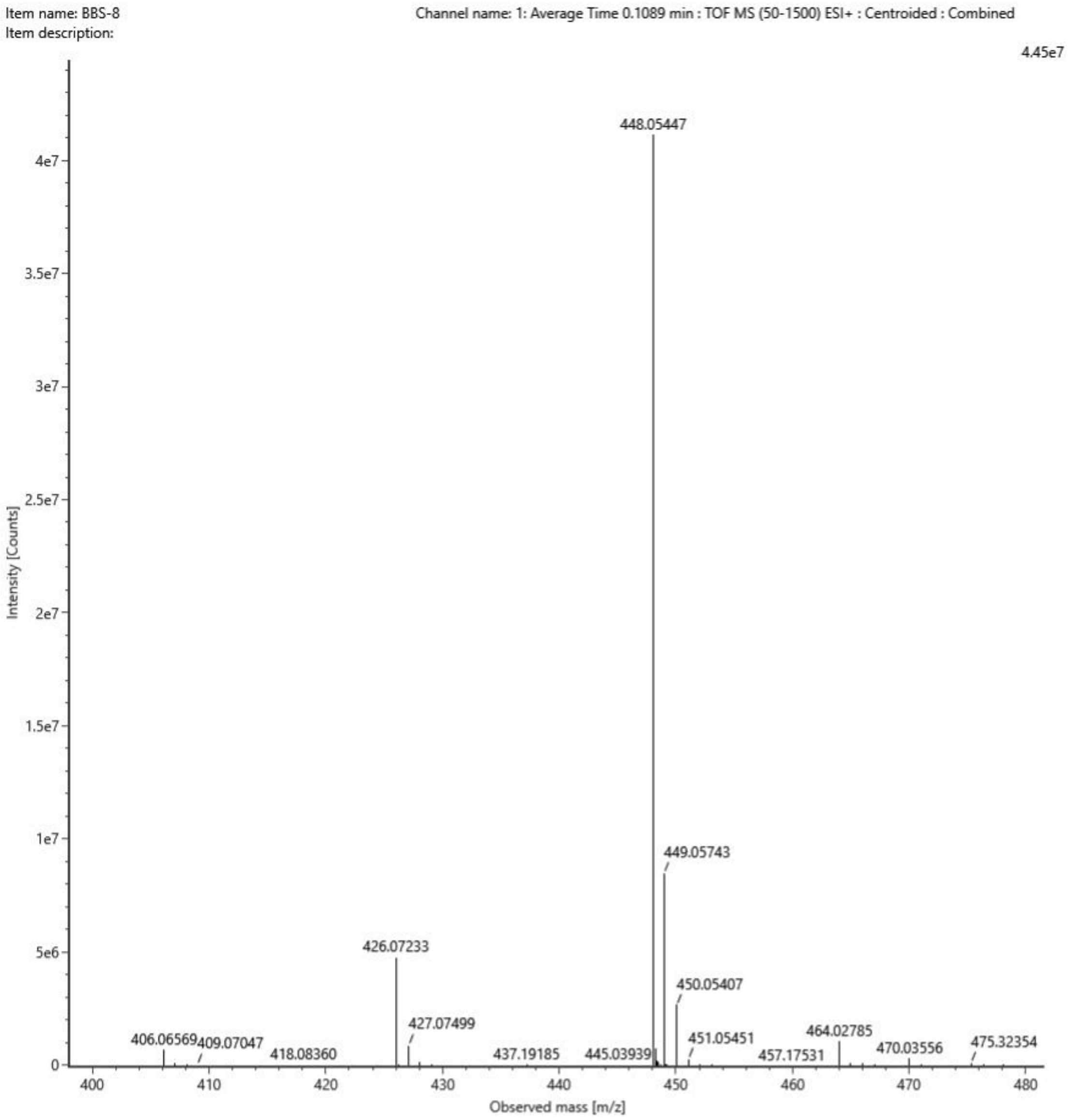

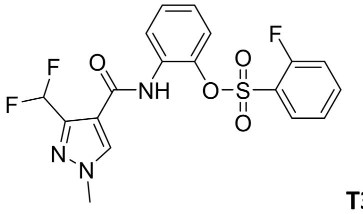

T3

Add:Na+

Composition	i-FIT Confidence (%)	Predicted m/z	m/z error (PPM)
C18H14F3N3O4S	99.695231	448.054932	-1.087758

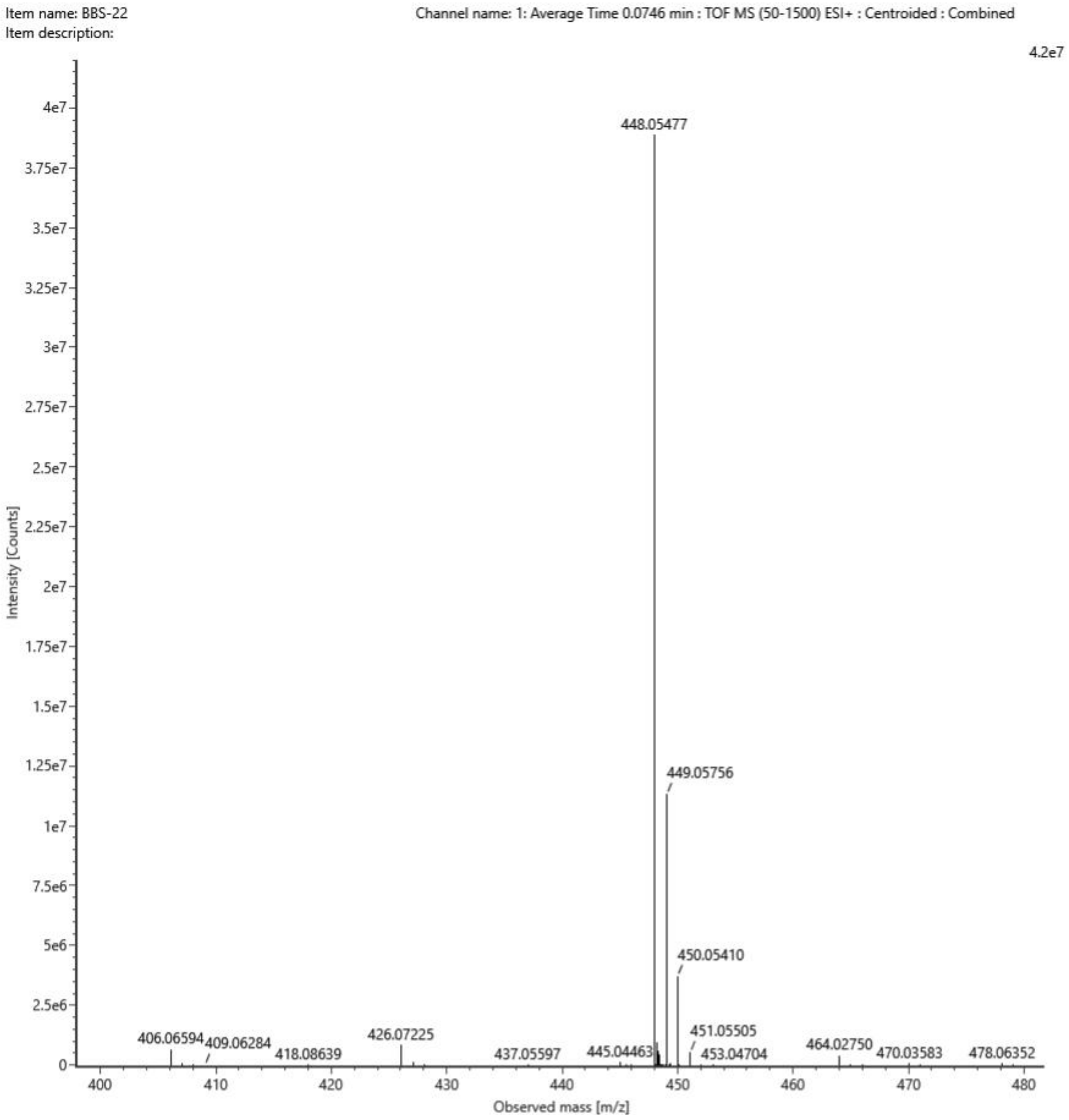

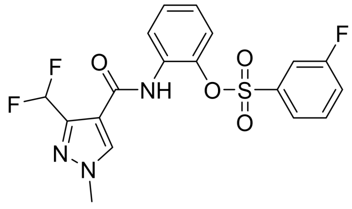

T4

Add:Na+

Composition	i-FIT Confidence (%)	Predicted m/z	m/z error (PPM)
C18H14F3N3O4S	99.887802	448.054932	-0.381984

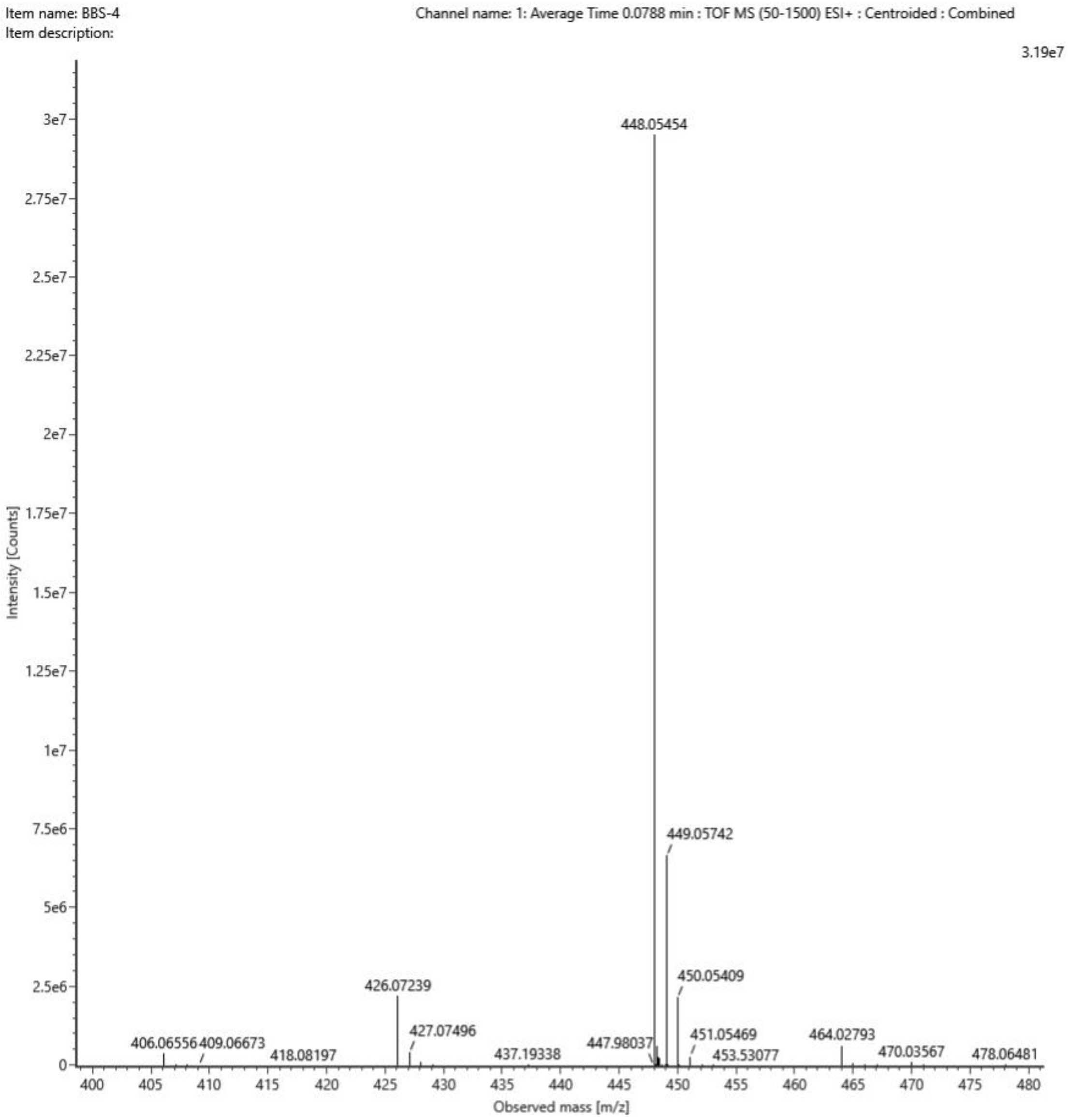

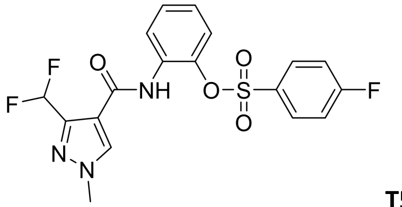

T5

Add:Na+

Composition	i-FIT Confidence (%)	Predicted m/z	m/z error (PPM)
C18H14F3N3O4S	100.000000	448.054932	-0.923077

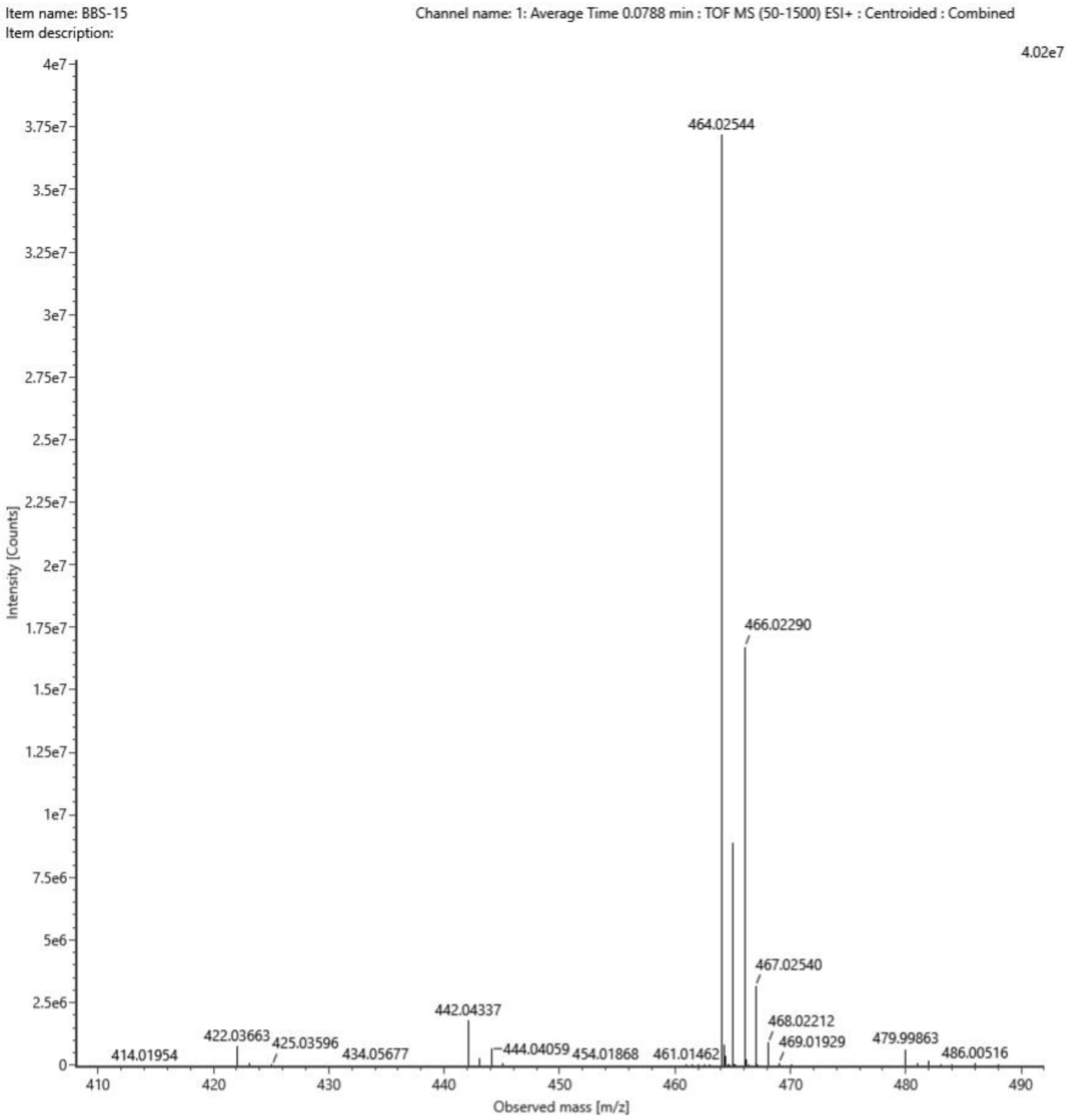

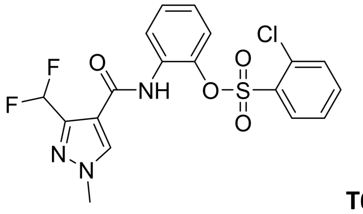

T6

Add:Na+

Composition	i-FIT Confidence (%)	Predicted m/z	m/z error (PPM)
C18H14ClF2N3O4S	100.000000	464.025382	0.131785

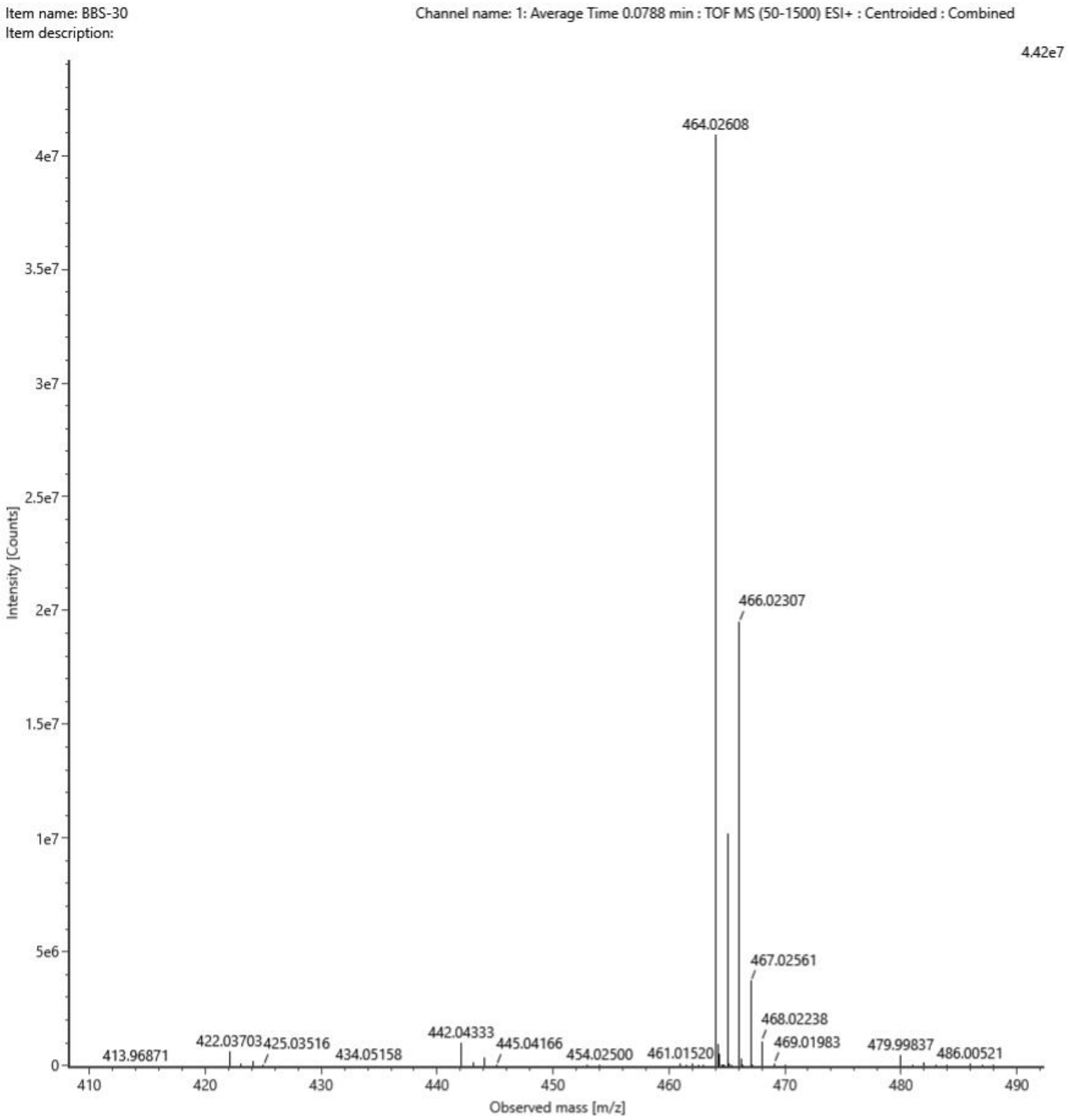

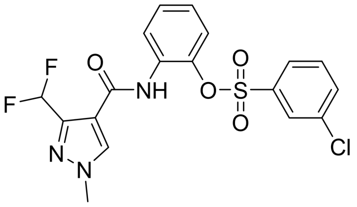

T7

Add:Na+

Composition	i-FIT Confidence (%)	Predicted m/z	m/z error (PPM)
C18H14ClF2N3O4S	99.999995	464.025382	1.582911

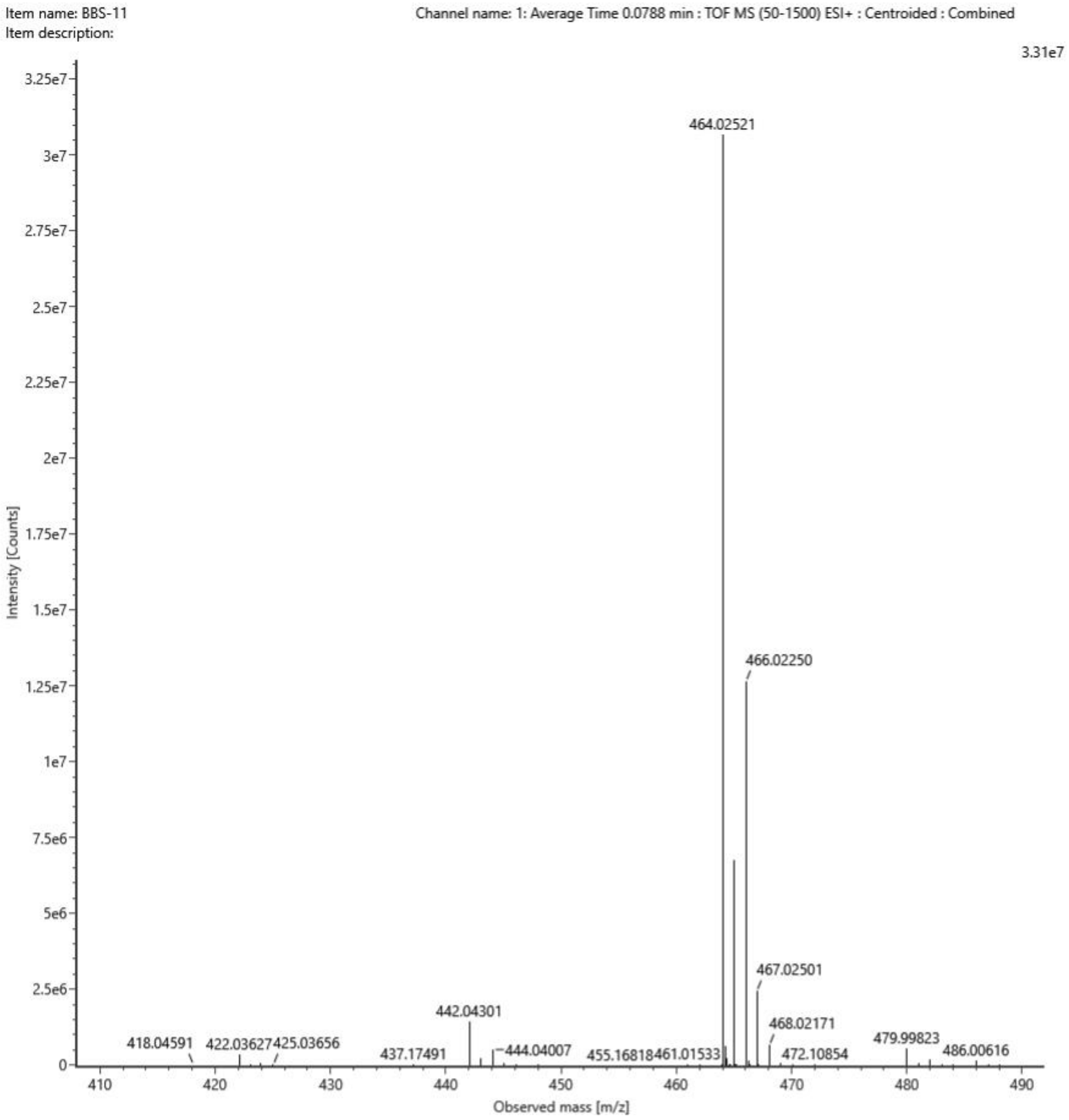

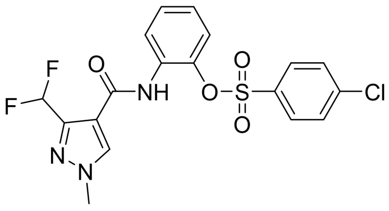

T8

Add:Na+

Composition	i-FIT Confidence (%)	Predicted m/z	m/z error (PPM)
C18H14ClF2N3O4S	100.000000	464.025382	-0.389714

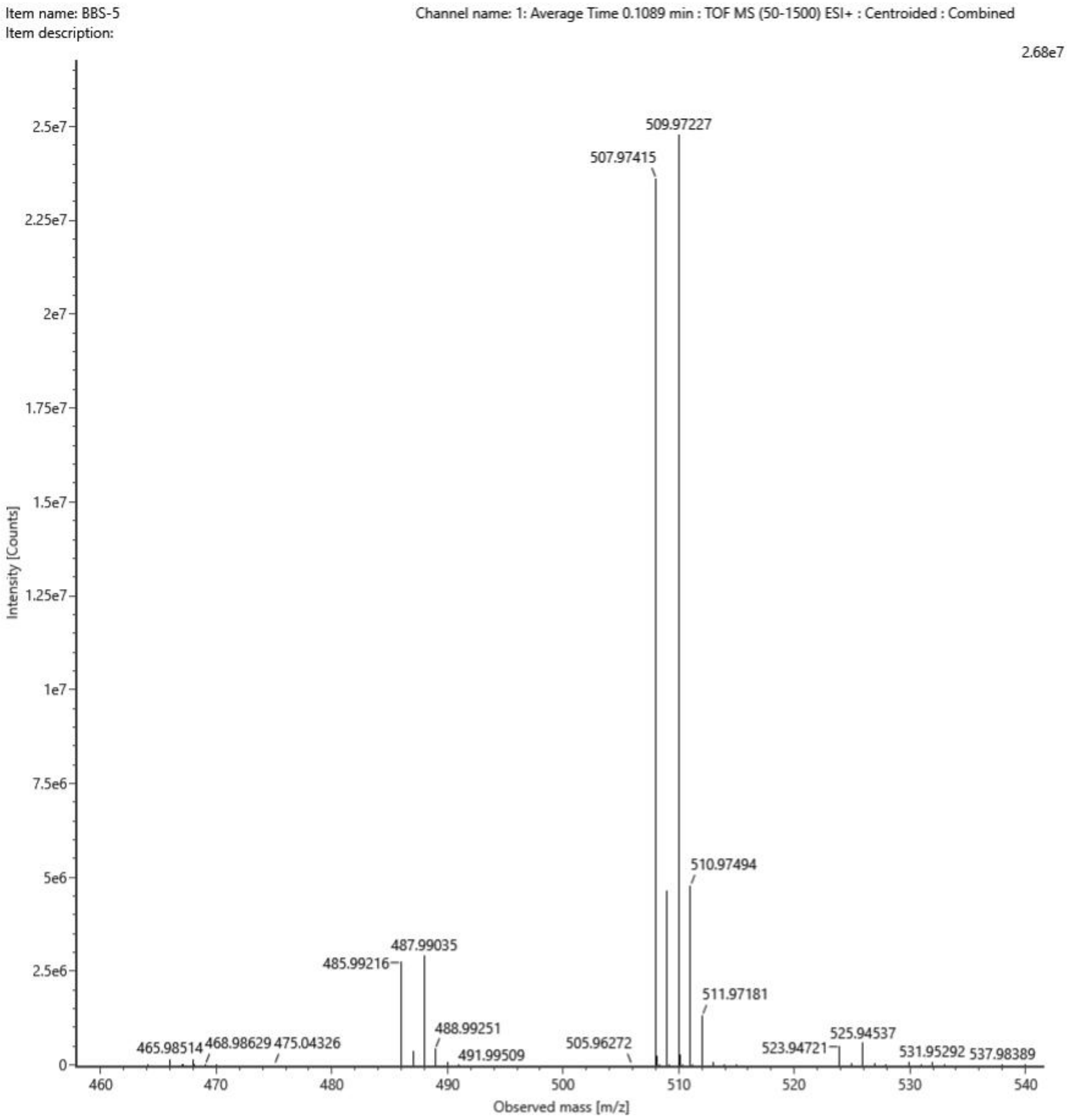

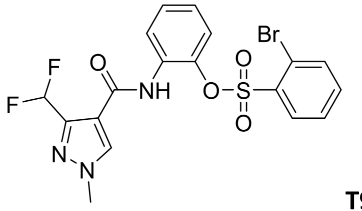

T9

Add:Na+

Composition	i-FIT Confidence (%)	Predicted m/z	m/z error (PPM)
C18H14BrF2N3O4S	100.000000	507.974867	-1.478537

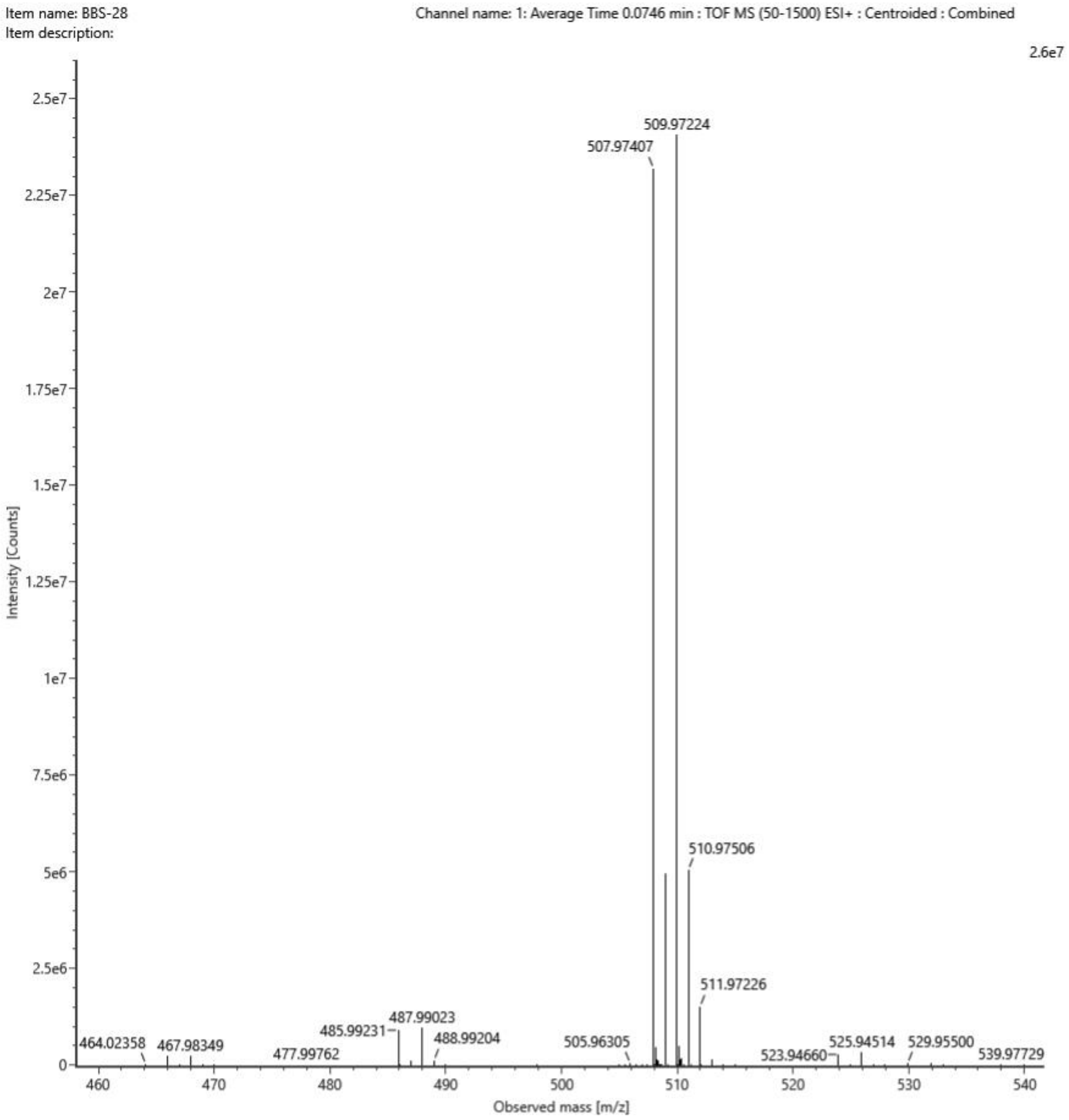

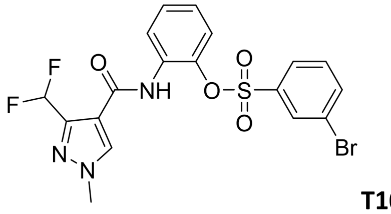

T10

Add:Na+

Composition	i-FIT Confidence (%)	Predicted m/z	m/z error (PPM)
C18H14BrF2N3O4S	100.000000	507.974867	-1.643491

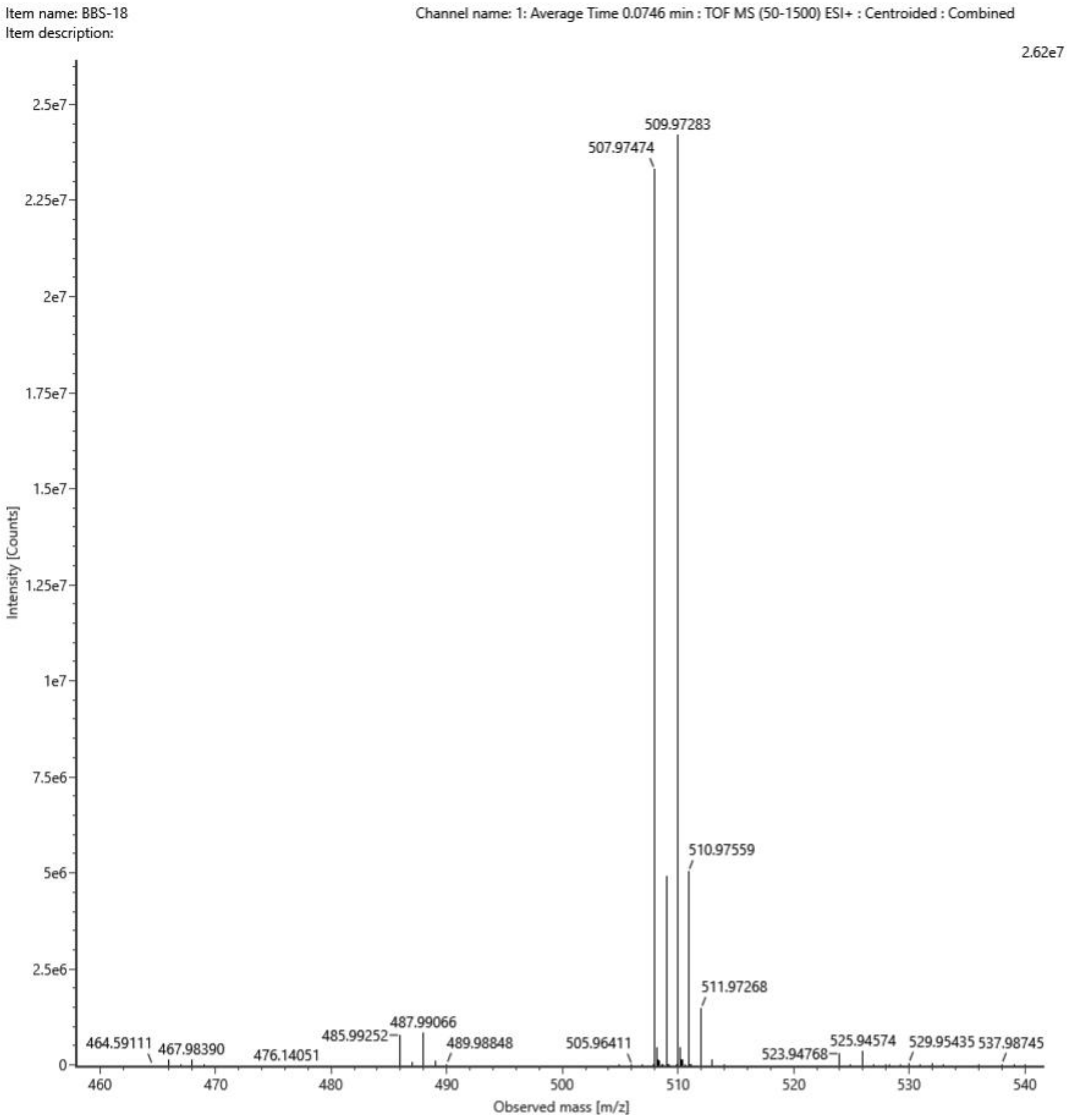

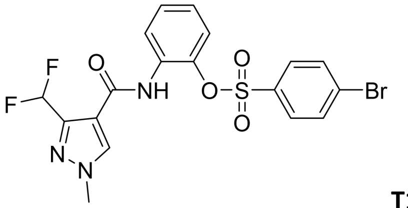

T11

Add:Na+

Composition	i-FIT Confidence (%)	Predicted m/z	m/z error (PPM)
C18H14BrF2N3O4S	99.998923	507.974867	-0.262004

Item name: BBS-17
Item description:

Channel name: 1: Average Time 0.0746 min : TOF MS (50-1500) ESI+ : Centroided : Combined

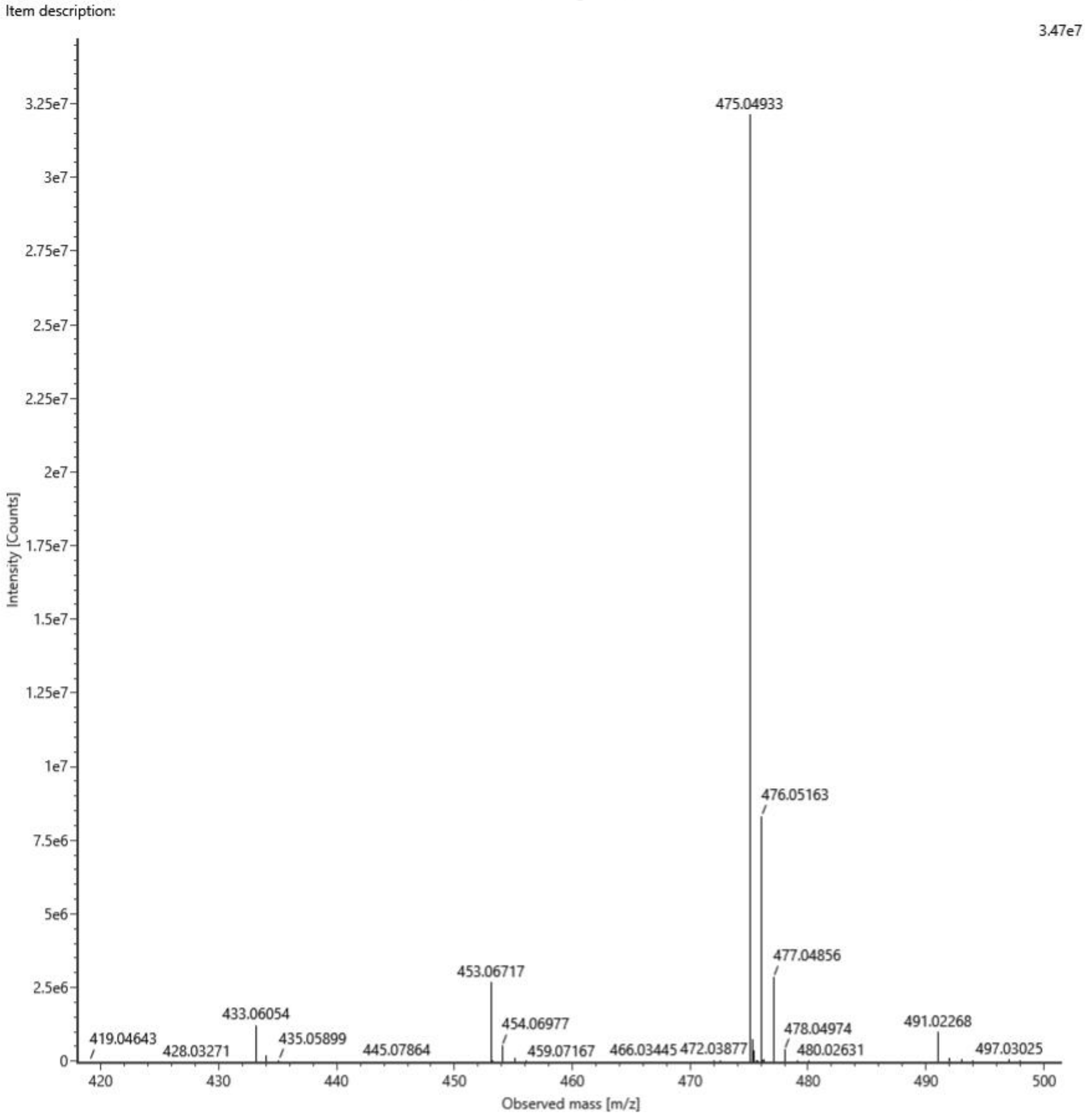

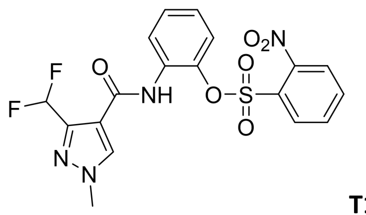

T12

Add:Na+

Composition	i-FIT Confidence (%)	Predicted m/z	m/z error (PPM)
C18H14F2N4O6S	100.000000	475.049432	-0.226563

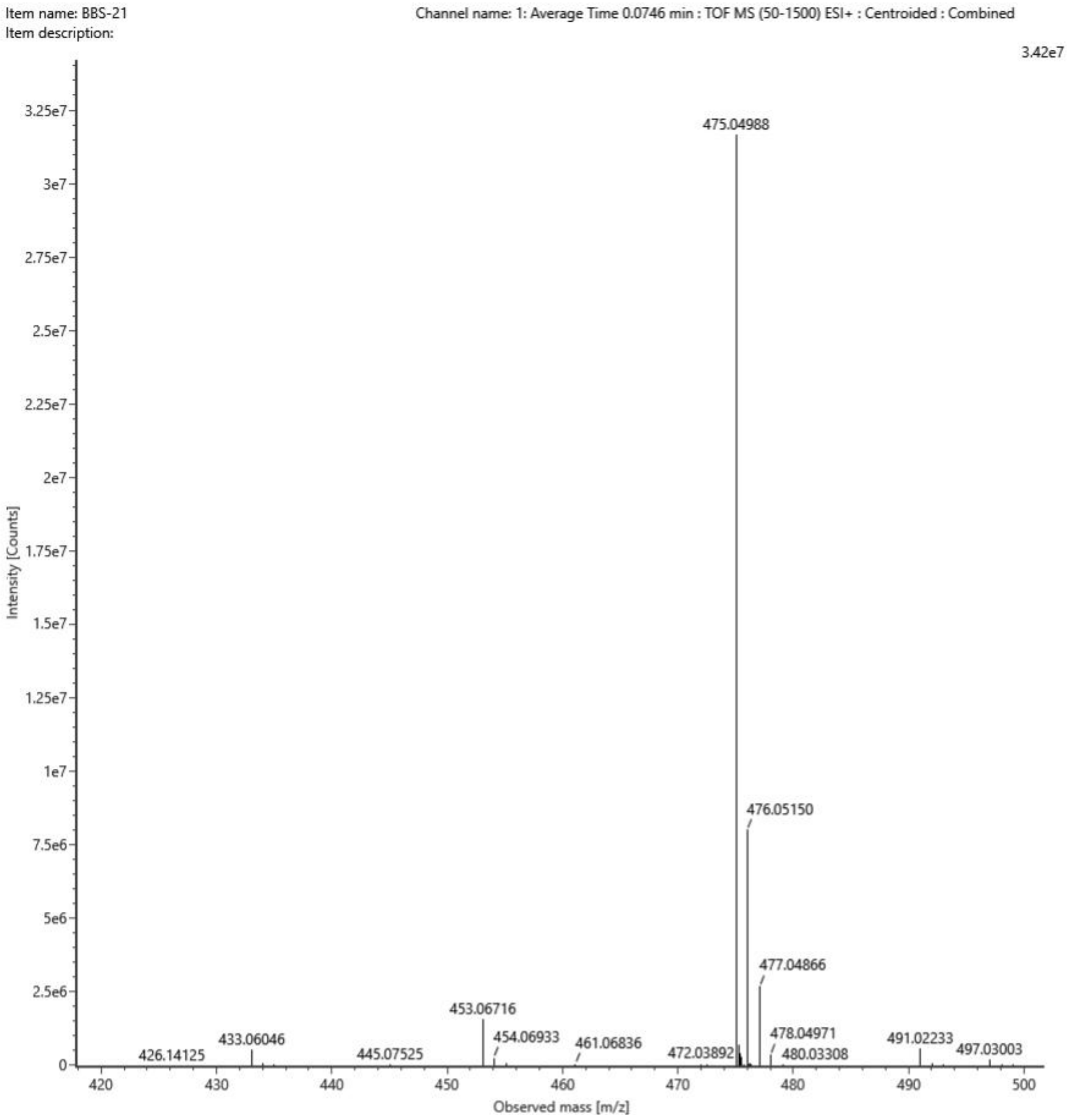

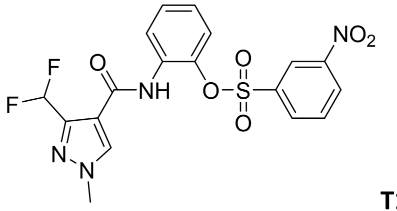

T13

Add:Na+

Composition	i-FIT Confidence (%)	Predicted m/z	m/z error (PPM)
C18H14F2N4O6S	100.000000	475.049432	0.990088

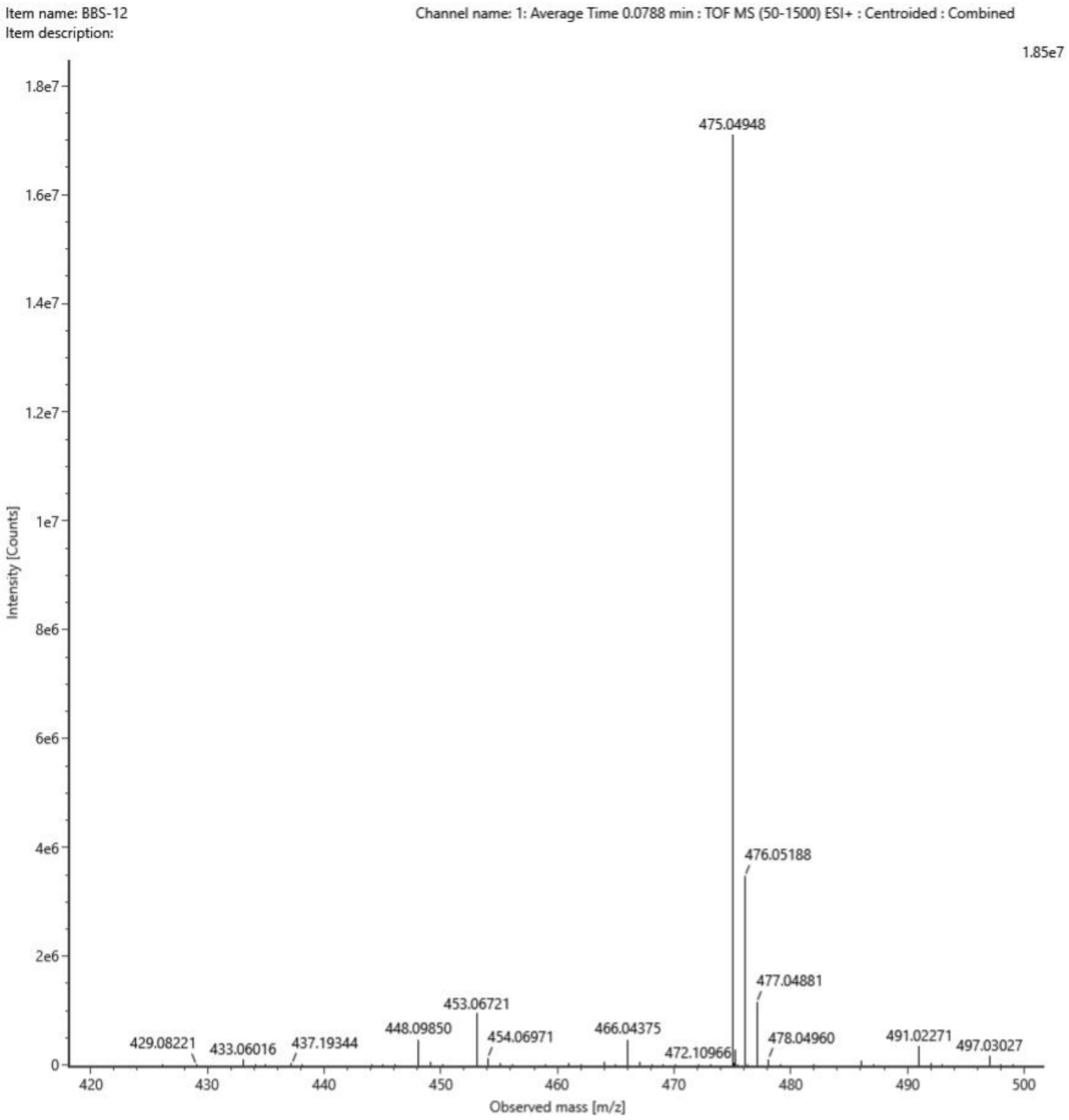

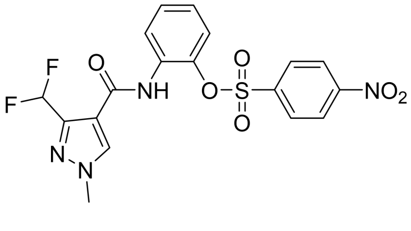

T14

Add:Na+

Composition	i-FIT Confidence (%)	Predicted m/z	m/z error (PPM)
C18H14F2N4O6S	100.000000	475.049432	0.105251

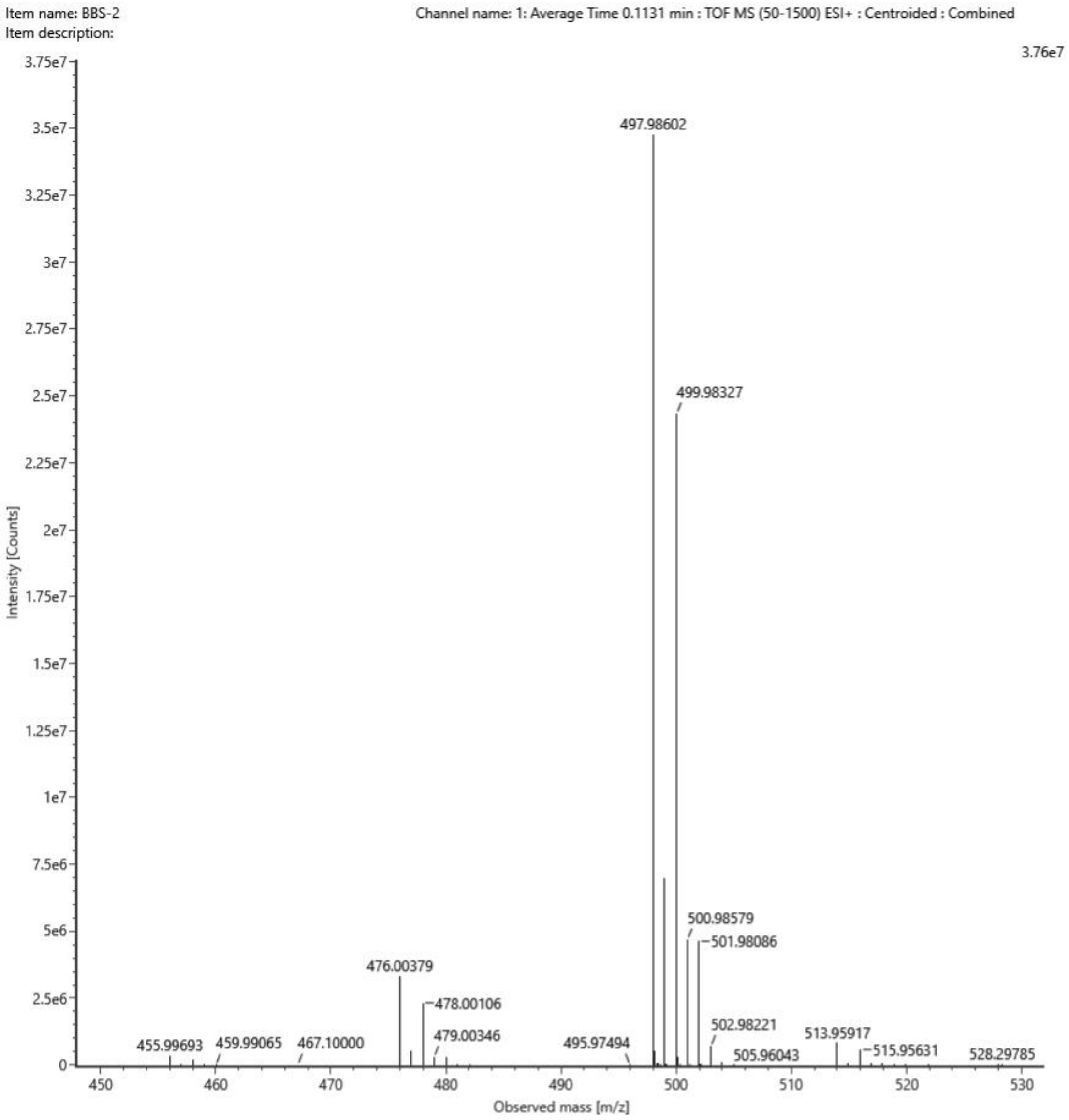

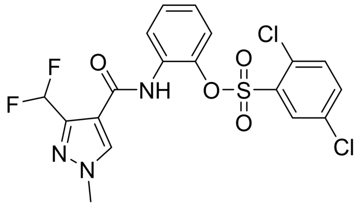

T15

Add:Na+

Composition	i-FIT Confidence (%)	Predicted m/z	m/z error (PPM)
C18H13Cl2F2N3O4S	98.802622	497.986410	-0.820124

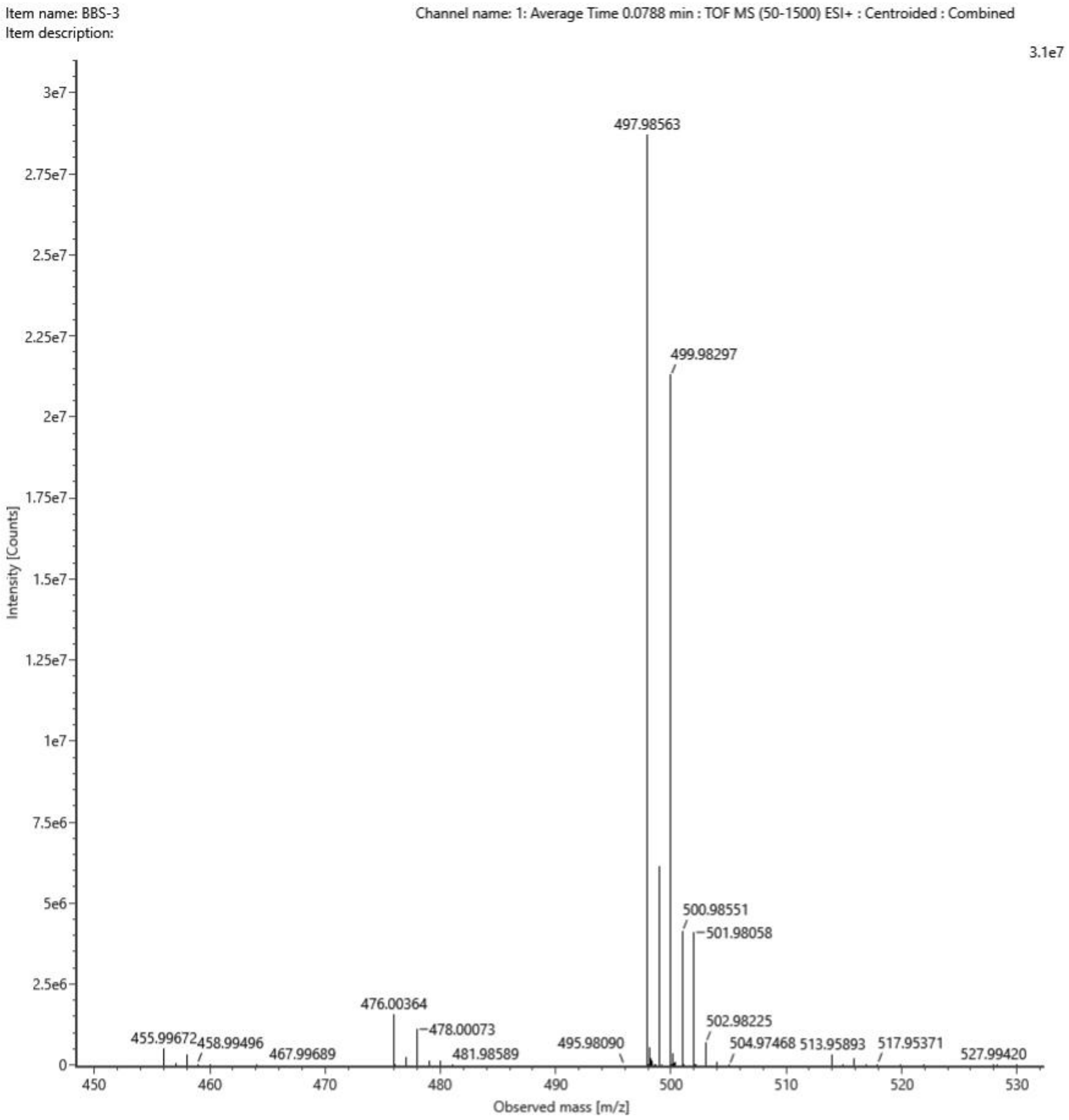

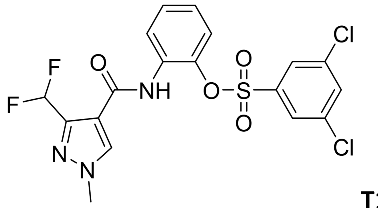

T16

Add:Na+

Composition	i-FIT Confidence (%)	Predicted m/z	m/z error (PPM)
C18H13Cl2F2N3O4S	97.721434	497.986410	-1.641183

Item name: BBS-29
Item description:

Channel name: 1: Average Time 0.1089 min : TOF MS (50-1500) ESI+ : Centroided : Combined

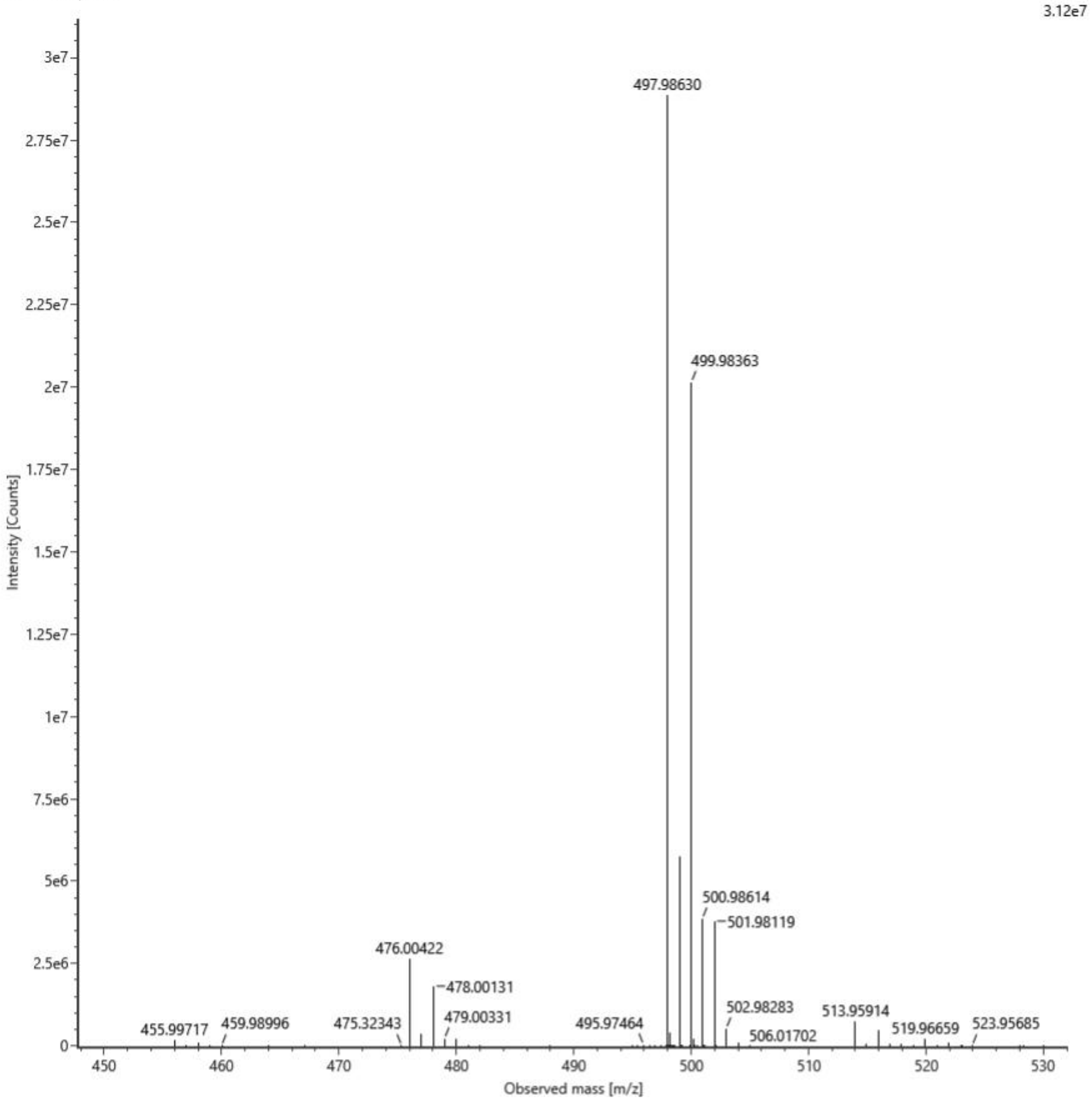

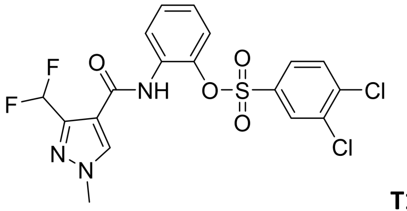

T17

Add:Na+

Composition	i-FIT Confidence (%)	Predicted m/z	m/z error (PPM)
C18H13Cl2F2N3O4S	99.999976	497.986410	-0.230646

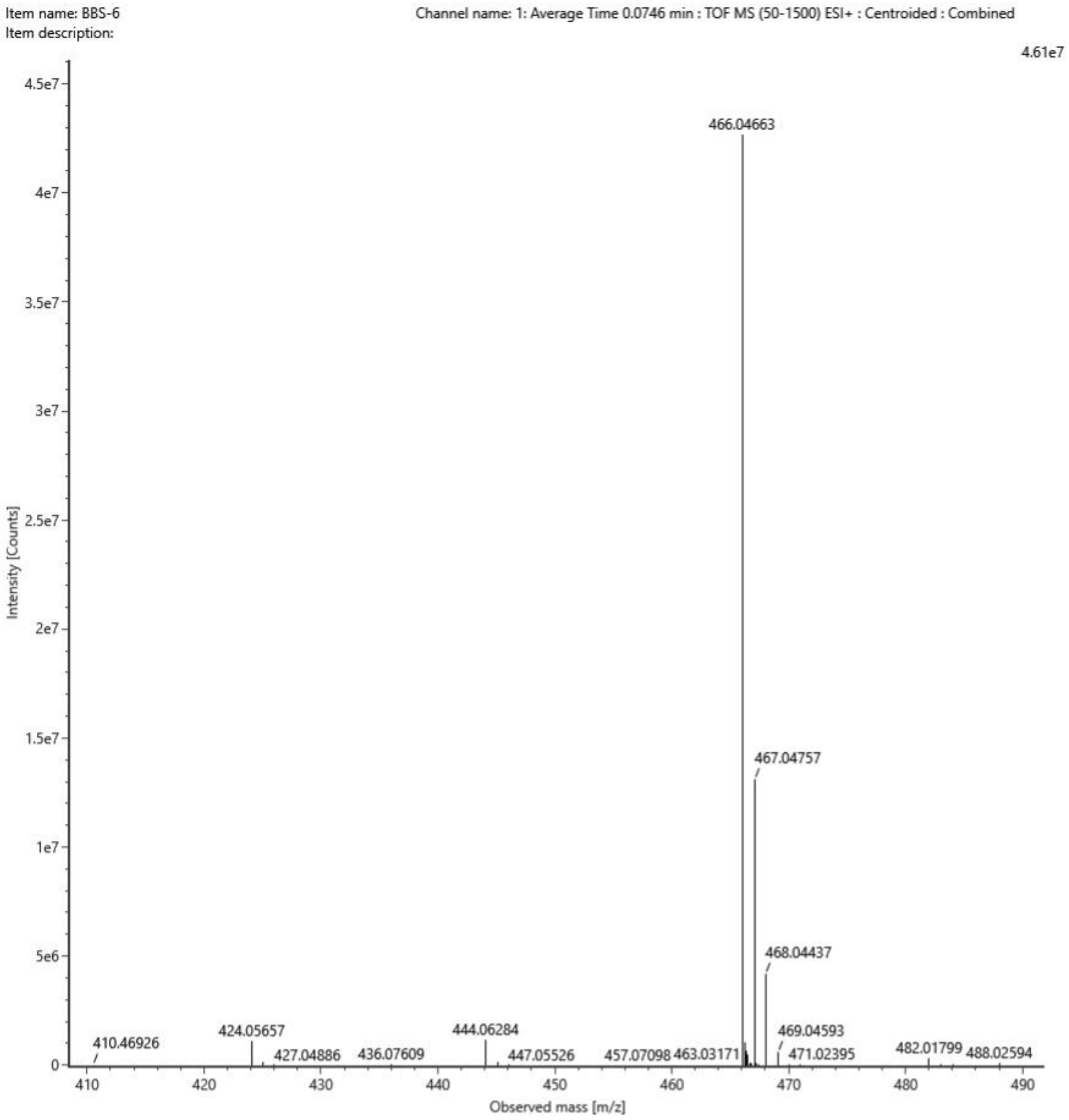

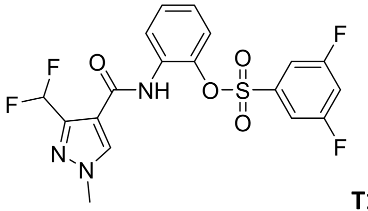

T18

Add:Na+

Composition	i-FIT Confidence (%)	Predicted m/z	m/z error (PPM)
C18H13F4N3O4S	100.000000	466.045511	2.526679

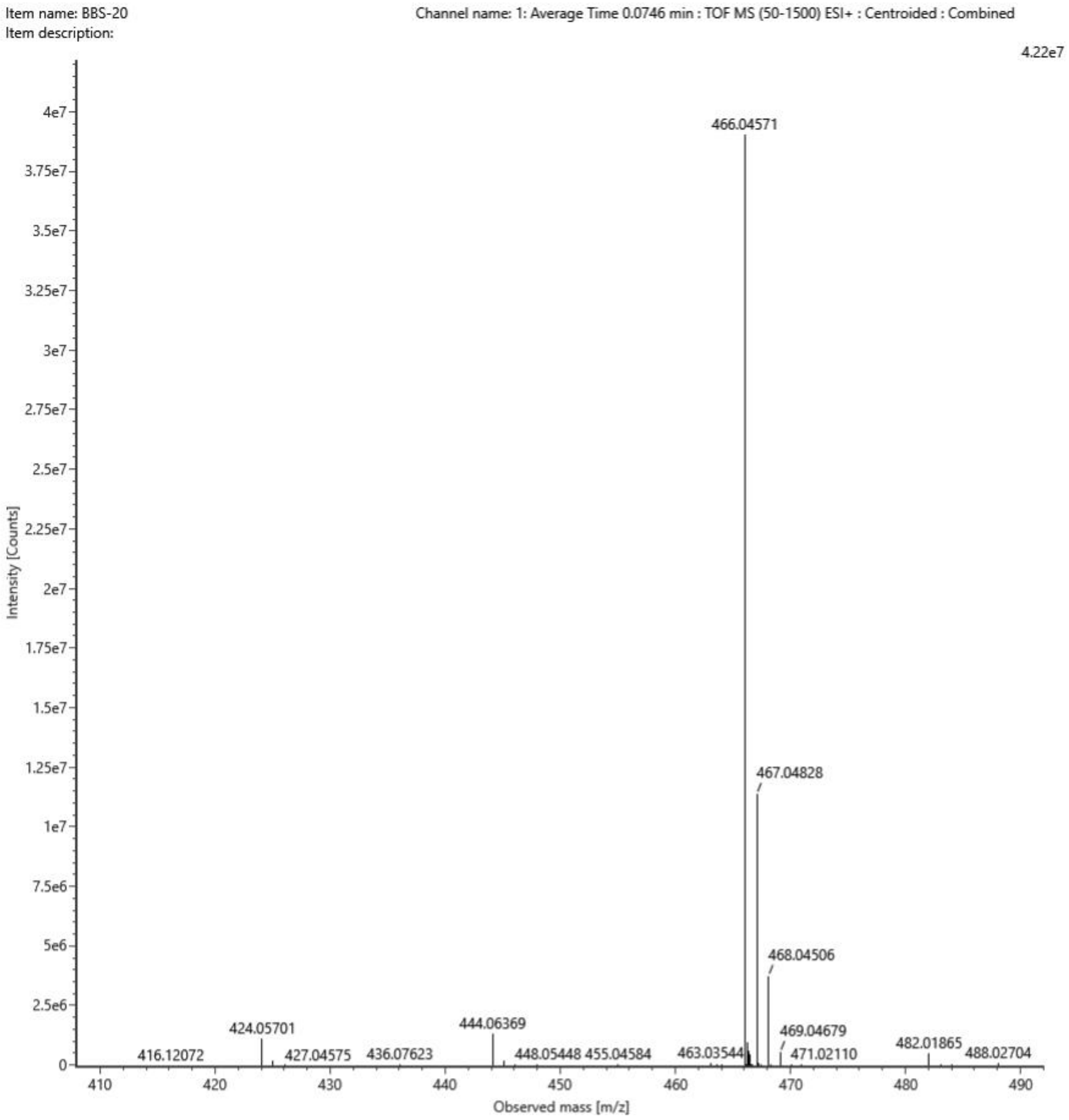

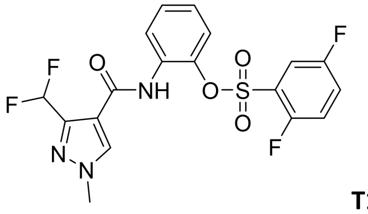

T19

Add:Na+

Composition	i-FIT Confidence (%)	Predicted m/z	m/z error (PPM)
C18H13F4N3O4S	100.000000	466.045511	0.450199

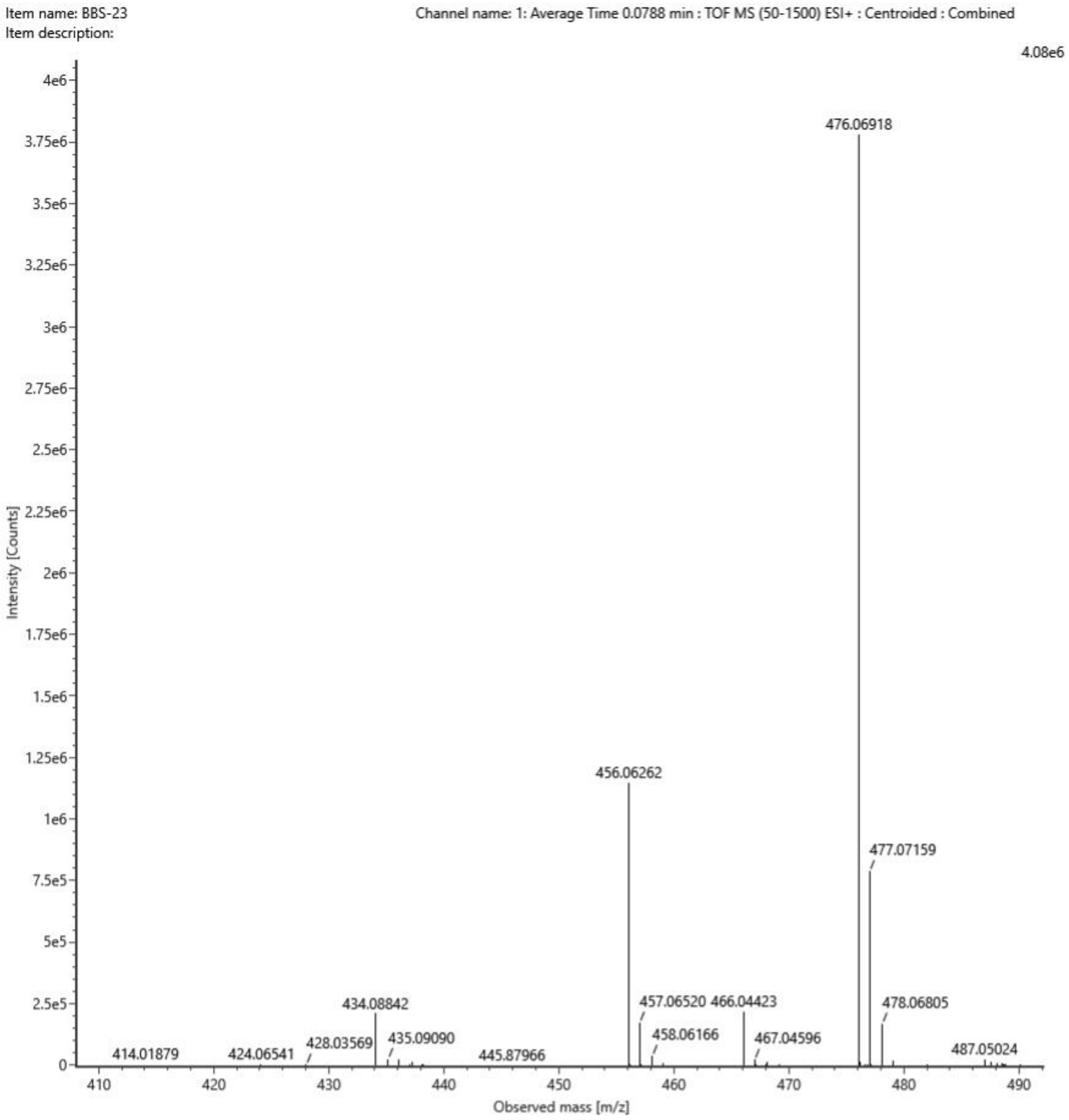

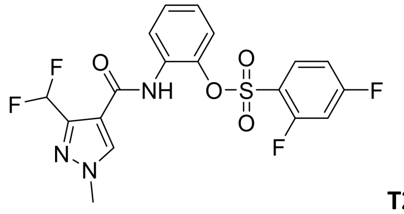

T20

Add:Na+

Composition	i-FIT Confidence (%)	Predicted m/z	m/z error (PPM)
C18H13F4N3O4S	100.000000	466.045511	-2.890242

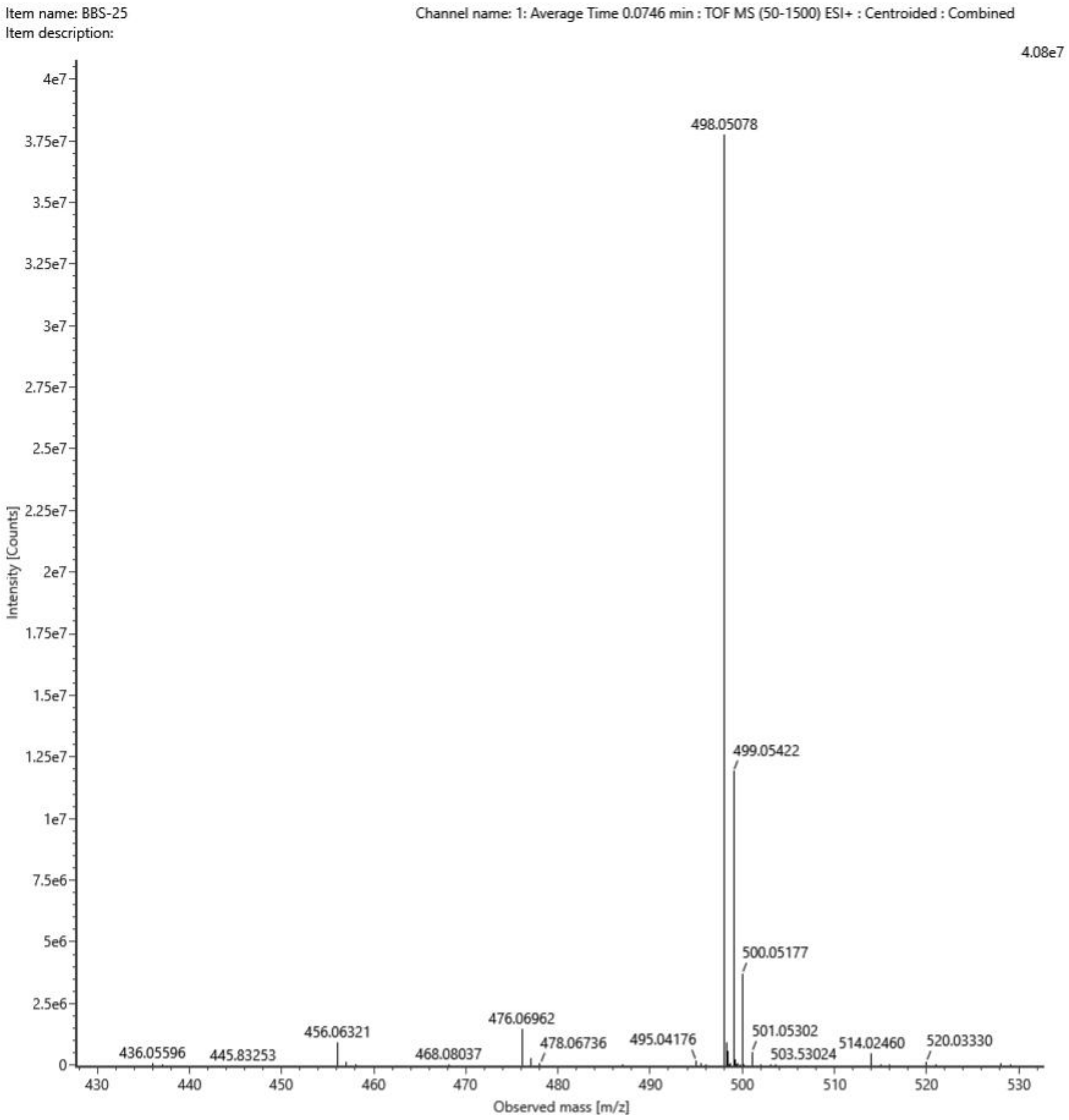

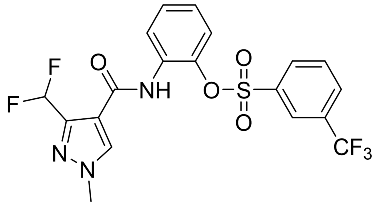

T22

Add:Na+

Composition	i-FIT Confidence (%)	Predicted m/z	m/z error (PPM)
C19H14F5N3O4S	100.000000	498.051739	-2.018198

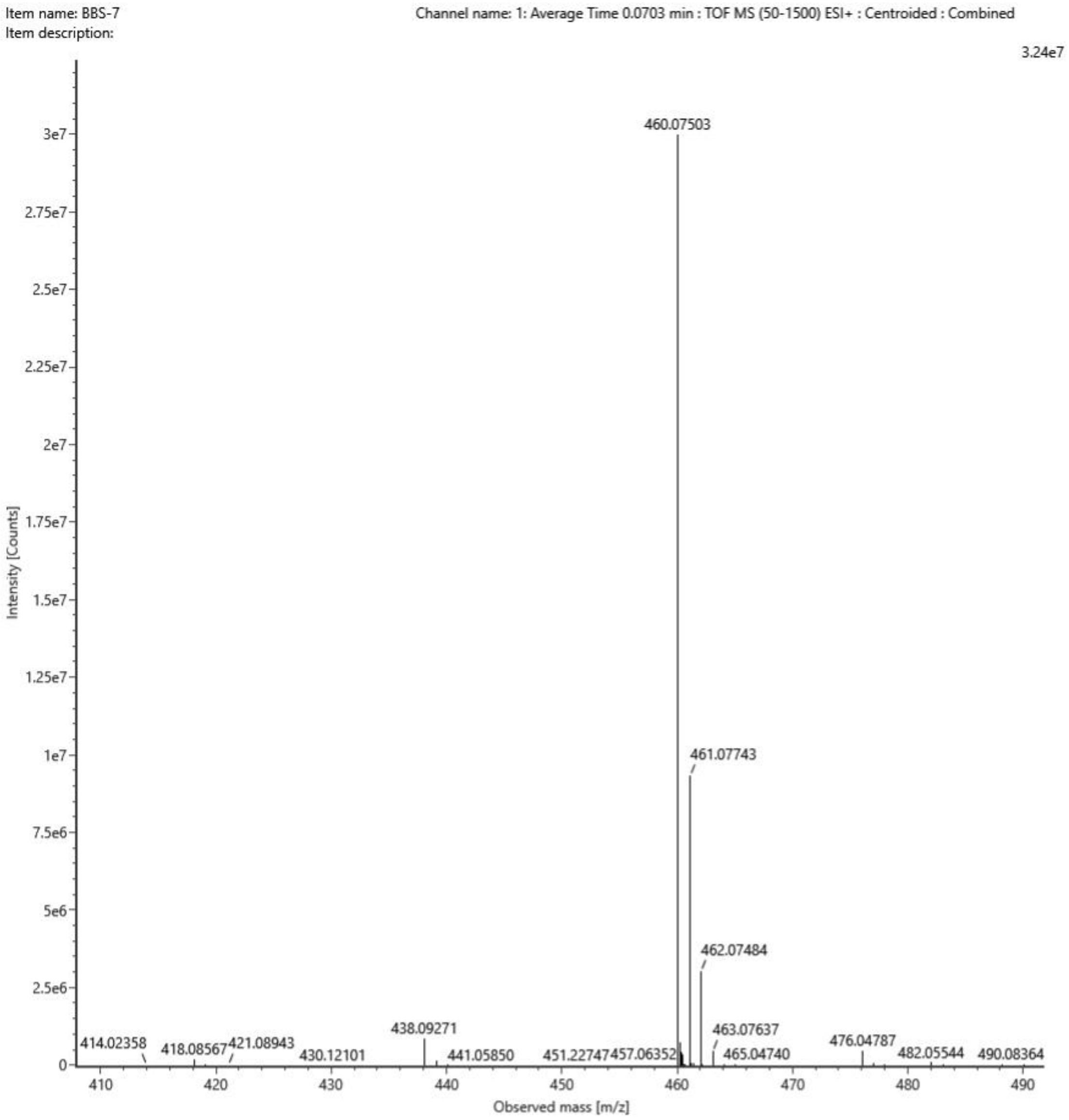

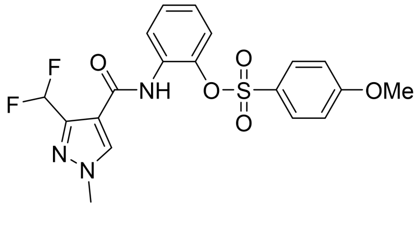

T23

Add:Na+

Composition	i-FIT Confidence (%)	Predicted m/z	m/z error (PPM)
C19H17F2N3O5S	99.738518	460.074919	0.254215

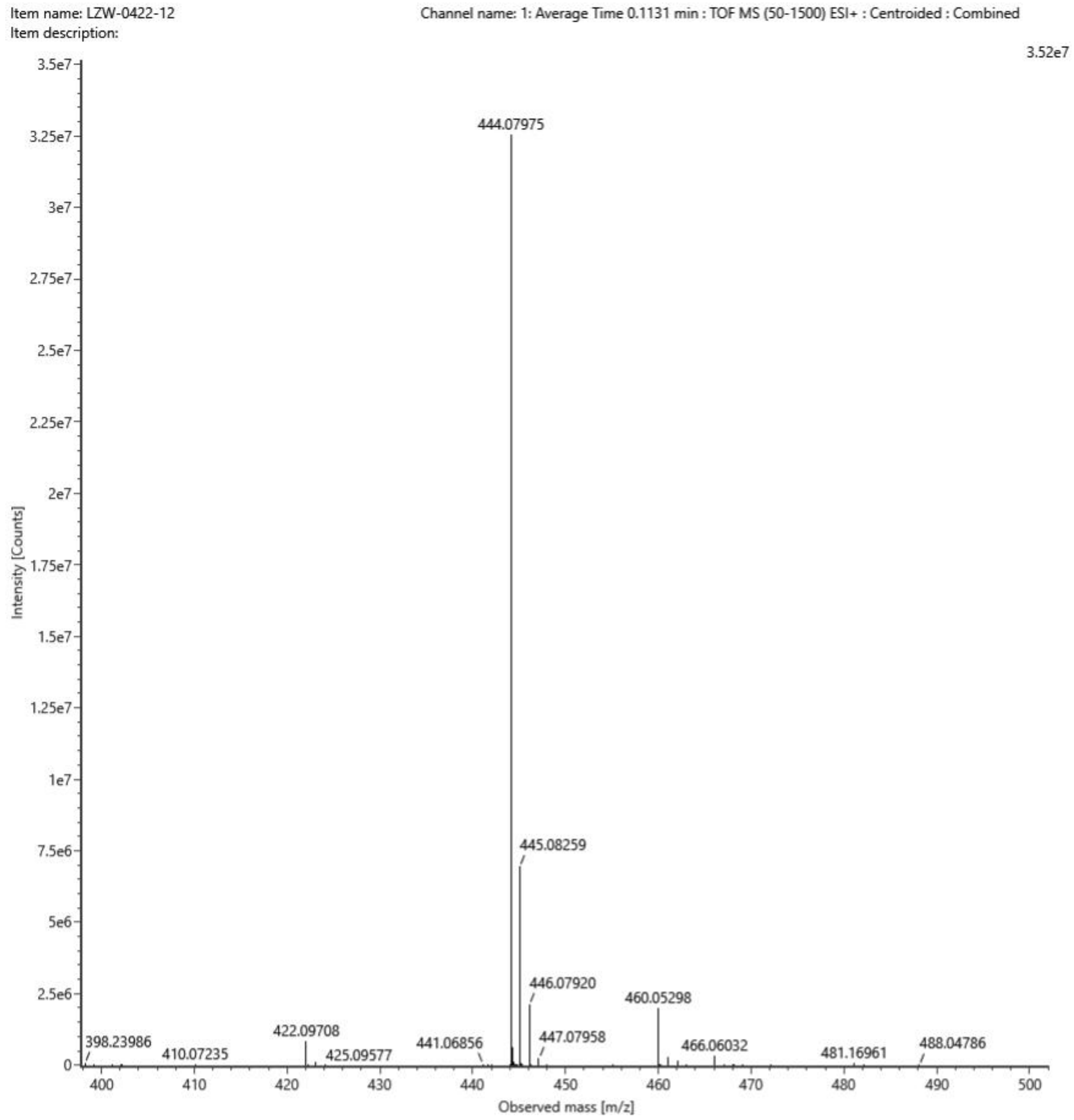

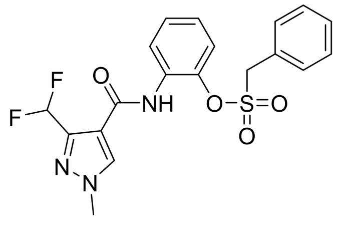

T24

Add:Na+

Composition	i-FIT Confidence (%)	Predicted m/z	m/z error (PPM)
C19H17F2N3O4S	100.000000	444.080004	-0.573865

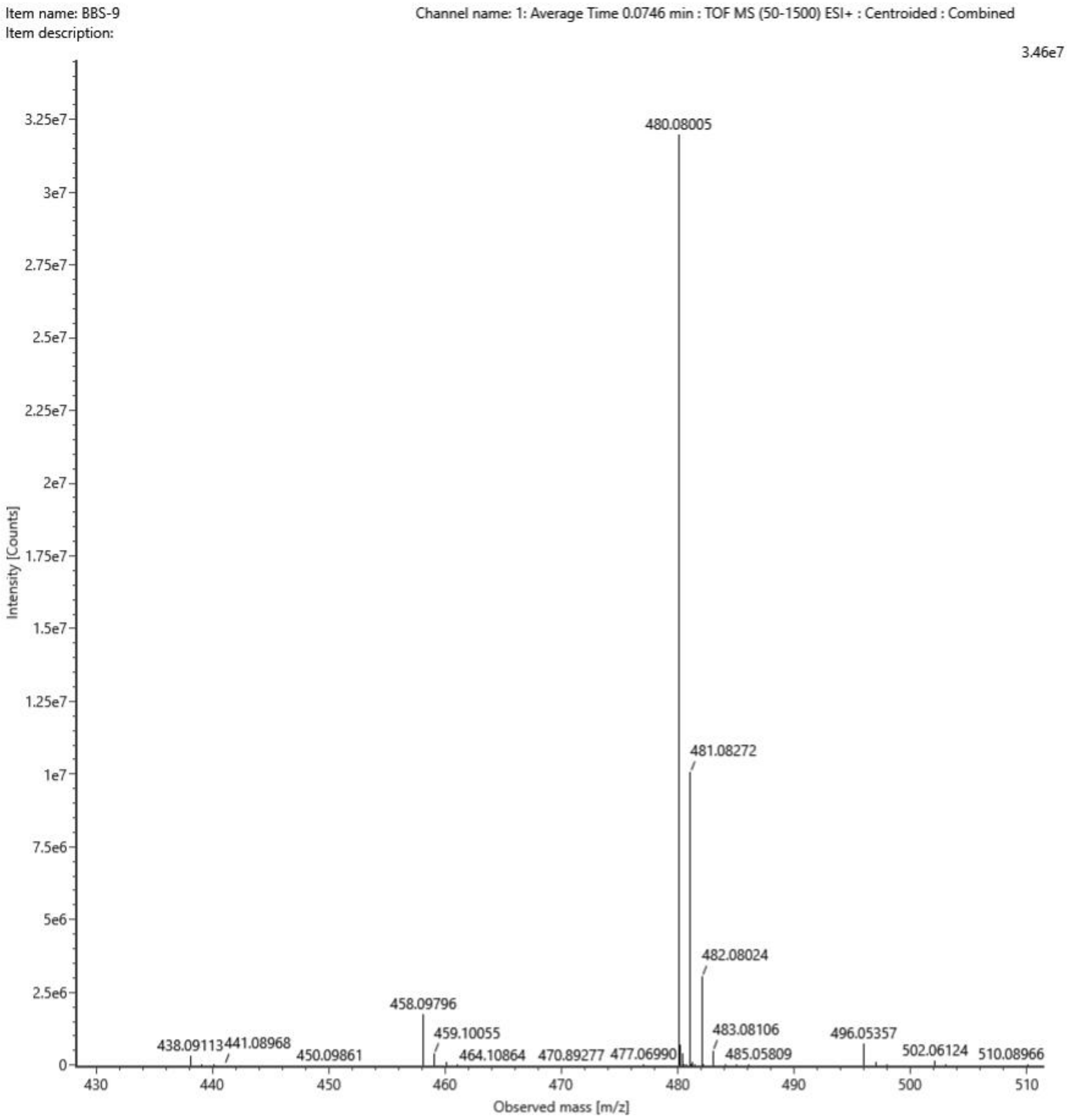

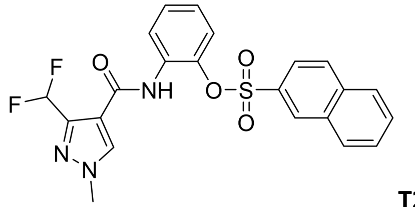

T25

Add:Na+

Composition	i-FIT Confidence (%)	Predicted m/z	m/z error (PPM)
C22H17F2N3O4S	100.000000	480.080004	0.100059

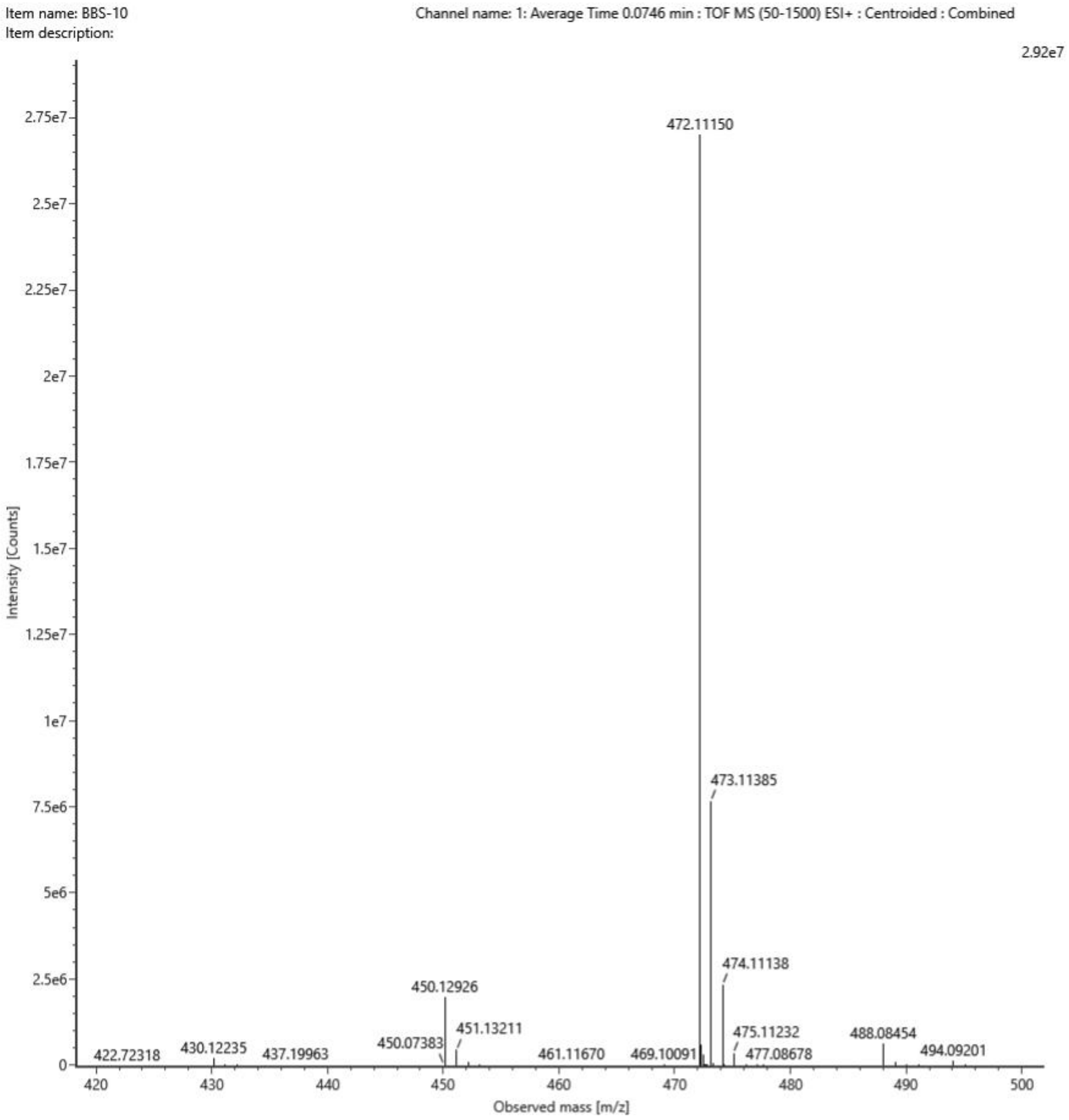

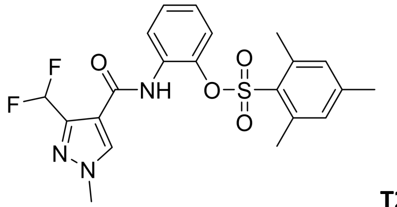

T26

Add:Na+

Composition	i-FIT Confidence (%)	Predicted m/z	m/z error (PPM)
C21H21F2N3O4S	100.000000	472.111304	0.435535

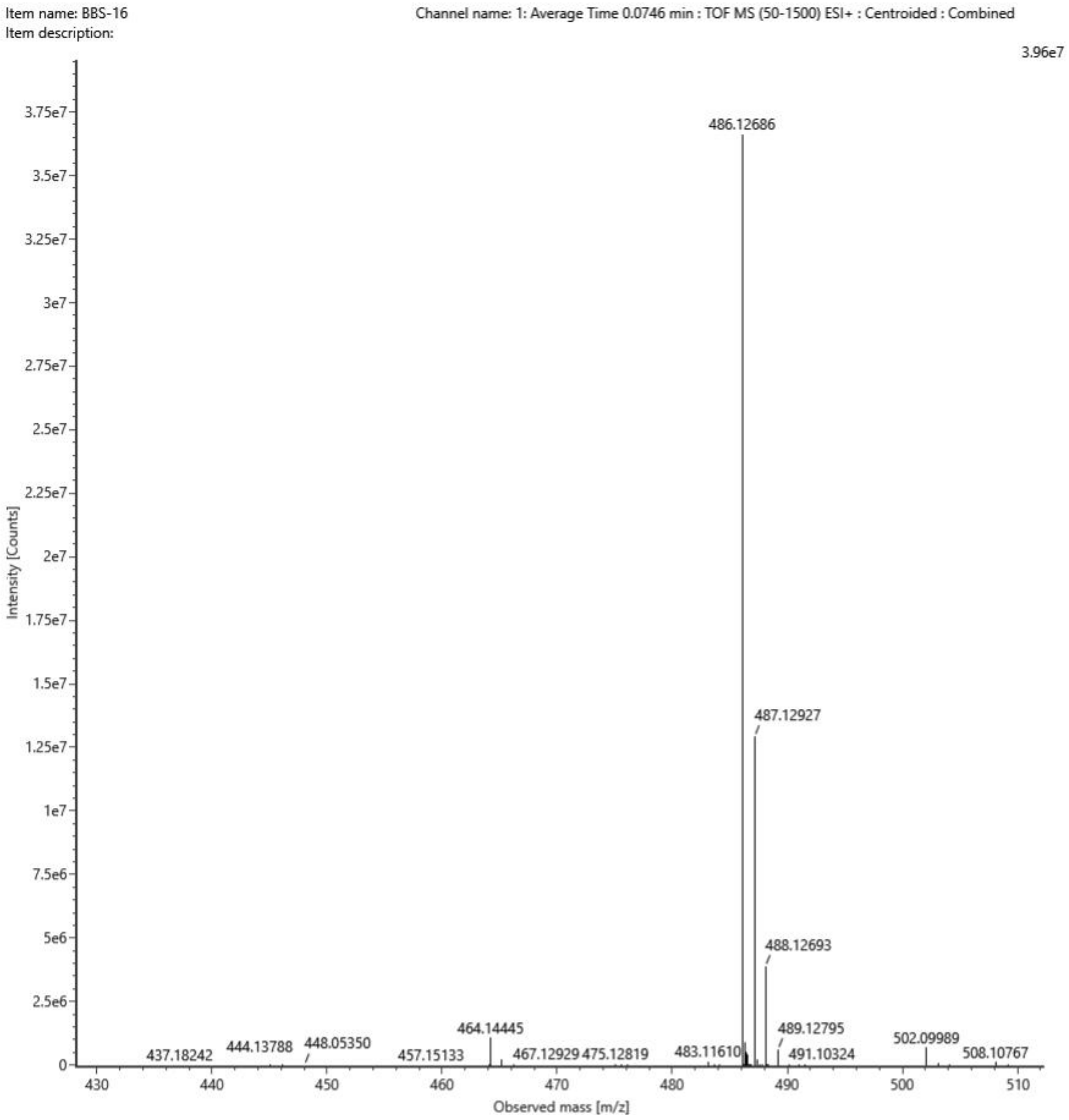

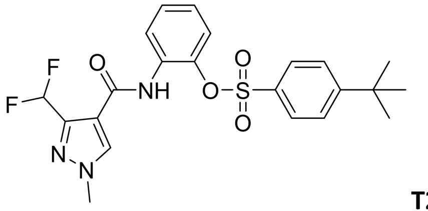

T27

Add:Na+

Composition	i-FIT Confidence (%)	Predicted m/z	m/z error (PPM)
C22H23F2N3O4S	99.999999	486.126954	-0.203946
